# Altering Pyrroloquinoline Quinone Nutritional Status Modulates Mitochondrial, Lipid, and Energy Metabolism in Rats

**DOI:** 10.1371/journal.pone.0021779

**Published:** 2011-07-21

**Authors:** Kathryn Bauerly, Calliandra Harris, Winyoo Chowanadisai, James Graham, Peter J. Havel, Eskouhie Tchaparian, Mike Satre, Joel S. Karliner, Robert B. Rucker

**Affiliations:** 1 Nutrition, University of California Davis, Davis, California, United States of America; 2 Molecular Biosciences, Veterinary Medicine, University of California Davis, Davis, California, United States of America; 3 Amgen Inc., South San Francisco, California, United States of America; 4 Cardiology, VA Medical Center, San Francisco, California, United States of America; New Mexico State University, United States of America

## Abstract

We have reported that pyrroloquinoline quinone (PQQ) improves reproduction, neonatal development, and mitochondrial function in animals by mechanisms that involve mitochondrial related cell signaling pathways. To extend these observations, the influence of PQQ on energy and lipid relationships and apparent protection against ischemia reperfusion injury are described herein. Sprague-Dawley rats were fed a nutritionally complete diet with PQQ added at either 0 (PQQ−) or 2 mg PQQ/Kg diet (PQQ+). Measurements included: 1) serum glucose and insulin, 2) total energy expenditure per metabolic body size (Wt^3/4^), 3) respiratory quotients (in the fed and fasted states), 4) changes in plasma lipids, 5) the relative mitochondrial amount in liver and heart, and 6) indices related to cardiac ischemia. For the latter, rats (PQQ− or PQQ+) were subjected to left anterior descending occlusions followed by 2 h of reperfusion to determine PQQ's influence on infarct size and myocardial tissue levels of malondialdehyde, an indicator of lipid peroxidation. Although no striking differences in serum glucose, insulin, and free fatty acid levels were observed, energy expenditure was lower in PQQ− vs. PQQ+ rats and energy expenditure (fed state) was correlated with the hepatic mitochondrial content. Elevations in plasma di- and triacylglyceride and β-hydroxybutryic acid concentrations were also observed in PQQ− rats vs. PQQ+ rats. Moreover, PQQ administration (i.p. at 4.5 mg/kg BW for 3 days) resulted in a greater than 2-fold decrease in plasma triglycerides during a 6-hour fast than saline administration in a rat model of type 2 diabetes. Cardiac injury resulting from ischemia/reperfusion was more pronounced in PQQ− rats than in PQQ+ rats. Collectively, these data demonstrate that PQQ deficiency impacts a number of parameters related to normal mitochondrial function.

## Introduction

Although pyrroloquinoline quinone (PQQ) functions as a bacterial enzymatic cofactor, a role in eukaryotic metabolism remains to be fully elucidated. PQQ is present in plant and animal cells and many foods and biological fluids, such as milk, at pM to nM levels [1]–[3]. PQQ has been shown to function as an antioxidant [4]–[9], cardio- and neuroprotectant [6], [9]–[13], and has been demonstrated to act as a cell and plant growth factor for a variety of organisms [14], [15].

In animal models, dietary PQQ deprivation results in abnormal development, immune dysfunction and decreased reproductive performance [16]–[18]. Our previous observations using gene array analysis to assess PQQ nutritional status suggested that PQQ affects a wide range of genes, most notably genes involved in mitochondrial-related functions [19]. For example, changes in gene transcriptional networks indicate that 2–4 percent of the total genes respond to changes in PQQ status depending on dietary conditions or pharmacologic administration. The genes that respond are largely revert to normal levels upon PQQ repletion, and are associated with cellular stress, mitochondriogenesis, and cell signaling. Moreover, we have observed [20] that exposure of mouse Hepa1–6 cells to PQQ results in the activation of cAMP response element-binding protein (CREB) and peroxisome proliferator-activated receptor-gamma-coactivator-1α (PGC-1α). PQQ exposure also increases the levels of nuclear respiratory factor activation (NRF-1 and NRF-2) and Tfam [20]. Such mechanistic features are in keeping with our prior reports that dietary PQQ deficiency results in a decrease in mitochondria amount or number [21]. PQQ-deficient mice and rats have 20–30% reductions in the relative amount of mitochondria in liver, lower respiratory control ratios, and lower respiratory quotients (RQ) than PQQ-supplemented mice [21]. As a consequence of decreased mitochondria, rats also have defects in amino acid metabolism, particularly lysine and other amino acids metabolized primarily in the mitochondria [16].

Given these observations, we hypothesized that rats fed PQQ deficient diets should also display altered lipid clearance and energy metabolism. Accordingly, non-diabetic Sprague-Dawley or diabetic UCD-T2DM rats [22] were fed either a PQQ adequate or PQQ deficient diet and changes in plasma lipid profiles, responses to a glucose challenge were measured, and 24 hour energy expenditures and respiratory quotient (RQ) were measured. As a functional test, the response to cardiac ischemic injury was tested owing to the importance of optimal mitochondrial and fatty acid clearance to minimizing ischemic damage.

## Results

### Animals, Plasma and Tissue PQQ levels, and Mitochondria

The levels of PQQ in plasma, liver, and heart and the relative mitochondrial content in liver and heart (based on changes in the mtDNA/nuclearDNA ratio) are presented in **Table 1** **.** Values for plasma PQQ were markedly reduced when rats were fed PQQ- diets. Short-term repletion with PQQ (3d) rapidly increased plasma PQQ levels in rats deprived of PQQ. Rats designated as adults were beyond sexual maturation (>8 weeks in age) and weighed 225–250 grams. As reported previously, PQQ deprivation did not cause dynamic changes in body weight as rats or mice approach sexual maturation [17]–[19]. The rate of weight gain of rats fed the amino acid based diet was about 90 percent that reported previously for rats fed commercial laboratory chow diets [19].

**Table 1:** Plasma PQQ and mtDNA/nuclear DNA Ratio Levels.

PQQ (nM) in Plasma and Tissues				
Parameters		PQQ Treatments^1,2^		
Experiment Designation	Tissue	PQQ+	PQQ−	PQQ−/+
Lipid Assessment	Plasma (Adult)	5.2±1.3	3.5±2.1*	17.0±4.5**
	Liver (Adult)	21.3±16.4	5.4±2.6**	26.1±17.2
	Heart (Adult)	3.4±1.9	2.4±1.0*	13.6±10.2*
Energy, Glucose, insulin, FFA Assessment	Plasma(Weanling/young)	10.1±4.7	0.71±0.35***	16.4±2.9
Glucose, insulin, FFA	Plasma (Adult)	11.3±6.7	2.6±1.35**	18.5±4.9
Ischemia Reperfusion	Plasma (Adult)	10.7±2.1	0.7±1.5**	ND
**mtDNA/NuclearDNA Ratio^3^**				
Lipid Assessment	Liver (Adult)	1.0±0.18	0.78±0.12**	1.3±0.06
Energy, Glucose, insulin, FFA Assessment	Liver(Weanling/young)	1.0±0.18	0.84±0.07	1.06±0.08
Glucose, insulin, FFA	Liver (Adult)	1.06±0.19	0.77±0.15*	1.28±0.19
	Heart (Adult)	1.0±0.21	0.85±0.17	1.1±0.2
Ischemia Reperfusion	Liver (Adult)	1.0±0.08	0.72±0.1**	1.1±0.1

1 Rats were fed an amino acid-based semi-purified diet either deficient in PQQ (PQQ−) or with PQQ added at 2 mg/kg diet (PQQ+). Rats initially fed PQQ− diets were also repleted with PQQ by i.p. injection (4.5 mg PQQ/Kg BW/24 hours X 3).

2 The superscripts _*, **, ***_ represent significance relative to the PQQ+ group at p<0.2; <0.05; or p<0.01, respectively.

3 The relative amounts of liver mitochondrial DNA (mtDNA) and nuclear DNA were measured by real-time PCR. The targeted genes were the nuclear cystic fibrosis and the mitochondrial nicotinamide adenine dinucleotide dehydrogenase-5 gene. When corresponding values for liver in each experiment are averaged, the liver values were significant at p<0.01 (PQQ+ vs PQQ− and PQQ+ vs PQQ −/+ based on ANOVA analysis using a Bonferoni correction); for heart, p<0.3.

For the studies related to energy expenditure, 4-week-old rats were used. The rats were selected from litters of dams fed either the PQQ− or PQQ+ diet throughout gestation to obtain two groups that could be matched with respect to body weighs (49±2 g). For the ischemia reperfusion study, rats (males) were 12 weeks old and weighed 375±9 grams.

Prolonged exposure to PQQ− diets consistently resulted in reduced mitochondrial contents in liver based on the decrease in the mtDNA/nuclearDNA ratios compared to rats fed PQQ+ diets (p, 0.01). For heart tissue, values for the mtDNA/nuclearDNA ratios also tended to be lower.

### Lipid Profiles

**Figure 1:**
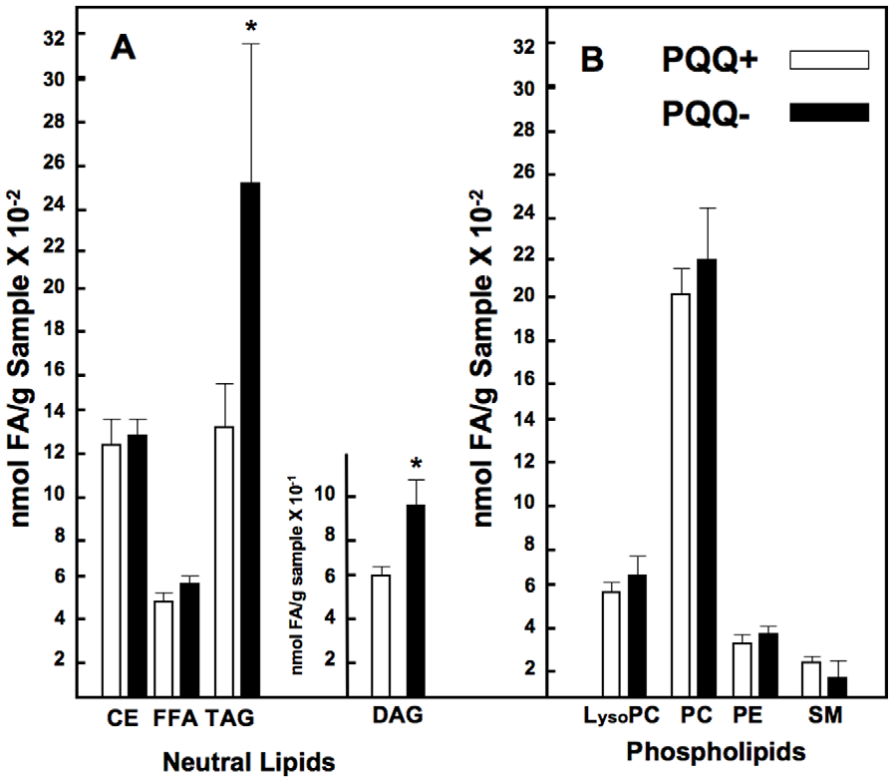
Effect of PQQ nutritional status on circulating lipid profiles in weanling rats. Values are expressed as nmol fatty acid/g plasma and are the means ± SEM (n = 5 per group). Panel A indicates changes in neutral lipids. For TAG and DAG, the asterisk indicates p<0.05. Panel B indicates changes in the major phospholipids; no differences were observed.

Distinct differences in serum lipid profiles were observed. Plasma di- and triglyceride (DAG and TAG) levels were elevated (∼20–50 %) in both young (weanling) and adult rats fed the PQQ− diets compared to rats fed PQQ+ diets (**Table 2** **, Tables S1, S2, S3, S4, S5, S6, S7, and S8, and** **Figure 1**). These results are in keeping with data reported previously for mice [21]. In **Table 2**, values labeled n3, n6, n7, n9, and DM (dimethoxy moieties derived from plasmalogens) represent families of fatty acids arising from or influenced by various desaturation pathways. Examination of individual lipids and lipid classes indicated that changes in PQQ status did not influence major desaturase or elongation systems. For example, the n6/n3 ratios, although elevated, did not differ between groups (e.g., 41±3 for neutral lipid fractions and 21±2 for phospholipid fractions **(** **Table 2** **).** The high values are in keeping with the use of corn oil as the dietary lipid source (n6/n3 ratio range: 42–46). Given that for specific fatty acids (e.g., Mead acid, 20:3n9 and arachidonic acid, 20:4n6), their plasma ratios (20:3n9/20:4n6) can be useful to assess fatty acid deficiency or defects in fatty acid desaturation pathways, attention was paid to changes in the ratio [23], [24]. For each major class of neutral lipids and their constituent fatty acids (Cholesterol esters, **Table S1**; plasma free fatty acids, **Table S2**; diacylglyceride, **Table S3;** triacylglyceride, **Table S4**) the major phospholipid classes and their constituent fatty acids (Lysophosphatidylcholine, **Table S5**; phosphatidylcholine, **Table S6**; phosphatidylethanolamine, **Table S7;** sphingomyelin, **Table S8**), most of the values were well below 0.02, whereas values for the 20:3n9/20:4n6 ratio of >0.2 suggest essential fatty acid deficiency [24]. Further, no clear differences were observed for given fatty acids in each fatty acid subclass, except for elevations corresponding to the increases in the DAG, TAG, and NEFA fractions associated with rats fed the PQQ− diet and reversal with repletion. Trace amounts of *trans* n7 fatty acids were observed, most likely arising from the corn oil used as the lipid source.

**Table 2:** Pyrroloquinoline Quinone and Plasma Lipid Composition.^1^

Lipid Class^1^	Treatment	Total Lipid^2^	Fatty Acid Composition^1^							
			SFA	MUFA	PUFA	N3	N6	N7	N9	DM
**Neutral Lipids (nmol fatty acid per g sample)** 2										
**Cholesterol Ester**	**PQQ+**	1219±268	188±21	114±22	914±233	15±3.4	899±231	16±3.9	98±18	0
	**PQQ−**	1271±234	189±27	103±19	977±195	13±3.6	963±192	14±3.6	89±16	0
	**PQQ−/+**	1439±78	199±23	**89** † **±7.1**	1149±56	17±3.0	1131±59	14±1.9	**74** † **±9.0**	0
**FFA** 1	**PQQ+**	467±83	196±44	96±16	175±25	8±3.7	166±22	12±3.4	82±12	0
	**PQQ−**	508±55	197**±41	106±8.0	203**±22	9±4.8	193** ±23	11±4.0	93** ±11	0
	**PQQ−/+**	371±122	148±40	80±30	143±52	7±2.4	136±50	11±4.6	66±25	0
**Diacylglycerol**	**PQQ+**	59±9.8	27±4.4	15±3.6	17±5.8	0.7±0.6	16±5.7	1.8±0.7	12.6±3.0	0.5±0.8
	**PQQ−**	95*±26	34**±7.0	23*±7.0	35*±12	3.8*±2.4	31*±11	2.9*±0.8	20* ±6.3	1.9±1.6
	**PQQ−/+**	60±13	21**±1.7	16±2.3	23±9.4	0.8±0.5	22±9.0	1.8±0.1	14±2.4	0.3±0.1
**Triacylglycerol**	**PQQ+**	1267±340	319±72	298±97	642±169	16±8	623±163	25±7	275±90	6±3
	**PQQ−**	2542*±1239	549**±240	624*±299	1358*±699	36**±21	1315*±672	45**±22	586* ±286	7.4±3
	**PQQ−/+**	**2177** † **±704**	**533** †† **±189**	**527** † **±172**	**1112** † **±340**	**37** †† **±23**	**1070** † **±322**	**47** †† **±25**	**483** †† **±150**	4±2
**Phospholipids (nmol fatty acid class per g sample)** 2										
**LysoPC** 1	**PQQ+**	543±58	295±32	37±5.8	208±29	4.8±1.3	204±29	8.3±1.1	28±4.9	1.5±0.9
	**PQQ−**	621*±68	323±43	40±4.4	255**±21	6.4**±0.9	249**±20	9.5±1.5	30±3.1	1.9±2.0
	**PQQ−/+**	**703** †† **±6.8**	**360** †† **±22**	**45** † **±4.5**	**295** †† **±19**	**8.7** †† **±1.9**	**287** †† **±17**	11††±1.5	35±5.4	1.4±0.5
**PC** 1	**PQQ+**	2036±329	906±148	107±15	1017±167	46±9.0	970±158	31±6.0	77±8.8	4.7±1.1
	**PQQ−**	2254±624	1011±221	122±47	1101±385	55±20	1042±373	35±16	91±27	7.0±3.7
	**PQQ−/+**	**2462** †† **±106**	**1103** †† **±50**	121±4.5	**1229** †† **±52**	**68** † **±4.0**	**1161** † **±53**	36.7±4.2	86±8.8	6.3±1.5
**PEA** 1	**PQQ+**	378±57	166±25	47±17	152±34	5.6±1.4	146±33	4.2±1.4	42±16	11.2±3.0
	**PQQ−**	395±87	165±40	57±10	162±41	7.3±1.5	154±40	4.8±1.0	51±9	10.4±1.6
	**PQQ−/+**	**464** † **±15**	**201** † **±15**	47±21	**203** †† **±19**	**9.4** †† **±2.4**	**194** † **±17**	4.6±1.8	41±19	12.8±0.8
**Sphingomyelin**	**PQQ+**	195±84	124±60	45±17	24±11	8±5	17±7	2±1	42±16	0
	**PQQ−**	165±84	101±56	42±20	21±10	7±3	15±6	1.6±1	40±19	0
	**PQQ−/+**	263±135	193±107	42±16	27±13	7±4	18±7	1±1.6	43±16	0

1 Values are mean ± SD; Abbreviations: FFA (free fatty acid or non-esterified fatty acids), LysoPC (lysophospholipid), PC (phosphocholine), PEA (N-acylphosphatidylethanolamine).

2 For comparisons: for PQQ+ vs. PQQ−, a single asterisk indicates p<0.05 and two asterisks indicate P<0.1 relative to the PQQ+ group; for PQQ+ vs. PQQ +/−, a

†indicates p<0.05 and

††indicate P<0.1 relative to the PQQ+ group.

Fewer changes (PQQ− vs. PQQ+) were observed for phospholipids, except for apparent increases in lysophosphatidylcholine, phosphatidylcholine, and phosphatidylethanolamine plasma fractions upon acute repletion of rats fed the PQQ− diet with PQQ (i.p.). In contrast, a consistent, although highly variable, reduction in sphingomyelin was observed in the majority of samples for both adult and weanling rats (**Table 2** **, Table S8, and** **Figure 1** **)**.

### Genes Important to Lipid Metabolism

Previously, we have described changes in hepatic transcription networks from which the elevations in plasma neutral lipids (**Table 2** **and** **Figure 1**) could be predicted [19]. It could be inferred that the changes in hepatic transcription networks and elevation in plasma neutral lipids was a consequence of decreases in mitochondrial amount and the expression of enzymes and transport proteins associated with β-oxidation [19], [20]. As an extension to these observations additional corroboration, changes in the levels of PPARα, peroxisomal membrane protein 4, methyl CoA racemase, and acyl CoA oxidase mRNAs levels were measured as markers as indirect measures of peroxisomal activity. Because of its relationship to β-oxidation, fatty acid binding protein mRNA levels were also determined.

**Figure 2:**
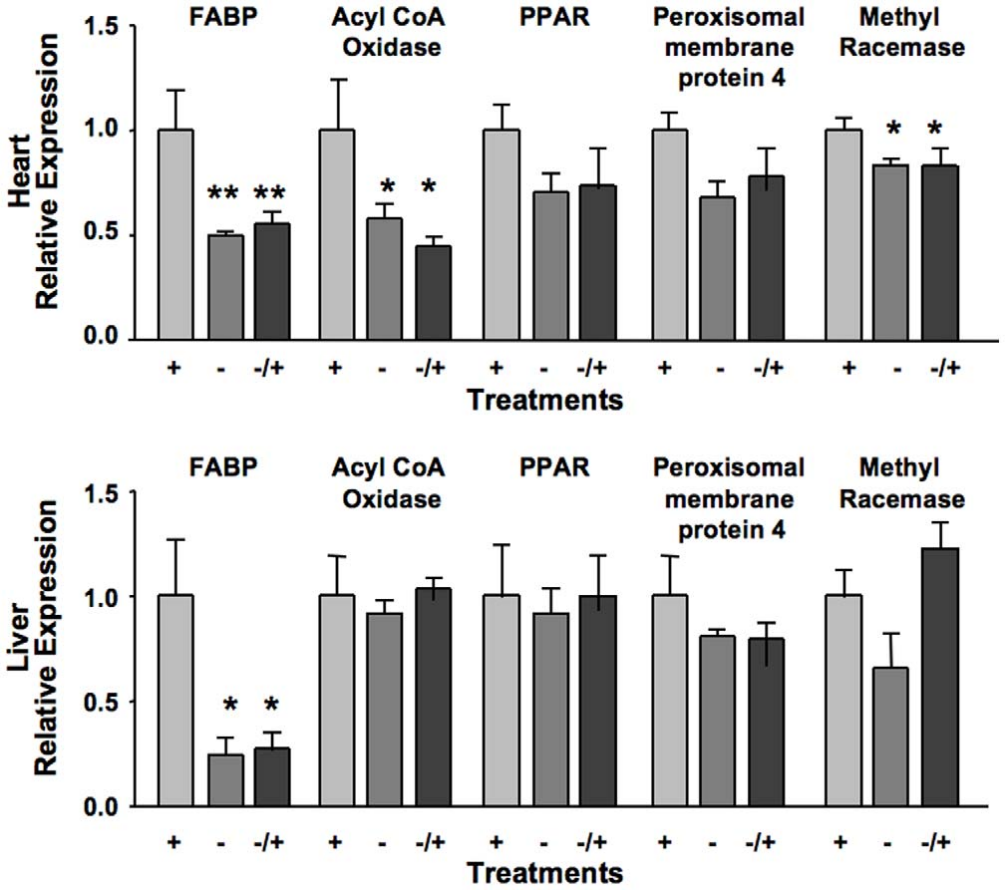
Effect of PQQ nutritional status on heart (A) and liver (B) mRNA expression levels for lipid and peroxisomal-related genes. Values are the mean ± SEM of relative expression values generated from qPCR measurements (n = 5 per group). For cardiac tissue, the expressions of FABP mRNA (**, p<0.001), acylCoA oxidase (*, p<0.01), and α-methyl CoA racemase (*, p<0.04) were statistically decreased in PQQ− rats vs. PQQ+ fed rats with trends for PPARα (p<0.2) and PMP4 (p<0.1). For liver, only FABP mRNA levels were clearly decreased (*, p<0.01) in rats fed PQQ− diets vs. PQQ+ fed rats (see discussion).

**Figure 3:**
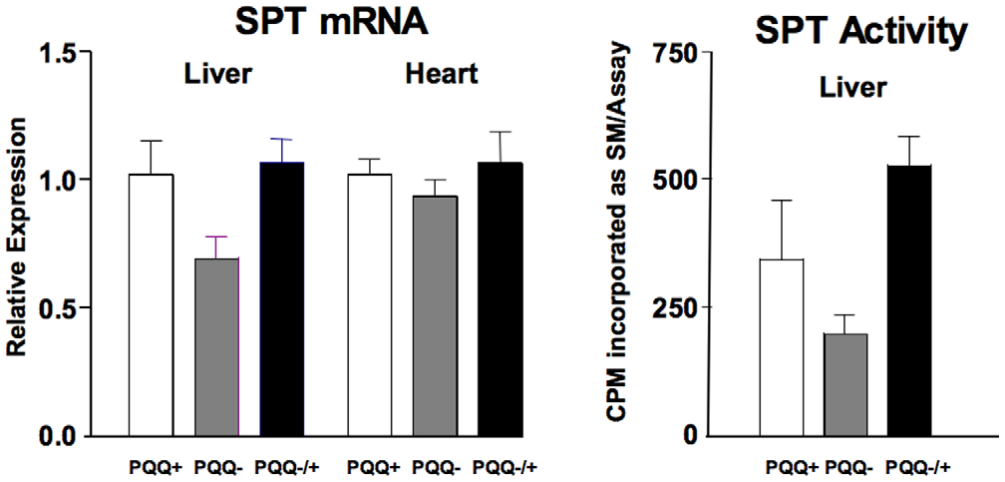
Serine palmityl transferase (SPT) mRNA expression in liver and heart and SPT functional activity levels in liver. Values are the means ± SEM (n = 5 per group). Although variable, rats fed PQQ− diets tended (p∼0.2–0.3) to have lower values for mRNA expression and activity (see Discussion).

The qRT PCR data (**Figure 2** **)** indicated that PQQ deprivation caused perturbations in the levels of mRNA for most of the markers in liver and cardiac tissue with statistically significant changes in fatty acid binding protein, acyl CoA oxidase and methyl CoA racemase in cardiac tissue, but only for fatty acid binding protein and in liver. Perhaps owing to the apparent stability of the peroxisomal markers that were chosen and the stability of fatty acid binding protein, repletion with PQQ (−/+), had little effect on immediately reversing the effects of prior PQQ deficiency (see Discussion). The mRNA levels for serine palmitoyl transferase, as well as its functional activity were also decreased (PQQ− vs. PQQ+, p∼0.1, **Figure 3**). The decreases in SPT mRNA and functional activity paralleled the variable, but consistent decreases in plasma sphingomyelin in young and adult rats (cf. **Table 2** **, Table S8, and** **Figure 1**).

### Glucose, Insulin, NEFA, and β-BHA

**Figure 4:**
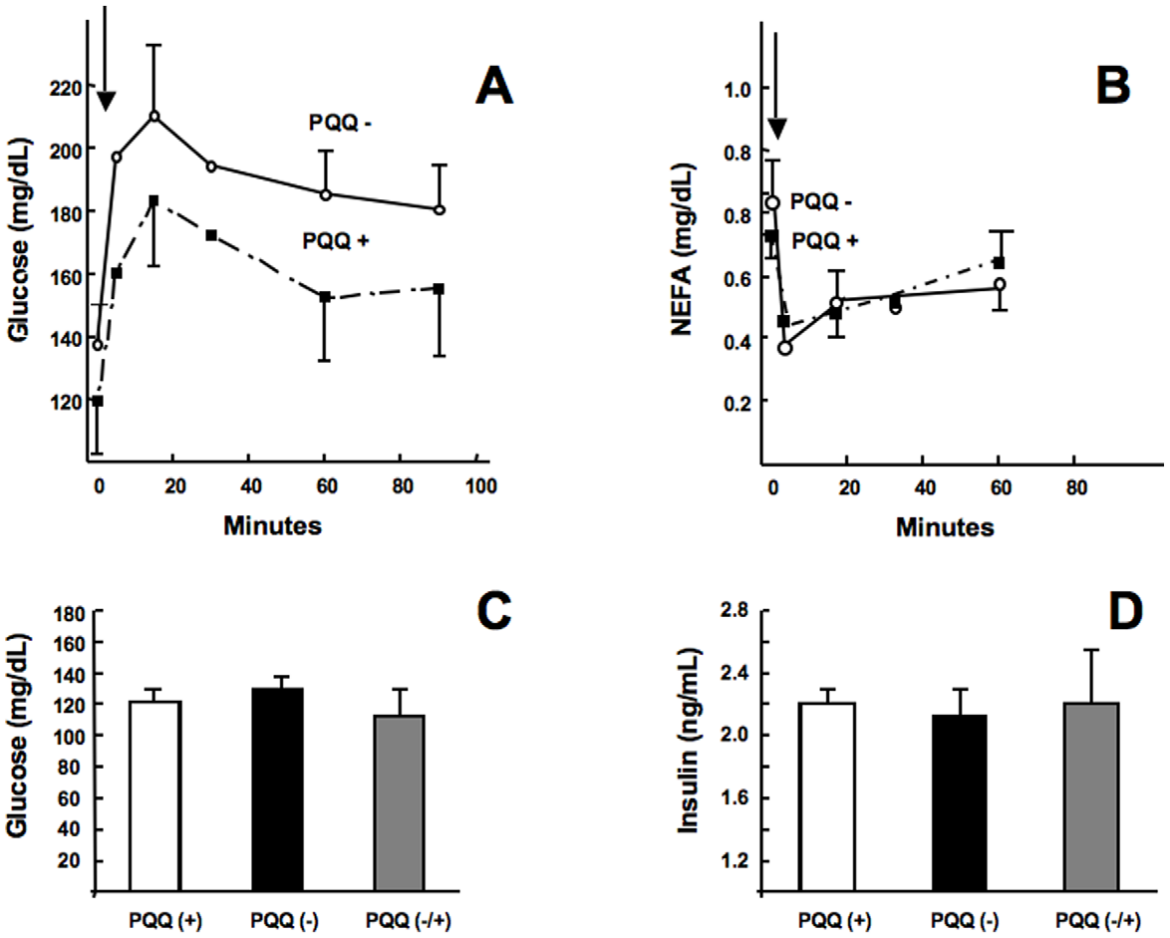
Glucose, NEFA, and insulin in rats following a glucose challenge. (A) Plasma glucose and (B) NEFA in PQQ+ and PQQ− fasted rats given 2 g glucose/kg of body weight by oral gavage. Blood was collected at 2, 5, 15, 30, 60 and 90 min intervals. Although glucose clearance appeared compromised in PQQ− rats following a glucose challenge, at each of the time points, there was no statististically significant difference compared to the PQQ+ group. Similarly, there was no difference in NEFA between groups following the glucose challenge, and no differences between PQQ+ and PQQ− rats in baseline plasma glucose (C) or insulin (D).

Several experiments were performed in which PQQ was administered interparentenially, or rats were subjected to the dietary deprivation protocol. The data in **Table 3** indicate for young Sprague-Dawley rats that fasting glucose and insulin concentrations were not significantly affected by either change in dietary status or acute PQQ administration (i.p.). However, PQQ administration (i.p.) to rats fed PQQ− diets caused a decrease in non-esterified free fatty levels (NEFA). Similar results were obtained using adult Sprague-Dawley rats (**Figure 4**). Although plasma glucose levels was slightly higher following oral glucose tolerance testing in PQQ deprived rats (**Figure 4A**), the difference (PQQ− vs. PQQ+) at each time point was not highly significant. Clear differences were also not observed in circulating plasma NEFA before or following oral glucose administration (**Figure 4B**). The administration of PQQ (i.p.) also did not alter significantly basal plasma glucose or insulin levels (**Figures 4C and D**).

**Table 3:** Effects of short term PQQ administration (i.p.) on Plasma Glucose, NEFA, and Insulin levels in Young Sprague-Dawley Rats (4 weeks old) fed PQQ− or PQQ+ Diets.^1^

Dietary State	Diet	PQQ	Time (Hours)	Glucose (mg/dL)	NEFA	Insulin
Fed	PQQ−	0 mg PQQ/kg BW	0	155±33	0.86±0.07*	103±20
Fed	PQQ−	3 mg PQQ/kg BW (PQQ−/+)	2	172±16	0.49±0.04	100±19
Fasted	PQQ+	0 mg PQQ/kg BW	0	153±19	0.53±0.06	83±21
Fasted	PQQ+	3 mg PQQ/kg BW (PQQ−/+)	2	175±15	0.50±0.06	107±22

1 PQQ was administered at 4.5 mg PQQ/kg BW (n = 4 per group) followed by assessment of changes in plasma glucose, NEFA, and insulin. An asterisk (*) indicates p<0.05 relative to the PQQ+ or PQQ−/+ group in the fed or fasted state.

**Figure 5:**
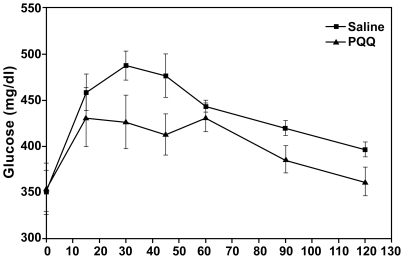
Oral glucose tolerance in response to a glucose load in diabetic UCD-T2DM Rats following the administration of PQQ (i.p.) at 4.5 mg PQQ/Kg BW for 3 days or saline. The area under the respective curves was reduced by 7 percent when rats given saline were compared to rats administered PQQ (p∼0.09).

As an additional validation of these findings, the availability of the UC Davis type-2 diabetic model rat (UCD-T2DM) [22] allowed the testing of whether PQQ can influence glucose tolerance in animals with existing diabetes. The data in **Figure 5** indicate that modest changes in oral glucose tolerance may occur in response to PQQ administration. The area under the respective curves was reduced by 7 percent when rats given saline was compared to rats administered PQQ for 3 days at 4.5 mg/ kg BW (p∼0.09). PQQ was also administered to UCD-T2DM rats, which were then subjected to a 6 h fast. No differences were observed (PQQ vs. saline) in plasma glucose or NEFA concentrations over the test period. For example, at 0, 3, and 6 h, the values for plasma free fatty acids were 0.43±0.02, 0.76±0.08, and 0.6±0.06 mg/dL for rats administered saline; in contrast, to 0.39±0.02, 0.69±0.07 and 0.58±0.06 mg/dL for rats administered PQQ. With regard to plasma triacylglyceride levels, values for control rats fell from 310 mg/dL ±49 to 236 mg/dL ±37 (i.e., Δ 74 mg triglyceride/dL) over the period of the 6 h fast following saline administration. In contrast, PQQ administration resulted in a greater than a 2-fold decrease to 141 mg/dL ±28 (p<0.05) or a Δ169 mg change in triglyceride/dL.

**Figure 6:**
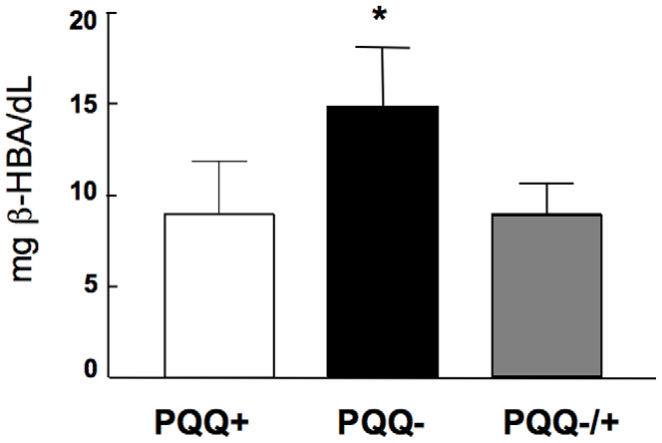
β-hydroxybutryic acid levels in rats fed the PQQ− or PQQ+ diets. The increase in β -hydroxybutryic acid was reversed upon PQQ repletion (p<0.05).

That PQQ administration caused changes in lipid clearance is also reflected by a significant increase in β-HBA levels in rats fed the PQQ− diet, which were reversed upon PQQ repletion (**Figure 6**). These data were taken as an additional indirect or functional measure of decreased β-oxidation potential due to the reduction in mitochondrial amount.

### Energy Expenditure

**Figure 7:**
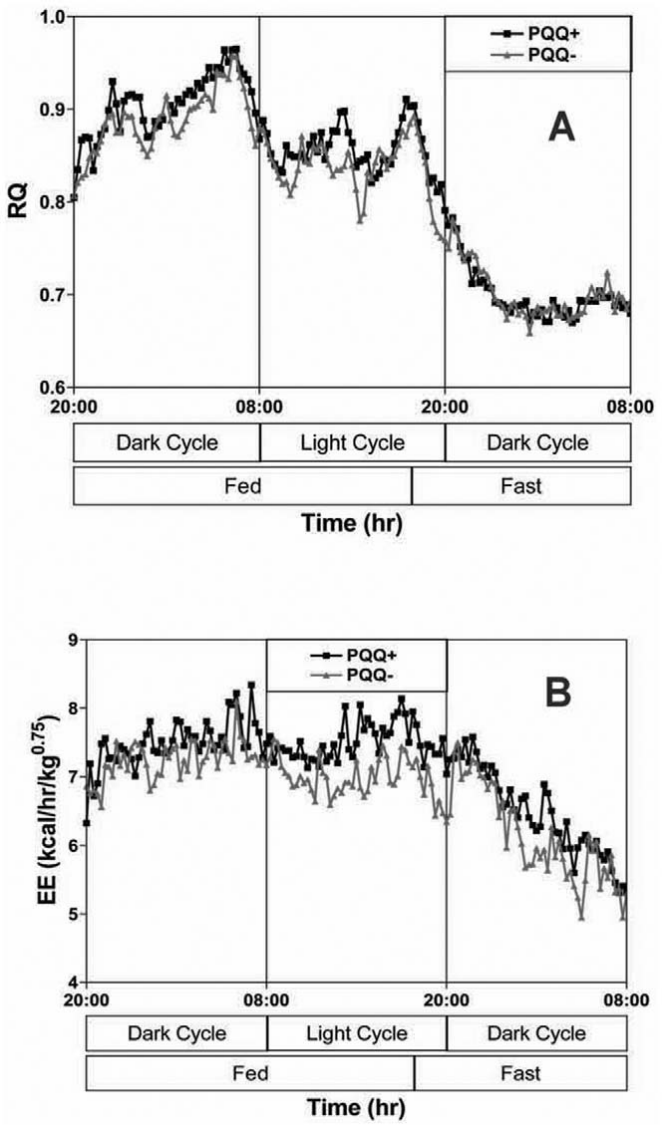
Changes in RQ and energy expenditure (oxygen consumption) in weaning rats derived from dams fed PQQ− and PQQ+ diets. No differences in apparent meal frequency or RQ over the 24-h period of observation. The aggregate energy expenditure in the fed state (n = 10 for each group) was reduced in rats fed PQQ- diets (p<0.05) compared to PQQ+ rats.

**Figure 8:**
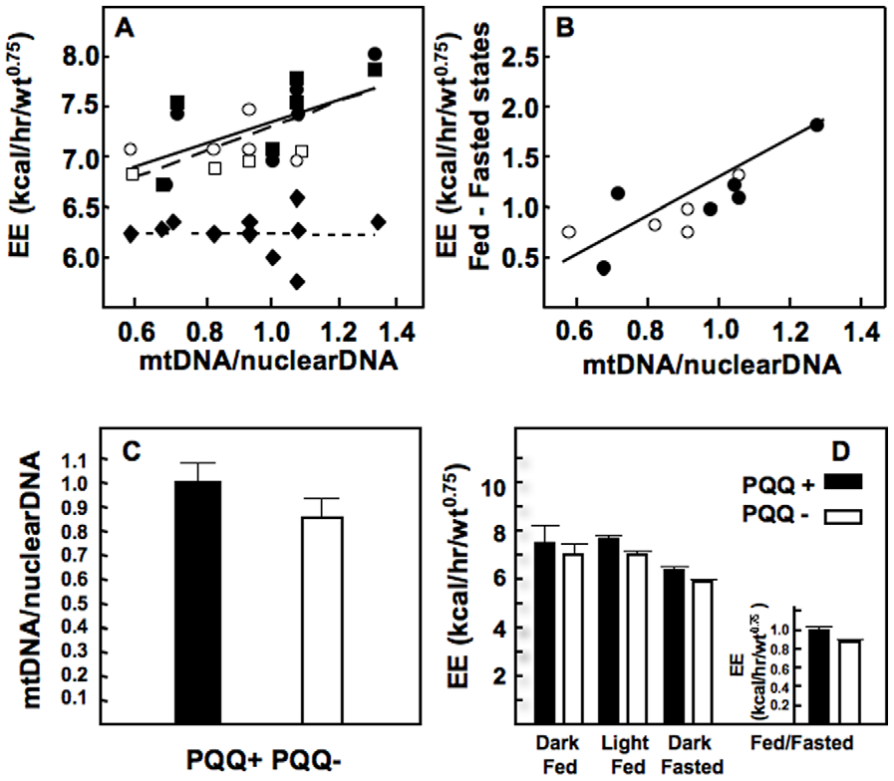
Relationship between relative mitochondrial amount (liver) and energy expenditure. In panel **A** the relationship between mitochondrial amount (mtDNA/nuclearDNA ratio) and energy expenditure in the light fed (squares) and dark fed (circles) for weanling rats fed PQQ- (open, n = 6) or PQQ+ (closed, n = 5) diets are shown; R∼0.6. Little or no relationship was observed when rats were fasted (⋄,PQQ-;⧫, PQQ+). In panel **B**, the average energy expenditure in both fed states minus the fasted state are compared. (Light and dark fed states) - (fasted state) are expressed relative to the mtDNA/nuclearDNA ratio. R was increased to ∼0.9. In panel **C** are data for the relative mitochondrial amount (mtDNA/nuclearDNA ratio). The average difference mitochondrial amount (∼10 %) was not significant for rats that were randomly selected out of a pool of 10 for each group (**see** **Figure 6**). In panel **D** are values for the fed and fasted states. The average decrease in total energy expenditure (PQQ- vs PQQ+) approached significance (p<0.1), when values for the fasted state (taken as a rough approximation of basal energy) were subtracted from the values for the two fed states (light and dark).

Data related to the effects of changing PQQ status on RQ, VO_2_, VCO_2_, and estimated energy expenditure are given in **Figures 7** and **8**. Although there was little change in RQ levels with respect to treatments, there was a significant difference in actual energy expenditure, which could be positively correlated with mitochondrial amount during periods when the rats were most active (see Discussion).

### PQQ and Cardiac Muscle Function and Protection: Ischemia Reperfusion

Previously it was reported that PQQ is protective when rats were subjected to 17 or 30 min of left anterior descending occlusion when reperfused with PQQ (15–20 mg/kg BW) or given PQQ (i.p.) either 30 min before occlusion or i.v. at the onset of reperfusion [10], [11]. With regard to dietary exposure, the data suggest that PQQ dietary exposure may be protective. Four out of 17 rats fed the PQQ- diet did not survive the ischemia reperfusion protocol, whereas all of the PQQ+ fed rats survived (**Table 4**).

**Table 4:** Dietary PQQ and Protection from an Ischemia/Reperfusion Injury.

Ischemia Protocol and Experimental Groups^1^				
Parameter	Ischemia/Reperfusion (I/R)		Preconditioning plus Ischemia/Reperfusion (P/I/R)	
	PQQ+ (n = 8)	PQQ- (n = 8)†	PQQ+ (n = 7)	PQQ- (n = 9)
Body Weight (g)	377±8.7	370±9.9	387±8.3	386±5.6
Heart weight (g)	1.12±0.07	1.20±0.05	1.16±0.07	1.21±0.04
LV weight (g)	0.87±0.04	0.90±0.03	0.87±0.04	0.93±0.02
Heart/BW (%)	0.30±0.01	0.32±0.01	0.30±0.02	0.31±0.01
Mortality (%)^1^	0	37.5 (3/8)^1^	0	11.1 (1/9)^1^
Infarct/LV (%)	24.2±1.7	23.7±2.3	16.8±2.9^2^	17.7±1.8^2^
CK Base-line (U/L)	794±167	537±114	480±147	1026±355
CK 2 h reperfusion (U/L)	2340±258^3^	1842±280^3^	1987±360^3^	2397±400^3^
CK (Fold increase)	3.73±0.35	4.23±0.89	5.12±1.0	4.34±1.4
RCR-ischemic	2.85±0.46	2.57±0.47	4.17±0.62^4^	3.64±0.33^4^
RCR-non-ischemic	4.94±0.28	4.35±0.46^4^	5.17±0.35	4.43±0.37^4^
MDA-ischemic	508±76	359±73 ↓	375±66	344±84 ↓
MDA-non-ischemic	194±19^5^	247±45 ↑^5^	202±43^5^	331±69 ↑^5^

1 Four rats in the PQQ- group died during either I/R or P/I/R protocols compared to none in the PQQ+ group (p∼0.1, chi square analysis). In the I/R-PQQ- group, one rat died of acute pulmonary edema before surgery, one after opening the chest, and one late during reperfusion. The latter heart was salvaged and is included for the estimates of infarct/LV (left ventricular)%, CK (creatine kinase) levels, RCR (mitochondrial respiratory control ratio) and MDA (malondialdehyde) levels. Two rats in the PQQ+ group died before suture of LAD and were excluded.

2 The P/I/R protocol reduced LV infarct size ∼30 percent (I/R vs. P/I/R, P<0.005).

3 CK levels were increased ∼4-5+ fold following I/R (p<0.01). The increase was refractory to PQQ exposure.

4 The RCR in ischemic areas of the LV was improved with preconditioning (p∼0.05). In non-ischemic areas of the LV, the RCR tended to be higher in response to PQQ exposure (p∼0.1).

5 MDA levels were lower in non-ischemic areas vs. ischemic areas (p<0.05). In non-ischemic ares, PQQ exposure resulted in a 30-40 % reduction in MDA levels (p<0.05).

When a preconditioning protocol was introduced prior to ischemia reperfusion protocol, left ventricular infarct size was reduced ∼30 % in both PQQ- and PQQ+ rats (P<0.005 by 2-way ANOVA). In addition to defined areas of ischemia, creatine kinase was increased 4 to 5- fold. Although as indices, the amount of tissue observed to be ischemic and the creatine kinase increase were refractory to PQQ exposure, the values for RCR tended to be higher in response to PQQ exposure (p∼0.1). Further, malondialdehyde levels were lower in non-ischemic areas vs. ischemic areas (p<0.05), and in non-ischemic areas, PQQ exposure resulted in a 30-40 % reduction in MDA levels (p<0.05).

## Discussion

We have previously reported that liver from rats fed a diet deficient in PQQ have fewer mitochondria in liver tissue based on mtDNA measurements [16]-[21], [25]. In this regard, PQQ appears to be among a growing number of compounds that seem to improve mitochondriogenesis and/or function in experimental animal models. As examples, the nutraceutical use of quercetin [26], resveratrol [27], [28], hydroxytyrosol [29], flavanols, such as epicatechin gallate [30], and combinations of nutrients, such as R-α-lipoic acid, biotin, acetyl-L-carnitine, and nicotinamide have been reported to enhance abnormal mitochondriogenesis or improve mitochondrial function [31]. In addition, such nutritional treatments often influence the mRNA levels of genes involved in lipid metabolism, including peroxisome proliferator–activated receptor-α, carnitine palmitoyl transferase-1 and the activities of mitochondrial complex I and II [32], although many of the mechanistic details are lacking. However, a novel feature for PQQ is that the mitochondrial-related perturbations occur at relatively low levels of dietary exposure in rodents (<250–300 nmol/1000 kCal or 4.2 MJ) and pharmacologic doses administered in the low mg/kg body weight range [17], [18]. In contrast, many other biofactors are needed in the sub to high µM ranges when expressed per 1000 kCal or 4.2 MJ of typical laboratory diet mixtures [32]. For example, resveratrol can enhance mitochondriogenesis, but in animal models, 200–500 mg resveratrol/kg diet are usually used for a response [33]. From a food perspective, this amounts to more than 100 times that usually found in a typical bottle of red wine [34]. A minimum 0.2–0.3 mg PQQ per kg of diet seems to support normal growth and development in mice, which is about 1/2 the requirement for folic acid in mice (e.g., 0.4 to 0.5 mg folic acid/kg of diet) and close to the concentration of pyrroloquinoline quinone and PQQ-amino acid derived products found in human milk solids [2].

For the studies described herein, we chose higher concentration of PQQ than we have previously used (e.g., 2 or 10 mg PQQ/kg of diet or 4.5 mg PQQ administered i.p./kg body weight) to better assure optimal tissue saturation. These amounts (excluding PQQ-amino acid derived adducts, such as imidizolopyrroloquinoline) are about 10–50 times those found in human diets based on what food compositional data are currently available [1], [2].

The apparent reduction in the amount of liver mitochondria in groups of mice [18], [21] and rats deprived of PQQ (with recovery upon PQQ repletion) has been a consistent finding [16], [19]. A statistical difference was not observed in adult rat cardiac muscle (PQQ- vs. PQQ+, **Table 1**); however, the pattern was similar to that for liver and supported by previous functional data in vitro that indicate PQQ exposure protects cardiac mitochondria [10], [11], [13]. Given that most animal models of mitochondrial dysfunction result in elevated TG or perturbations in lipid metabolism [35], we infer that the reduction in mitochondrial amount and presumably function in PQQ deprived rats is a principal underlying cause for the changes observed in plasma lipids. In addition, the reduction in FABP and CPT mRNA levels and their respective products are rate-limiting steps in the delivery of long chain fatty acids to the mitochondria; thus, may be among additional factors [36]. Although many details regarding mechanisms remain to be resolved, that PQQ exposure activates CREP phosphorylation and increases in the expression of PGC-1α, transcriptions factors NRF1 and 2, and TFAM (transcription factor A, mitochondrial) are all consistent with induction of mitochondriogenesis and perturbations in circulating lipids fractions, because of concomitant alterations in β-oxidation [37].

Regarding the modulation of plasma glucose and insulin by PQQ administration, although the changes were modest, trends in the data did not exclude the possibility that at higher concentrations PQQ may be effective. Milne et al. [38] have described novel activators of SIRT1, whose administration to insulin-resistant animals improves glucose homeostasis. The molecules, various resveratrol analogs, caused SIRT1 activation, which has been linked to increased mitochondriogenesis and attenuating insulin resistance in animal models, such as the Zucker *fa/fa* rats and genetically obese mice (*Lep^ob/ob^*). The doses of resveratrol or the analogs ranged from 100–1000 mg per kg body weight, i.e. ∼20-200 the amounts of PQQ that were given intraperitoneally. Accordingly, higher doses of PQQ might be considered in future studies. Moreover, we previously identified the insulin receptor and carbohydrate metabolism as possible targets for PQQ using Ingenuity pathways analysis software (http://www. ingenuity.com/index.html [19]). The Ingenuity based analysis indicated that PQQ repletion of PQQ deprived rats, using a protocol similar to the one described herein, caused a 2-fold increase in several of the insulin-induced family of genes (e.g., insulin induced gene 2) and a 2-fold decrease in insulin degrading enzyme.

We also found it of interest that in addition to modest increases in plasma lysophosphatidylcholine, phosphatidylcholine, and phosphatidylethanolamine levels upon acute repletion of rats with PQQ (i.p.) fed the PQQ- diet), there were reductions in plasma sphingomyelin levels in PQQ-deprived adult and weanling rats. The changes in plasma phospholipids were assumed related or linked to cellular membrane changes associated with tissue remodeling. Although not statistically significant, we focused on changes in sphingomyelin levels, because of corresponding decreases in serine palmityl transferase based on measurement of both functional activity and SPT mRNA levels and previous gene array analysis [19]. Sphingolipids also facilitate formation of more mechanically and chemically stable plasma membrane lipid bilayers [39], [40]. Moreover, ceramides and sphingosine (as sphingosine-1-phosphate), whose formation is dependent on serine palmitoyl transferase, are also involved in cell signaling, apoptosis, and cardioprotection [41]–[43]. Regarding other fatty acid modifications, there were no consistent changes in the concentrations of PUFAs with chain lengths of >20 carbons nor in the in the n6/n3 ratio, or concentration of Mead acid (20:39(n-9), (5Z, 8Z, 11Z)-Eicosa-5, 8, 11-trienoic acid) in the major lipid fractions. C_20_-C_22_ PUFAs were examined because they often function in key cell signaling steps and govern the expression of a wide array of genes, for example, down-regulation of hepatic lipogenic genes while up-regulating genes associated with fatty acid oxidation [44]–[46].

We also examined the expression of five genes selected to ascertain the extent to which PPAR activation and cellular fatty acid transport may contribute to the disturbances in lipid metabolism in PQQ- rats. Liver and cardiac fatty acid-binding proteins (FABP) are abundant proteins that bind most of the long chain fatty acids present in the cytosol. Their cytosolic levels provide an additional control on β-oxidation [47]–[50]. Liver and cardiac FABP mRNA levels (FABP-1) were reduced 50% or more in PQQ- deficient rats. Although changes in FABP mRNA levels were refractory to PQQ repletion, rat hepatic fatty acid binding protein is relatively long-lived (∼3 days). For example, Bass et al [50] have reported that following a 48-h fast, total liver FABP decreases 65% and re-feeding for 24 h does not lead to a significant recovery of liver FABP.

As markers for potential peroxisomal regulation, acyl-coenzyme A oxidase 1, PPARα, peroxisomal membrane protein 4, and α-methyl CoA racemase were examined. It was expected that if PQQ functions through cAMP-responsive transcription factor regulated pathways, each of the selected peroxisomal components would be influenced to some degree in keeping with the changes in mitochondrial components. Although some of the changes were not significant, all of the genes used as markers were reduced from 10-45 % in PQQ- rats and most were increased or normalized upon PQQ repletion. With regard to specific changes, it is noteworthy that the changes liver PPARα were modest. Likewise using Hepa1–6 cells in culture, although PQQ exposure causes activation of the PGC-1α and related genes important to mitochondrial activation, little effect on PPARα was observed (**Figure S1**). As a consequence, we infer that the effects of PQQ may be more related to stimulation of mitochondrial signaling, and perhaps FABP expression, than acting as a specific PPAR agonist.

Importantly, a relationship between the amount of mtDNA/nuclearDNA in liver and energy expenditure (estimated from oxygen consumption) was also observed. It has previously been reported that changes in oxidative capacity as little as 20 percent can have a direct influence on the sensitivity of cytosolic respiratory control and has important consequences in the maintenance of cellular energy balance [51]–[53]. Our results are keeping with such observations and help to link PQQ and non-fasting energy expenditure to the mitochondrial amount. Although RQ values were similar between groups, rats deprived of PQQ had lower estimates of VCO_2_ and VO_2_. As a final point, pharmacologic doses of PQQ (∼3 mg/kg body weight or greater) are cardioprotective in models of ischemia reperfusion injury [10], [11], [13]. The observation of 30–40 percent mortality in rats nutritionally deprived of PQQ in response to ischemia adds an interesting dimension to the cardioprotective effects of PQQ. Tao et al. [13] have also proposed that such cardioprotection may be result of increased antioxidant defense as a result of PQQ exposure. The work summarized herein adds as an additional consideration, i.e. increased mitochondrial oxidative efficiency in part related to increased mitochondriogenesis. We interpret the pattern observed for changes in MDA levels in heart sections as a reflection of differences in tissue viability. The MDA values were 1.5 to 2.5 fold higher in ischemic sections. For both tissue conditions (ischemia vs. non-ischemia), relatively lower values for RCR were accompanied by higher MDA values. Compared to non-ischemic sections of heart, it is predictable that low values for the rate of respiratory control and ischemia would lead to augmentation of lipid peroxidation.

In summary, rats fed a diet deficient in PQQ are metabolically challenged, due to decreased mitochondria number. In addition to previously reported perturbed amino acid metabolism, PQQ deficient rats also have defects in lipid and energy metabolism and are vulnerable to ischemic insult. Taken together, PQQ is a novel metabolic modulator involved in many aspects of mitochondriogenesis, mitochondrial metabolic function as well as being a cardioprotectant.

## Materials and Methods

### Reagents, Animal Care, and Nutritional Protocols

Chemicals and reagents used in diets and assays were obtained from Fisher Chemicals and Sigma–Aldrich and were of the highest purity available. Amino acids for diet preparations were purchased from Ajinomoto Co., Inc. Reverse transcription and PCR enzymes and reagents were obtained from Applied Biosystems or BioRad.

Animal Research Services at the University of California, Davis (Animal welfare assurance number, A-3433-02) and the VA Medical Center, UC San Francisco (Animal welfare assurance number, A-3476-01), approved the animal protocols for the nutritional studies (protocol 12998, RBR, UCD) and surgery related to ischemia reperfusion (protocol 03-051-01, JSK, VAMC UCSF). The American Association for the Accreditation of Laboratory Animal Care has accredited both facilities. All aspects of the work were conducted in keeping with established guidelines (*Guide for the Care and Use of Laboratory Animals*, NIH Publication No. 85-23, revised 1996). In addition, the work conducted at UC Davis (animal husbandry) followed USDA guidelines (Registration number, 93-R-0433).

Male Sprague-Dawley rats (n = 4–10 for given groups) were obtained commercially (Charles River, Wilmington, MA) and maintained in polycarbonate cages with Carefresh Total 1 Clean Bedding, (International Absorbents Corp, Bellingham, WA) in a temperature-controlled facility with a 12-h dark/light cycle and allowed to consume deionized water and purified amino acid based diet with or without PQQ *ad libitum*. Separate experiments focused on PQQ and indices important to lipid metabolism assessment and changes in glucose. In some the experiments, the effects of PQQ administration on glucose regulation were also examined in rats that were developed as a model of type-2 diabetes, the UCD-T2DM rat [22].

Rats were fed an amino acid based diet devoid in PQQ that provided all known required nutrients in sufficient quantities to provide maximal growth. The composition of the basal diet was identical to that reported by Steinberg et al [17], [18] and Stites et al [21]; the basal PQQ concentration ranged between 5–20 fmol/g diet. PQQ was added to diets at 0 (PQQ−) or 2 mg/kg (∼6 nmol/g) (PQQ+). Rats were fed diets for at least 4 weeks prior to selection for given sets of assays (see Figure and Table legends). For some experiments, a subset of PQQ- rats was repleted with PQQ (intraperitoneal, i.p., injections of 4.5 mg/kg PQQ per day (PQQ−/+) for 3 days. Unless indicated in Figure or Table legends, rats were usually fasted for 4 hrs and anesthetized by CO_2_ inhalation prior to blood and tissue collections. Blood was collected by cardiac puncture into heparinized syringes. Plasma was separated by centrifugation at 2,000 x *g* for 15 min at 4°C and stored at −20°C until analyzed.

### PQQ Estimation

PQQ was extracted from tissue and plasma as previously described [17], [18]. A glucose dehydrogenase (GDH)–based assay system was used for PQQ quantization [21], [54]. Recoveries based on samples (plasma and liver extracts) spiked with PQQ were good (e.g. >85%).

### Plasma Glucose, Insulin, and Free Fatty Acid Levels

To assess oral glucose tolerance, Sprague-Dawley rats were fed either the PQQ- or PQQ+ diet for 4 (young rats) or 8 weeks (adult rats). The rats (4/group) were examined in the fed or fasted state (overnight, 8–10 h). Blood was collected from the tail vein. After a baseline collection, 2 g glucose/kg of body weight (BW) was given by oral gavage and blood was collected at 2, 5, 15, 30, 60 and 90 m intervals to assess glucose tolerance. Plasma glucose was measured using a glucose analyzer (Analox GM7 Microstat, London, UK). In some experiments, insulin was also measured using a rat insulin RIA kit (Millipore, St. Charles MO). Non-esterified fatty acid levels were measured using a NEFA kit (Wako, Richmond, VA). The assays kits that were used each gave excellent precision and recovery without the need for extraction.

The short-term effects of PQQ administration on glucose regulation were examined in both Sprague-Dawley rats and rats that were developed as a model of type-2 diabetes, the UCD-T2DM rat [22]. The model was created by crossing obese Sprague-Dawley rats with polygenic adult onset obesity and insulin resistance with Zucker diabetic fatty (ZDF)-lean rats that have a defect in pancreatic β-cell function, but normal leptin receptor function. These rats (n = 8 per group) were fed either the PQQ+ or PQQ- diet. PQQ was also administered (i.p.) at 4.5 mg PQQ/kg BW to one-half of the rats in each group, which was immediately followed by assessment of glucose tolerance or changes in plasma glucose, triacylglycerides and fatty acid levels over a 2. Values for plasma glucose were ∼350 mg/dL for the UCD-T2DM and ∼150 mg/dL for Sprague-Dawley rats, respectively.

### Lipid Profiles and Beta-Hydroxybutryic Acid (β-BHA)

A quantitative profile of plasma lipid metabolites was generated from PQQ- and PQQ+ rats (5/group) (Lipomics Technologies Inc, now Tethys Bioscience (cf. http://www.lipomics.com/). The lipids were extracted in the presence of authentic internal standards by the Folch method (chloroform/methanol (2:1 v/v). Individual lipid classes were separated by liquid chromatography. Each lipid class was next transesterified in 1% (v/v) sulfuric acid in methanol under a nitrogen atmosphere at 100°C for 45 m. The resulting fatty acid methyl esters were extracted from the mixture with hexane containing 0.05% butylated hydroxytoluene and prepared for gas chromatography under nitrogen. Fatty acid methyl esters were separated and quantified by capillary gas chromatography (Agilent Technologies model 6890) equipped with a 30 m DB-88MS capillary column (Agilent Technologies) and a flame-ionization detector.

In addition, as an indirect marker of β-oxidation, serum β-HBA levels were measured using a commercial assay kit obtained from Catachem, Inc. (Bridgeport, CT). The assay is based on the conversion of *D*-3-hydroxybutyrate by *D*-3-hydroxybutyrate dehydrogenase to acetoacetate as described by Bergmeyer [55].

### Metabolic Data and Body Composition

The respiratory quotient (RQ), VO_2_, VCO_2_ and energy expenditure (EE) for individual rats were measured using PQQ- and PQQ+ rats (n = 8 per group). Metabolic monitoring was carried out with energy expenditure analysis system (AccuScan Instruments, Columbus, Ohio, United States) located in the UC Davis Department of Nutrition animal facility. The system consists of an O_2_ analyzer, CO_2_ analyzer, and PhysioScan analyzer that can monitor vertical and horizontal movement via light beam interruption. A flow controller/channelyzer allows for flow rate adjustments and sequential channeling of airflow from a reference line and the four animal chambers through the CO_2_ and O_2_ analyzers. The flow rate for these experiments was 0.5 l/m. The dimensions of the plexiglas chambers are 29 cm (L) X 19 cm (H) X 13 cm (W).

The Integra ME software includes O_2_ and CO_2_ analyzer calibration, data collection, and analysis programs. Reported and calculated values include O_2_ consumption, CO_2_ production, RQ, heat production (energy expenditure), total ambulatory movement, and total rest time. Animals were acclimated to the chambers for 4–6 h prior to a 24-h data collection period. Each animal received its assigned diet and water while in the chamber. Measurements were made over a 36 hrs in both the fed/dark, fed/light, and fasted/dark states (∼12 hr ea).

Body fat mass and lean mass were determined following whole body fat extraction as described by Bell et al [56].

### Mitochondrial DNA, mRNA Expression and qRT-PCR, and Serine Palmitoyl Transferase Activity

The relative amounts of liver mitochondria in rats were determined using quantitative real time PCR (qRT-PCR) methods as previously described [16], [19]–[21]. The primers for the targeted genes are given in **Table 5**. The nuclear cystic fibrosis (CF) gene and mitochondrial nicotinamide adenine dinucleotide dehydrogenase-5 (ND-5) gene were used to assess the relative mitochondrial copy number to nuclear copy number [16], [57].

**Table 5:** PCR Primers.

Gene	Forward	Reverse
Cystic Fibrosis gene, Nuclear (CF)	AAACTCAGGATAGCTGTCCGTTTAG	GCCAAATGATAGCATGGAACTCT
NAD dehydrogenase-5 (ND-5)	GGATGATGATATGGCCTTGCA	CGACTCGGTTGTAGAGGATTGC
Liver Fatty Acid Binding Protein (FABP1)	TGTAGCCCATACTGGCCTCAA	TTCAGGTTTTCCAGCATTCATG
PPAR α	TGGAGTCCACGCATGTGAAG	CGCCAGCTTTAGCCGAATAG
Peroxisomal membrane protein 4 (Pmp4)	CAGCTAAGGATTCCAGATGCTCTT	AGGCCCAGAGAGGGTTGAA
Acyl CoA oxidase (Acox1)	TGCTGGCATCGAAGAATGTC	AATCCCACTGCTGTGAGAATAGC
Carnitine Palmityl Transferase (CPT)	TGGGAGCGACTCTTCAATACTTC	TTGATATGTTGGATGGTGTCTGTCT
Methyl CoA racemase	GACCCCAGTGCTGACTCTTGA	TGAAGGAGCCCCGTTCTCT
Serine palmityl transferase (SPT)	AATGCGCTCGCTTCTGTTG	GCCAGAGAGCCGCTGATG
Actin	GGCGCTTTTGACTCAGGATT	GGGATGTTTGCTCCAACCAA

PCR was performed using an ABI 7900HT real time thermocycler (Perkin Elmer) coupled with SYBR Green technology (Applied Biosystems) and the following cycling parameters: stage 1, 50°C for 2 m; stage 2, 95°C for 10 m; stage 3, 40 cycles for 95°C for 15 s; 60°C for 1 m; and stage 4, 95°C for 15 s. The linearity of the dissociation curve was analyzed using the ABI 7900HT software. Each sample was analyzed in duplicate. The mean cycle time of the linear part of the curve was designated *Ct*. Relative mitochondrial copy number to nuclear copy number was assessed by a comparative *Ct* method (*ΔCt* mitochondria*/*nuclear  =  *Ct* mitochondria*_Ct_ − Ct* nuclear*_Ct_*) to assess for the fold-change for mtDNA/nuclear DNA from PQQ−, PQQ+, PQQ−/+ and PQQ+/− rats.

For qRT- PCR assays, RNA was extracted with Trizol (Invitrogen) and cDNAs were generated from 1 µg total RNA (Reverse Transcription Kit, Applied Biosystems, Foster City, CA) following the manufacturer's instructions. When needed, gene specific primers were selected (**Table 5**) using Primer Express® Software (Applied Biosystems).

Serine palmitoyl transferase (palmitoyl-CoA:L-serine C-palmitoyl transferase (decar-boxylating), EC 2.3.1.50) was measured based on the incorporation of ^3^H-serine into the chloroform-soluble product 3-ketosphinganine in microsomal subfractions as described by Williams et al [58].

### Ischemia Reperfusion and Infarct Size

The effects of PQQ on cardiac function were tested using an ischemia reperfusion model [10], [11]. Rats were anesthetized using pentobarbital (50 mg/kg body weight) by intraperitoneal injection. A tracheotomy was then performed and ventilation was maintained using a Harvard Rodent Respirator (Model 683, Harvard Apparatus, Holliston, Mass) as described previously [10], [11]. The level of consciousness of each animal was monitored by lack of response to foot pad stimulation. Next, a reversible coronary artery snare occluder was placed around the proximal left anterior descending (LAD) coronary artery through a midline sternotomy. Rats then underwent 30 m of LAD coronary artery occlusion and 120 m of reflow. For ischemic preconditioning studies, rats underwent 20 minutes of equilibration followed by 3 minutes of LAD coronary occlusion. This was followed by 7 minutes of reflow. Hearts were then subjected to 30 minutes of LAD coronary artery occlusion and 120 minutes of reperfusion.

Infarct size was measured as described previously. After 120 m of reperfusion, the LAD was re-occluded, and phthalocyanine dye (Engelhard Corp, Louisville, KY) was injected into the LV cavity, allowing normally perfused myocardium to stain blue. The heart was then excised, rinsed of excess dye, and sliced transversely from apex to base into 2-mm-thick sections. The sections were incubated in triphenyltetrazolium chloride (TTC, Sigma) as described above. Infarcted myocardium fails to stain with TTC. The tissue sections were then fixed in a10% formalin solution (Sigma/Aldrich) and weighed. Color digital images of both sides of each transverse slice were obtained using a video camera (DC 300 F, Leica Microsystems, Wetzlar, Germany) connected to a microscope (Stereo Zoom 6 Photo, Leica). The regions showing blue-stained (non-ischemic), red-stained (ischemic but non-infarcted), and unstained (infarcted) tissue were sectioned. Each colored area was outlined and measured using NIH Image 1.59 software (National Institutes of Health, Bethesda, MD) in a blinded fashion.

On each side, the fraction of the LV area representing infarct-related tissue (average of 2 images) was multiplied by the weight of that section to determine the absolute weight of infarct-related tissue. The infarct size for each heart was expressed as:

*Percentage Infarct size/LV mass  =  Infarct weight in each slice/Total LV weight X ∼100%*

*Risk area/LV mass (%)  =  Total weight of non-blue stained section/Total LV weight.*

Infarct size was then calculated as a percentage of risk area, i.e. infarct weight in each slice/Risk area weight of each slice. For these estimates and for malondialdehyde determinations (see below), rats were euthanized by removal of the heart under anesthesia at the conclusion of the experiment.

### Statistical Analysis

Data were analyzed using one-way analysis of variance and with a Bonferroni correction or by t-test analysis. Data are presented as the mean +/− the standard error (SEM).

## Supporting Information

Figure S1 **Effect of PQQ on PPARα activation.** Mouse Hepa 1–6 cells were examined to assess whether PPARα expression was responsive to the addition of PQQ. The cells were plated in 12-well plates and transfected with mouse PPARα and pSV-β-galactosidase expression vectors and PPARα luciferase reporter vector (Promega Corp., Madison, WI). The pSV-β-galactosidase vector was used to monitor transfection efficiency (Promega Corp., Madison, WI). Luciferase activities were measured using the Dual Luciferase Reporter assay system kit (Promega). Homogenates from cells were prepared with 500 µL of PLB (passive lysis buffer, Promega Corp.). Cells were lysed in agitation for 15 min. 20 µL of homogenate was used for measurement. After 16 h, cells were incubated with PQQ or 10 µM WY-14643 (a known PPAR-α agonist) for 24 h. Cell lysates were extracted and analyzed for PPAR-α activation, as detected by luciferase activity, and normalized for transactivation efficiency by β-galactosidase activity. The data represent the mean ± SD for 6 independent determinations and represents transactivation relative to the control condition (set at 100%). Means with asterisks differ from the control, as analyzed by one-way ANOVA (**, p<0.01).
(TIFF)

Table S1 **The values for individual fatty acids as components of major classes of plasma neutral and phospholipids given in Tables S1–S8.** The data are for adult rats fed PQQ- or PQQ+ diets (n =  4 to 5 per group) plus 3 additional rats fed the PQQ- diet and repleted with PQQ at 4.5 mg/kg BW (PPQ−/+) for 3 days prior to assays. To assess trends in the data, t-tests (two-tailed) were carried out.
(DOC)

Table S2 (DOC)

Table S3 (DOC)

Table S4 (DOC)

Table S5 (DOC)

Table S6 (DOC)

Table S7 (DOC)

Table S8 (DOC)
