# Supplementary figures and images for: Altering Pyrroloquinoline Quinone Nutritional Status Modulates Mitochondrial, Lipid, and Energy Metabolism in Rats
Source: PLoS One. 2011 Jul 21;6(7):e21779. doi: 10.1371/journal.pone.0021779 (PMC3140972; doi:10.1371/journal.pone.0021779)

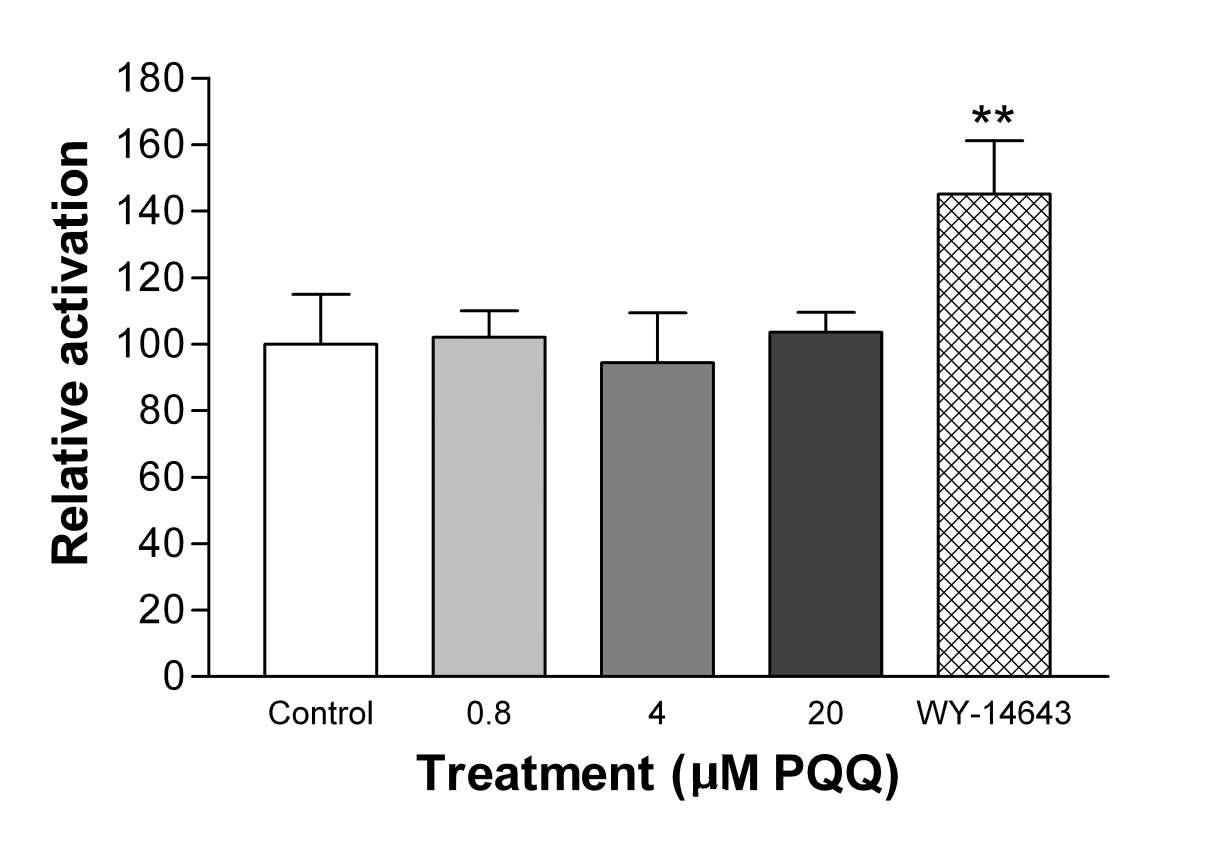

Supplement: Figure S1 — Effect of PQQ on PPARα activation. Mouse Hepa 1–6 cells were examined to assess whether PPARα expression was responsive to the addition of PQQ. The cells were plated in 12-well plates and transfected with mouse PPARα and pSV-β-galactosidase expression vectors and PPARα luciferase reporter vector (Promega Corp., Madison, WI). The pSV-β-galactosidase vector was used to monitor transfection efficiency (Promega Corp., Madison, WI). Luciferase activities were measured using the Dual Luciferase Reporter assay system kit (Promega). Homogenates from cells were prepared with 500 µL of PLB (passive lysis buffer, Promega Corp.). Cells were lysed in agitation for 15 min. 20 µL of homogenate was used for measurement. After 16 h, cells were incubated with PQQ or 10 µM WY-14643 (a known PPAR-α agonist) for 24 h. Cell lysates were extracted and analyzed for PPAR-α activation, as detected by luciferase activity, and normalized for transactivation efficiency by β-galactosidase activity. The data represent the mean ± SD for 6 independent determinations and represents transactivation relative to the control condition (set at 100%). Means with asterisks differ from the control, as analyzed by one-way ANOVA (**, p<0.01). (TIFF) [file pone.0021779.s001.tiff]
