# Supplementary material for: Altering Pyrroloquinoline Quinone Nutritional Status Modulates Mitochondrial, Lipid, and Energy Metabolism in Rats
Source: PLoS One. 2011 Jul 21;6(7):e21779. doi: 10.1371/journal.pone.0021779 (PMC3140972; doi:10.1371/journal.pone.0021779)
Supplement: Table S1 — The values for individual fatty acids as components of major classes of plasma neutral and phospholipids given in Tables S1–S8. The data are for adult rats fed PQQ- or PQQ+ diets (n = 4 to 5 per group) plus 3 additional rats fed the PQQ- diet and repleted with PQQ at 4.5 mg/kg BW (PPQ−/+) for 3 days prior to assays. To assess trends in the data, t-tests (two-tailed) were carried out. (DOC) [file pone.0021779.s002.doc]

Abbreviations: FA, fatty acid; SFA, saturated fatty acids; MUFA, mono unsaturated fatty acids; PUFA, Polyunsaturated fatty acids

| **Table 1S: Influence of PQQ on Changes in Plasma Cholesterol Ester and Constituent Fatty Acids** | | | | | | | | | | | | | | | | | | | |
| --- | --- | --- | --- | --- | --- | --- | --- | --- | --- | --- | --- | --- | --- | --- | --- | --- | --- | --- | --- |
| **Individual Fatty Acids Associated with the Cholesterol Ester Faction (nmol/g sample)** | | | | | | | | | | | | | | | | | | | |
| **FA** | **Experimental Treatments and Statistical Relationships** | | | | | | | | | | | | | | | | | | |
|  | **PQQ -/+** | | | | **PQQ+** | | | | | | **PQQ-** | | | | | | **p Value^1^** | | |
|  | **1** | **2** | **3** | **Average** | **1** | **2** | **3** | **4** | **5** | **Average** | **1** | **2** | **3** | **4** | **5** | **Average** | **PQQ+ vs**  **PQQ-** | **PQQ- vs**  **PQQ-/+** | **PQQ+ vs**  **PQQ-/+** |
| **14:0** | 14.0 | 20.3 | 23.6 | **19.3** | 20.2 | 23.4 | 27.0 | 20.0 | 21.1 | **22.3** | 31.0 | 15.7 | 19.5 | 22.3 | 20.0 | **21.7** | 0.83 | 0.57 | 0.30 |
| **15:0** | 10.6 | 17.0 | 19.8 | **15.8** | 10.3 | 16.0 | 19.7 | 19.1 | 19.4 | **16.9** | 21.1 | 14.4 | 18.5 | 18.1 | 18.4 | **18.1** | 0.59 | 0.38 | 0.73 |
| **16:0** | 131 | 135 | 148 | **138** | 107 | 104 | 156 | 120 | 132 | **124** | 91.3 | 169 | 114 | 125 | 138 | **127** | 0.82 | 0.57 | 0.32 |
| **18:0** | 21.6 | 20.3 | 29.9 | **23.9** | 29.3 | 24.0 | 16.7 | 27.8 | 20.7 | **23.7** | 11.5 | 22.7 | 18.1 | 17.7 | 31.3 | **20.3** | 0.42 | 0.48 | 0.96 |
| **20:0** | 1.1 | 3.8 | 1.6 | **2.14** | 2.7 | 2.7 | 0.7 | 1.2 | 0.8 | **1.61** | 0.3 | 0.9 | 2.1 | 0.9 | 1.2 | **1.07** | 0.35 | 0.19 | 0.55 |
| **22:0** | 0.4 | 0.2 | 0.4 | **0.33** | 0.4 | 0.3 | 0.5 | 0.4 | 0.4 | **0.403** | 0.2 | 0.6 | 0.4 | 0.3 | 0.4 | **0.372** | 0.69 | 0.71 | 0.30 |
| **24:0** | 0.1 | 0.1 | 0.1 | **0.1** | 0.6 | 0.0 | 0.1 | 0.0 | 0.1 | **0.153** | 0.1 | 0.1 | 0.2 | 0.1 | 0.2 | **0.165** | 0.92 | **0.09** | 0.72 |
| **14:1n7** | 0.6 | 0.9 | 0.9 | **0.8** | 0.6 | 0.4 | 0.9 | 0.0 | 1. | **0.583** | 0.2 | 1.0 | 0.8 | 1. | 1.0 | **0.796** | 0.38 | 0.94 | 0.38 |
| **16:1n7** | 7.6 | 9.9 | 5.9 | **7.79** | 7.1 | 8.1 | 14.7 | 9.2 | 8.7 | **9.53** | 6.1 | 10.0 | 5.5 | 6.4 | 8.6 | **7.31** | 0.20 | 0.75 | 0.41 |
| **18:1n7** | 7.0 | 6.4 | 6.7 | **6.69** | 6.0 | 7.1 | 8.5 | 6.8 | 6.2 | **6.91** | 5.3 | 8.5 | 4.8 | 6.1 | 8.3 | **6.61** | 0.74 | 0.94 | 0.72 |
| [**18:1n9**](http://www.lipomics.com/resources/fatty_acids/18_1n9.htm) | 69.4 | 65.7 | 81.8 | **72.3** | 66.2 | 99.7 | 117 | 103 | 93.5 | **95.8** | 73.8 | 94.3 | 65.9 | 102 | 98.5 | **86.9** | 0.44 | 0.20 | **0.09** |
| [**20:1n9**](http://www.lipomics.com/resources/fatty_acids/20_1n9.htm) | 0.9 | 0.3 | 0.9 | **0.72** | 1.5 | 1.1 | 1.0 | 0.9 | 0.8 | **1.06** | 0.3 | 1.1 | 0.7 | 1.4 | 1.3 | **0.974** | 0.73 | 0.44 | 0.16 |
| [**20:3n9**](http://www.lipomics.com/resources/fatty_acids/20_3n9.htm) | 0.3 | 0.3 | 0.4 | **0.33** | 0.3 | 0.4 | 0.5 | 0.3 | 0.4 | **0.382** | 0.2 | 0.4 | 0.3 | 0.5 | 0.3 | **0.329** | 0.40 | 0.98 | 0.41 |
| [**22:1n9**](http://www.lipomics.com/resources/fatty_acids/22_1n9.htm) | 0.6 | 0.8 | 1.1 | **0.85** | 1.3 | 0.4 | 0.6 | 0.4 | 1.6 | **0.831** | 0.0 | 0.5 | 0.9 | 1.2 | 0.5 | **0.629** | 0.55 | 0.49 | 0.95 |
| [**24:1n9**](http://www.lipomics.com/resources/fatty_acids/24_1n9.htm) | 0.0 | 0.0 | 0.0 | **0.00** | 0.0 | 0.0 | 0.0 | 0.0 | 0.0 | **0.00** | 1.5 | 0.0 | 0.0 | 0.0 | 0.0 | **0.308** | 0.35 | 0.48 | - |
| [**18:2n6**](http://www.lipomics.com/resources/fatty_acids/18_2n6.htm) | 272 | 274 | 326 | **291** | 192 | 212 | 320 | 226 | 263 | **243** | 211 | 335 | 229 | 272 | 257 | **261** | 0.57 | 0.38 | 0.19 |
| [**18:3n6**](http://www.lipomics.com/resources/fatty_acids/18_3n6.htm) | 7.8 | 11.4 | 7.3 | **8.84** | 6.8 | 4.8 | 11.8 | 7.7 | 7.8 | **7.78** | 4.7 | 9.0 | 5.1 | 5.5 | 6.2 | **6.08** | 0.25 | **0.10** | 0.57 |
| **20:2n6** | 0.6 | 0.4 | 0.9 | **0.64** | 0.9 | 0.9 | 0.8 | 0.7 | 0.7 | **0.814** | 0.8 | 0.9 | 0.6 | 1.1 | 1.0 | **0.865** | 0.58 | 0.15 | 0.15 |
| **20:3n6** | 2.5 | 1.7 | 2.4 | **2.22** | 2.2 | 2.1 | 2.7 | 2.3 | 2.3 | **2.30** | 3.7 | 2.8 | 1.8 | 2.3 | 2.3 | **2.58** | 0.43 | 0.47 | 0.74 |
| [**20:4n6**](http://www.lipomics.com/resources/fatty_acids/20_4n6.htm) | 842 | 768 | 836 | **815** | 552 | 477 | 902 | 499 | 730 | **632** | 542 | 908 | 595 | 641 | 714 | **680** | 0.65 | 0.17 | 0.14 |
| [**22:2n6**](http://www.lipomics.com/resources/fatty_acids/22_2n6.htm) | 0.0 | 0.0 | 0.1 | **0.04** | 0.6 | 0.0 | 0.1 | 0.1 | 0.1 | **0.170** | 3.4 | 0.0 | 0.0 | 0.0 | 0.1 | **0.699** | 0.47 | 0.50 | 0.38 |
| **22:4n6** | 0.0 | 0.0 | 0.8 | **0.26** | 2.8 | 1.2 | 0.9 | 2.0 | 0.9 | **1.55** | 0.7 | 1.8 | 0.8 | 0.8 | 0.9 | **0.996** | 0.21 | **0.06** | **0.05** |
| [**22:5n6**](http://www.lipomics.com/resources/fatty_acids/22_5n6.htm) | 11.7 | 14.1 | 15.0 | **13.6** | 13.3 | 9.4 | 13.8 | 9.9 | 14.4 | **12.2** | 6.7 | 15.2 | 11.5 | 13.1 | 10.4 | **11.4** | 0.68 | 0.33 | 0.40 |
| [**18:3n3**](http://www.lipomics.com/resources/fatty_acids/18_3n3.htm) | 0.9 | 4.8 | 1.0 | **2.23** | 1.7 | 0.9 | 1.6 | 1.3 | 0.9 | **1.28** | 0.7 | 1.10 | 3.5 | 1.5 | 1.3 | **1.65** | 0.49 | 0.63 | 0.36 |
| **18:4n3** | 0.0 | 0.0 | 0.0 | **0.00** | 0.0 | 0.0 | 0.0 | 2.8 | 2.7 | **1.10** | 0.1 | 1. | 1.2 | 0. | 2.7 | **1.00** | 0.91 | 0.18 | 0.27 |
| **20:3n3** | 0.0 | 0.0 | 0.0 | **0.00** | 0.0 | 0.0 | 0.0 | 0.0 | 0.0 | **0.00** | 0.0 | 0.0 | 0.0 | 0.0 | 0.0 | **0.00** | - | - | - |
| [**20:4n3**](http://www.lipomics.com/resources/fatty_acids/20_4n3.htm) | 0.1 | 0.0 | 0.1 | **0.064** | 0.5 | 0.8 | 0.1 | 0.1 | 0.0 | **0.309** | 0.1 | 0.1 | 0.0 | 0.0 | 0.0 | **0.0237** | **0.08** | 0.26 | 0.24 |
| [**20:5n3**](http://www.lipomics.com/resources/fatty_acids/20_5n3.htm) | 3.7 | 2.8 | 1.1 | **2.53** | 2.3 | 6.1 | 0.5 | 1.5 | 0.2 | **2.11** | 0.5 | 2. | 0.4 | 0.0 | 0.5 | **0.690** | 0.23 | **0.04** | 0.79 |
| [**22:5n3**](http://www.lipomics.com/resources/fatty_acids/22_5n3.htm) | 1.8 | 0.5 | 0.5 | **0.931** | 0.4 | 5.3 | 0.4 | 1.1 | 0.0 | **1.45** | 0.0 | 0.3 | 0.3 | 0.3 | 0.2 | **0.239** | 0.25 | **0.08** | 0.71 |
| **22:6n3** | 9.8 | 12.5 | 12.2 | **11.5** | 6.5 | 5.4 | 15. | 5.1 | 11.4 | **8.80** | 6.4 | 12.9 | 7.9 | 13.5 | 9.8 | **10.1** | 0.61 | 0.50 | 0.37 |
| **24:6n3** | 0.0 | 0.0 | 0.0 | **0.00** | 0.0 | 0.0 | 0.0 | 0.0 | 0.0 | **0.00** | 0.0 | 0.0 | 0.0 | 0.0 | 0.0 | **0.00** | - | - | - |
| [**dm16:0**](http://www.lipomics.com/resources/fatty_acids/pl_16_0.htm) | 0.0 | 0.0 | 0.0 | **0.00** | 0.0 | 0.0 | 0.0 | 0.0 | 0.0 | **0.00** | 0.0 | 0.0 | 0.0 | 0.0 | 0.0 | **0.00** | - | - | - |
| [**dm18:0**](http://www.lipomics.com/resources/fatty_acids/pl_18_0.htm) | 0.0 | 0.0 | 0.0 | **0.00** | 0.0 | 0.0 | 0.0 | 0.0 | 0.0 | **0.00** | 0.0 | 0.0 | 0.0 | 0.0 | 0.0 | **0.00** | - | - | - |
| [**dm18:1n7**](http://www.lipomics.com/resources/fatty_acids/pl_18_1n7.htm) | 0.0 | 0.0 | 0.0 | **0.00** | 0.0 | 0.0 | 0.0 | 0.0 | 0.0 | **0.00** | 0.0 | 0.0 | 0.0 | 0.0 | 0.0 | **0.00** | - | - | - |
| [**dm18:1n9**](http://www.lipomics.com/resources/fatty_acids/pl_18_1n9.htm) | 0.0 | 0.0 | 0.0 | **0.00** | 0.0 | 0.0 | 0.0 | 0.0 | 0.0 | **0.00** | 0.0 | 0.0 | 0.0 | 0.0 | 0.0 | **0.00** | - | - | - |
| [**t16:1n7**](http://www.lipomics.com/resources/fatty_acids/t16_1n7.htm) | 0.0 | 0.0 | 0.0 | **0.00** | 0.0 | 0.0 | 0.0 | 0.0 | 0.0 | **0.00** | 0.0 | 0.0 | 0.0 | 0.0 | 0.0 | **0.00** | - | - | - |
| [**t18:1n9**](http://www.lipomics.com/resources/fatty_acids/t18_1n9.htm) | 0.0 | 0.0 | 0.0 | **0.00** | 0.0 | 0.0 | 0.0 | 0.0 | 0.0 | **0.00** | 0.0 | 0.0 | 0.0 | 0.0 | 0.0 | **0.124** | 0.35 | 0.48 | - |
| **t18:2n6** | 0.6 | 2.0 | 1.0 | **1.19** | 0.8 | 0.6 | 1.4 | 0.6 | 1.3 | **0.941** | 0.0 | 1.3 | 1.5 | 1.6 | 0.6 | **0.996** | 0.88 | 0.71 | 0.54 |
| **B Total Cholesterol Ester and Fatty Acid Subclasses (nmol/g sample)^1^** | | | | | | | | | | | | | | | | | | | |
| **nmol FA/g sample** | 1420 | 1373. | 1525. | **1439** | 1037 | 1014 | 1636 | 1067 | 1342 | **1219** | 1024 | 1630 | 1111 | 1256 | 1335 | **1271** | 0.75 | 0.29 | 0.23 |
| **nmol CE/g sample** | 1420 | 1373. | 1525. | **1439** | 1037 | 1014 | 1636 | 1067 | 1342 | **1219** | 1024 | 1630 | 1111 | 1256 | 1335 | **1271** | 0.75 | 0.29 | 0.23 |
| **SFA** | 179 | 196 | 223 | **200** | 171 | 170 | 221 | 188 | 194 | **189** | 156 | 223 | 173 | 184 | 210 | **189** | 0.98 | 0.60 | 0.51 |
| **MUFA** | 86.1 | 84.1 | 97.3 | **89.2** | 82.6 | 117 | 143 | 120. | 112 | **115** | 87.2 | 116 | 78.7 | 118 | 118 | **103** | 0.41 | 0.27 | **0.10** |
| **PUFA** | 1154 | 1091 | 1204 | **1149** | 783 | 727 | 1271 | 759. | 1034 | **915** | 781 | 1291 | 858 | 952 | 1007 | **977** | 0.66 | 0.20 | 0.15 |
| **n3** | 16.3 | 20.7 | 14.8 | **17.3** | 11.4 | 18.5 | 18.2 | 11.7 | 15.3 | **15.0** | 7.8 | 17.5 | 13.4 | 15.4 | 14.6 | **13.7** | 0.57 | 0.21 | 0.39 |
| **n6** | 1138 | 1070 | 1188 | **1131** | 771 | 708 | 1252 | 747 | 1019 | **899** | 773 | 1273 | 844 | 936 | 992 | **963** | 0.65 | 0.20 | 0.15 |
| **n7** | 14.6 | 16.3 | 12.5 | **14.5** | 13.0 | 15.2 | 23.1 | 16.0 | 14.9 | **16.4** | 11.3 | 18.5 | 10.3 | 12.5 | 16.9 | **13.9** | 0.32 | 0.81 | 0.45 |
| **n9** | 71.3 | 67. | 84.3 | **74.2** | 69.2 | 102 | 119 | 104 | 96.3 | **98.1** | 75.9 | 96.3 | 67.8 | 105 | 101 | **89.1** | 0.43 | 0.20 | **0.083** |
| **dm** | 0.0 | 0.0 | 0.0 | **0.00** | 0.0 | 0.0 | 0.0 | 0.0 | 0.0 | **0.00** | 0.0 | 0.0 | 0.0 | 0.0 | 0.0 | **0.00** | - | - | - |

^1^ Values were averaged and then rounded to 3 significant numbers. p values are derived from non-adjusted t-tests to assess trends. Values for p values of 0.1 or less are highlighted in bold. The data are for adult rats fed PQQ- or PQQ+ diets (n= 4 to 5 per group) and 3 additional rats fed the PQQ- diet; repleted with PQQ 4.5 mg/kg BW (PPQ-/+) for 3 days prior to assay.
