# Supplementary material for: Altering Pyrroloquinoline Quinone Nutritional Status Modulates Mitochondrial, Lipid, and Energy Metabolism in Rats
Source: PLoS One. 2011 Jul 21;6(7):e21779. doi: 10.1371/journal.pone.0021779 (PMC3140972; doi:10.1371/journal.pone.0021779)
Supplement: Table S2 — (DOC) [file pone.0021779.s003.doc]

Abbreviations: FA, fatty acid; SFA, saturated fatty acids; MUFA, mono unsaturated fatty acids; PUFA, Polyunsaturated fatty acids

| **Table 2S: Influence of PQQ on Changes in Plasma Free Fatty Acids (nmol/g plasma)** | | | | | | | | | | | | | | | | | | | |
| --- | --- | --- | --- | --- | --- | --- | --- | --- | --- | --- | --- | --- | --- | --- | --- | --- | --- | --- | --- |
| **FA/Sample** | **Experimental Treatments and Statistical Relationships** | | | | | | | | | | | | | | | | | | |
|  | **PQQ -/+** | | | | **PQQ+** | | | | | | **PQQ-** | | | | | | **p Values^1^** | | |
|  | **1** | **2** | **3** | **Average** | **1** | **2** | **3** | **4** | **5** | **Average** | **1** | **2** | **3** | **4** | **5** | **Average** | **PQQ+ vs**  **PQQ-** | **PQQ- vs**  **PQQ-/+** | **PQQ+ vs**  **PQQ-/+** |
| **14:0** | 9.10 | 7.30 | 5.80 | **7.41** | 6.50 | 9.60 | 7.80 | 15.2 | 12.8 | **10.4** | 8.90 | 8.40 | 15.1 | 8.00 | 12.5 | **10.6** | 0.925 | 0.157 | 0.234 |
| **15:0** | 2.90 | 2.40 | 2.30 | **2.53** | 1.70 | 2.90 | 1.80 | 4.00 | 2.70 | **2.62** | 3.10 | 2.40 | 4.80 | 2.80 | 2.30 | **3.07** | 0.474 | 0.407 | 0.882 |
| **16:0** | 135 | 81.4 | 85.7 | **101** | 118 | 120 | 110 | 186 | 145 | **136** | 113 | 119 | 185 | 139 | 131 | **137** | 0.938 | 0.138 | 0.172 |
| **18:0** | 43.5 | 28.6 | 31.0 | **34.4** | 36.0 | 42.7 | 35.2 | 56.7 | 48.4 | **43.8** | 36.2 | 35.5 | 55.6 | 46.4 | 38.1 | **42.4** | 0.805 | 0.240 | 0.188 |
| **20:0** | 1.20 | 0.70 | 0.80 | **0.924** | 0.60 | 0.90 | 0.70 | 0.90 | 1.00 | **0.820** | 0.80 | 0.90 | 1.90 | 0.90 | 0.00 | **0.918** | 0.758 | 0.988 | 0.531 |
| **22:0** | 0.70 | 0.70 | 0.80 | **0.734** | 0.80 | 0.50 | 0.40 | 1.40 | 0.40 | **0.702** | 0.80 | 0.40 | 0.60 | 1.20 | 0.90 | **0.795** | 0.689 | 0.740 | 0.901 |
| **24:0** | 1.90 | 1.50 | 1.50 | **1.61** | 1.00 | 2.60 | 1.70 | 1.50 | 1.30 | **1.61** | 2.00 | 2.00 | 3.80 | 1.40 | 1.70 | **2.18** | 0.287 | 0.350 | 0.991 |
| **14:1n7** | 3.60 | 2.70 | 2.30 | **2.85** | 1.00 | 2.80 | 1.70 | 4.50 | 3.20 | **2.65** | 2.30 | 2.70 | 4.90 | 1.50 | 1.50 | **2.57** | 0.923 | 0.760 | 0.825 |
| **16:1n7** | 6.20 | 3.90 | 3.00 | **4.36** | 6.70 | 5.00 | 5.30 | 8.20 | 7.70 | **6.57** | 4.40 | 3.90 | 7.30 | 6.00 | 4.70 | **5.25** | 0.176 | 0.439 | **0.092** |
| **18:1n7** | 10.2 | 5.00 | 5.10 | **6.75** | 0.00 | 6.60 | 5.40 | 6.90 | 7.10 | **5.21** | 6.90 | 7.60 | 6.90 | 9.10 | 0.00 | **6.09** | 0.682 | 0.795 | 0.506 |
| [**18:1n9**](http://www.lipomics.com/resources/fatty_acids/18_1n9.htm) | 87.7 | 43.8 | 53.6 | **61.7** | 69.6 | 70.3 | 68.7 | 90.6 | 89.0 | **77.6** | 76.1 | 84.6 | 83.4 | 94.2 | 102 | **88.0** | 0.160 | **0.060** | 0.224 |
| [**20:1n9**](http://www.lipomics.com/resources/fatty_acids/20_1n9.htm) | 0.90 | 0.50 | 0.70 | **0.704** | 0.90 | 0.70 | 0.70 | 2.30 | 0.80 | **1.07** | 0.80 | 1.00 | 0.90 | 0.00 | 0.10 | **0.545** | 0.198 | 0.606 | 0.421 |
| [**20:3n9**](http://www.lipomics.com/resources/fatty_acids/20_3n9.htm) | 0.00 | 0.10 | 0.10 | **0.09** | 0.40 | 0.00 | 0.30 | 2.30 | 0.50 | **0.703** | 0.20 | 0.30 | 2.40 | 0.00 | 2.40 | **1.07** | 0.606 | 0.227 | 0.303 |
| [**22:1n9**](http://www.lipomics.com/resources/fatty_acids/22_1n9.htm) | 2.70 | 1.10 | 1.10 | **1.63** | 1.10 | 1.20 | 0.40 | 0.40 | 1.10 | **0.831** | 1.30 | 1.00 | 0.70 | 0.60 | 0.30 | **0.770** | 0.817 | **0.107** | 0.136 |
| [**24:1n9**](http://www.lipomics.com/resources/fatty_acids/24_1n9.htm) | 3.10 | 1.50 | 2.20 | **2.26** | 2.20 | 2.10 | 1.90 | 2.00 | 2.10 | **2.05** | 2.10 | 2.20 | 2.10 | 2.20 | 4.90 | **2.71** | 0.281 | 0.603 | 0.585 |
| [**18:2n6**](http://www.lipomics.com/resources/fatty_acids/18_2n6.htm) | 148 | 66.2 | 94.4 | **103** | 119 | 106 | 128 | 153 | 139 | **129** | 131 | 157 | 132 | 172 | 175 | **153** | **0.083** | **0.058** | 0.254 |
| [**18:3n6**](http://www.lipomics.com/resources/fatty_acids/18_3n6.htm) | 2.40 | 1.10 | 0.80 | **1.42** | 1.90 | 1.60 | 1.80 | 2.50 | 2.10 | **1.98** | 1.30 | 1.70 | 2.10 | 1.90 | 16.4 | **4.69** | 0.383 | 0.437 | 0.238 |
| **20:2n6** | 3.50 | 1.50 | 1.10 | **2.05** | 1.70 | 3.30 | 1.70 | 7.00 | 2.30 | **3.20** | 2.00 | 2.80 | 8.90 | 2.80 | 0.10 | **3.33** | 0.944 | 0.557 | 0.449 |
| **20:3n6** | 1.80 | 0.80 | 0.70 | **1.13** | 1.30 | 1.60 | 1.20 | 2.50 | 1.40 | **1.62** | 1.40 | 1.90 | 1.90 | 2.20 | 0.00 | **1.50** | 0.805 | 0.552 | 0.276 |
| [**20:4n6**](http://www.lipomics.com/resources/fatty_acids/20_4n6.htm) | 23.9 | 17.2 | 18.2 | **19.8** | 22.5 | 18.3 | 19.8 | 26.4 | 24.2 | **22.2** | 21.4 | 20.8 | 19.1 | 23.8 | 24.8 | **22.0** | 0.885 | 0.325 | 0.356 |
| [**22:2n6**](http://www.lipomics.com/resources/fatty_acids/22_2n6.htm) | 0.00 | 0.00 | 0.00 | **0.008** | 0.10 | 0.00 | 0.00 | 0.20 | 0.00 | **0.067** | 0.00 | 0.20 | 0.10 | 0.10 | 0.00 | **0.069** | 0.971 | 0.314 | 0.344 |
| **22:4n6** | 5.80 | 3.10 | 3.20 | **4.04** | 5.20 | 4.60 | 3.00 | 5.10 | 4.00 | **4.37** | 5.10 | 4.50 | 4.80 | 5.40 | 0.00 | **3.97** | 0.719 | 0.963 | 0.699 |
| [**22:5n6**](http://www.lipomics.com/resources/fatty_acids/22_5n6.htm) | 6.00 | 3.70 | 3.90 | **4.52** | 6.60 | 4.30 | 3.70 | 0.00 | 5.00 | **3.91** | 4.30 | 4.80 | 4.50 | 6.50 | 0.00 | **4.02** | 0.946 | 0.755 | 0.708 |
| [**18:3n3**](http://www.lipomics.com/resources/fatty_acids/18_3n3.htm) | 3.10 | 1.90 | 1.70 | **2.25** | 1.80 | 1.50 | 2.10 | 2.30 | 1.80 | **1.92** | 2.40 | 2.70 | 1.90 | 3.20 | 0.90 | **2.22** | 0.491 | 0.974 | 0.409 |
| **18:4n3** | 0.10 | 0.10 | 0.00 | **0.079** | 0.10 | 0.10 | 0.10 | 2.00 | 0.10 | **0.469** | 0.10 | 0.10 | 1.00 | 0.20 | 1.70 | **0.622** | 0.77 | 0.25 | 0.48 |
| **20:3n3** | 0.00 | 0.00 | 0.00 | **0.00** | 0.00 | 0.00 | 0.00 | 0.00 | 0.00 | **0.00** | 0.00 | 0.00 | 0.00 | 0.00 | 0.00 | **0.00** | - | - | - |
| [**20:4n3**](http://www.lipomics.com/resources/fatty_acids/20_4n3.htm) | 0.10 | 0.60 | 0.30 | **0.355** | 0.60 | 0.10 | 0.10 | 0.20 | 0.10 | **0.222** | 0.20 | 0.00 | 0.20 | 0.30 | 0.20 | **0.157** | 0.56 | 0.158 | 0.485 |
| [**20:5n3**](http://www.lipomics.com/resources/fatty_acids/20_5n3.htm) | 1.90 | 1.00 | 0.30 | **1.07** | 0.10 | 4.20 | 1.80 | 7.90 | 1.60 | **3.12** | 0.10 | 3.40 | 12.5 | 0.30 | 4.60 | **4.16** | 0.701 | 0.349 | 0.311 |
| [**22:5n3**](http://www.lipomics.com/resources/fatty_acids/22_5n3.htm) | 2.10 | 0.90 | 1.10 | **1.40** | 1.60 | 1.20 | 0.8 | 1.30 | 1.20 | **1.23** | 1.30 | 1.30 | 1.10 | 1.40 | 0.20 | **1.07** | 0.536 | 0.443 | 0.614 |
| **22:6n3** | 2.10 | 1.40 | 1.40 | **1.62** | 1.60 | 1.10 | 1.30 | 1.00 | 1.20 | **1.24** | 1.70 | 1.50 | 1.20 | 1.60 | 0.20 | **1.21** | 0.926 | 0.352 | 0.139 |
| **24:6n3** | 0.00 | 0.00 | 0.00 | **0.00** | 0.00 | 0.00 | 0.00 | 0.00 | 0.00 | **0.00** | 0.00 | 0.00 | 0.00 | 0.00 | 0.00 | **0.00** | - | - | - |
| [**dm16:0**](http://www.lipomics.com/resources/fatty_acids/pl_16_0.htm) | 0.00 | 0.00 | 0.00 | **0.00** | 0.00 | 0.00 | 0.00 | 0.00 | 0.00 | **0.00** | 0.00 | 0.00 | 0.00 | 0.00 | 0.00 | **0.00** | - | - | - |
| [**dm18:0**](http://www.lipomics.com/resources/fatty_acids/pl_18_0.htm) | 0.00 | 0.00 | 0.00 | **0.00** | 0.00 | 0.00 | 0.00 | 0.00 | 0.00 | **0.00** | 0.00 | 0.00 | 0.00 | 0.00 | 0.00 | **0.00** | - | - | - |
| [**dm18:1n7**](http://www.lipomics.com/resources/fatty_acids/pl_18_1n7.htm) | 0.00 | 0.00 | 0.00 | **0.00** | 0.00 | 0.00 | 0.00 | 0.00 | 0.00 | **0.00** | 0.00 | 0.00 | 0.00 | 0.00 | 0.00 | **0.00** | - | - | - |
| [**dm18:1n9**](http://www.lipomics.com/resources/fatty_acids/pl_18_1n9.htm) | 0.00 | 0.00 | 0.00 | **0.00** | 0.00 | 0.00 | 0.00 | 0.00 | 0.00 | **0.00** | 0.00 | 0.00 | 0.00 | 0.00 | 0.00 | **0.00** | - | - | - |
| [**t16:1n7**](http://www.lipomics.com/resources/fatty_acids/t16_1n7.htm) | 0.00 | 0.00 | 0.00 | **0.00** | 0.00 | 0.00 | 0.00 | 1.10 | 0.00 | **0.226** | 0.00 | 0.00 | 1.10 | 10.9 | 0.00 | **2.39** | 0.34 | 0.43 | 0.48 |
| [**t18:1n9**](http://www.lipomics.com/resources/fatty_acids/t18_1n9.htm) | 0.00 | 0.00 | 0.00 | **0.00** | 0.00 | 0.00 | 0.00 | 0.00 | 0.00 | **0.00** | 0.00 | 0.00 | 0.00 | 0.00 | 0.00 | **0.00** | - | - | - |
| **t18:2n6** | 0.00 | 0.00 | 0.00 | **0.00** | 0.00 | 0.00 | 0.00 | 0.00 | 0.00 | **0.00** | 0.00 | 0.00 | 0.00 | 0.00 | 0.00 | **0.00** | 0.51 | 0.71 | 0.82 |
| **B Total Free Fatty Acid Subclasses (nmol/g sample)^1^** | | | | | | | | | | | | | | | | | | | |
| **nmol FA/g sample** | 510 | 281 | 323 | **371** | 410 | 416 | 407 | 595 | 507 | **467** | 431 | 475 | 568 | 546 | 525 | **509** | 0.377 | **0.0658** | 0.229 |
| **SFA** | 195 | 123 | 128 | **148** | 164 | 179 | 157 | 266 | 212 | **196** | 164 | 169 | 267 | 200 | 186 | **197** | 0.95 | 0.16 | 0.18 |
| **MUFA** | 114 | 58.4 | 67.9 | **80** | 81.5 | 88.7 | 84.0 | 115 | 111 | **96.0** | 93.8 | 103 | 106 | 113 | 113 | **106** | 0.247 | 0.106 | 0.354 |
| **PUFA** | 201 | 99.9 | 127 | **142** | 164 | 148 | 165 | 214 | 184 | **175** | 173 | 203 | 194 | 221 | 226 | **204** | **0.094** | **0.055** | 0.269 |
| **n3** | 9.50 | 6.00 | 4.90 | **6.77** | 5.90 | 8.20 | 6.10 | 14.8 | 6.10 | **8.21** | 5.70 | 9.10 | 17.9 | 6.90 | 7.70 | **9.45** | 0.663 | 0.416 | 0.583 |
| **n6** | 192 | 93.8 | 122 | **136** | 158 | 140 | 159 | 197 | 178 | **166** | 167 | 194 | 174 | 215 | 216 | **193** | **0.094** | **0.064** | 0.267 |
| **n7** | 16.4 | 8.90 | 8.00 | **11.1** | 6.70 | 11.6 | 10.7 | 15.1 | 14.8 | **11.8** | 11.3 | 11.5 | 14.2 | 15.0 | 4.70 | **11.3** | 0.857 | 0.944 | 0.820 |
| **n9** | 94.4 | 47.0 | 57.7 | **66.4** | 74.1 | 74.3 | 71.9 | 97.5 | 93.5 | **82.3** | 80.5 | 89.1 | 89.5 | 96.9 | 109 | **93.1** | 0.177 | **0.073** | 0.259 |
| **dm** | 0.00 | 0.00 | 0.00 | **0.00** | 0.00 | 0.00 | 0.00 | 0.00 | 0.00 | **0.00** | 0.00 | 0.00 | 0.00 | 0.00 | 0.00 | **0.00** | - | - | - |

^1^ Values were averaged and then rounded to 3 significant numbers. p values are derived from non-adjusted t-tests to assess trends. Values for p values of 0.1 or less are highlighted in bold. The data are for adult rats fed PQQ- or PQQ+ diets (n= 4 to 5 per group) and 3 additional rats fed the PQQ- diet; repleted with PQQ 4.5 mg/kg BW (PPQ-/+) for 3 days prior to assay.
