# Supplementary material for: Altering Pyrroloquinoline Quinone Nutritional Status Modulates Mitochondrial, Lipid, and Energy Metabolism in Rats
Source: PLoS One. 2011 Jul 21;6(7):e21779. doi: 10.1371/journal.pone.0021779 (PMC3140972; doi:10.1371/journal.pone.0021779)
Supplement: Table S3 — (DOC) [file pone.0021779.s004.doc]

Abbreviations: FA, fatty acid; SFA, saturated fatty acids; MUFA, mono unsaturated fatty acids; PUFA, Polyunsaturated fatty acids

| **Table S3: Influence of PQQ on Changes in Diacylglyceride and Constituent Fatty Acids** | | | | | | | | | | | | | | | | | | | | |
| --- | --- | --- | --- | --- | --- | --- | --- | --- | --- | --- | --- | --- | --- | --- | --- | --- | --- | --- | --- | --- |
| **Individual Fatty Acids Associated with the Diacylglyceride Faction (nmol/g sample)^1^** | | | | | | | | | | | | | | | | | | | | |
| **FA/Sample** | **Experimental Treatments and Statistical Relationships** | | | | | | | | | | | | | | | | | | | |
|  | **PQQ -/+** | | | | **PQQ+** | | | | | | **PQQ-** | | | | | | | **p Values**** | | |
|  | **1** | **2** | **3** | **Average** | **1** | **2** | **3** | **4** | **5** | **Average** | | **1** | **2** | **3** | **4** | **5** | **Average** | **PQQ+ vs**  **PQQ-** | **PQQ- vs**  **PQQ-/+** | **PQQ+ vs**  **PQQ-/+** |
| 14:0 | 1.40 | 1.40 | 2.10 | **1.60** | 1.90 | 2.30 | 2.80 | 3.10 | 2.80 | **2.58** | | 2.20 | 1.90 | 3.20 | 3.30 | 2.50 | **2.62** | 0.918 | **0.0463** | **0.025** |
| 15:0 | 0.80 | 0.60 | 0.90 | **0.754** | 0.60 | 0.70 | 1.30 | 1.50 | 0.90 | **0.996** | | 1.50 | 1.00 | 1.40 | 1.10 | 1.00 | **1.21** | 0.319 | **0.0269** | 0.343 |
| 16:0 | 14.2 | 12.2 | 12.7 | **13.0** | 14.2 | 14.0 | 17.5 | 18.1 | 12.6 | **15.3** | | 30.2 | 18.9 | 16.3 | 16.1 | 18.0 | **19.9** | 0.142 | **0.0988** | 0.185 |
| 18:0 | 5.80 | 4.90 | 5.20 | **5.29** | 5.50 | 10.1 | 8.90 | 7.60 | 5.90 | **7.57** | | 13.2 | 7.50 | 7.20 | 10.8 | 8.20 | **9.38** | 0.247 | **0.0390** | **0.102** |
| 20:0 | 0.30 | 0.40 | 0.70 | **0.446** | 0.00 | 0.20 | 0.00 | 0.40 | 0.00 | **0.133** | | 0.20 | 0.50 | 0.00 | 0.10 | 0.30 | **0.225** | 0.482 | 0.184 | **0.070** |
| 22:0 | 0.40 | 0.20 | 0.00 | **0.198** | 0.00 | 0.00 | 0.00 | 0.20 | 0.00 | **0.0383** | | 0.00 | 0.70 | 0.00 | 0.00 | 1.60 | **0.454** | 0.210 | 0.560 | 0.167 |
| 24:0 | 0.20 | 0.10 | 0.10 | **0.103** | 0.00 | 0.40 | 0.20 | 0.20 | 0.00 | **0.167** | | 0.20 | 0.10 | 0.20 | 0.30 | 0.20 | **0.193** | 0.744 | **0.0708** | 0.538 |
| 14:1n7 | 0.30 | 0.30 | 0.40 | **0.335** | 0.40 | 0.70 | 0.50 | 0.60 | 0.50 | **0.534** | | 0.60 | 0.40 | 0.60 | 0.80 | 0.50 | **0.578** | 0.667 | **0.0558** | **0.066** |
| 16:1n7 | 0.70 | 0.80 | 0.40 | **0.639** | 0.70 | 0.60 | 0.70 | 0.90 | 0.00 | **0.597** | | 0.90 | 0.90 | 0.60 | 1.10 | 0.90 | **0.865** | 0.177 | 0.171 | 0.858 |
| 18:1n7 | 1.00 | 1.20 | 1.40 | **1.20** | 1.60 | 0.90 | 1.50 | 1.30 | 0.70 | **1.21** | | 3.20 | 1.80 | 1.60 | 1.10 | 2.40 | **2.03** | **0.076** | 0.143 | 0.966 |
| [18:1n9](http://www.lipomics.com/resources/fatty_acids/18_1n9.htm) | 14.5 | 10.7 | 9.30 | **11.5** | 12.4 | 9.70 | 10.4 | 15.0 | 8.10 | **11.1** | | 27.4 | 20.2 | 11.1 | 15.3 | 19.1 | **18.6** | **0.035** | 0.110 | 0.850 |
| [20:1n9](http://www.lipomics.com/resources/fatty_acids/20_1n9.htm) | 0.10 | 0.00 | 0.00 | **0.0476** | 0.00 | 0.00 | 0.00 | 0.10 | 0.00 | **0.0236** | | 0.10 | 0.00 | 0.00 | 0.00 | 0.10 | **0.04** | 0.341 | 0.929 | 0.186 |
| [20:3n9](http://www.lipomics.com/resources/fatty_acids/20_3n9.htm) | 0.10 | 0.00 | 0.00 | **0.0478** | 0.10 | 0.20 | 0.00 | 0.00 | 0.00 | **0.0606** | | 0.10 | 0.00 | 0.00 | 0.20 | 0.10 | **0.078** | 0.781 | 0.547 | 0.786 |
| [22:1n9](http://www.lipomics.com/resources/fatty_acids/22_1n9.htm) | 0.60 | 0.30 | 1.70 | **0.876** | 0.90 | 0.60 | 1.10 | 0.60 | 0.00 | **0.637** | | 0.90 | 1.20 | 0.50 | 1.40 | 0.70 | **0.940** | 0.253 | 0.870 | 0.565 |
| [24:1n9](http://www.lipomics.com/resources/fatty_acids/24_1n9.htm) | 1.10 | 0.90 | 1.10 | **1.02** | 0.70 | 0.00 | 1.00 | 1.10 | 0.90 | **0.732** | | 0.70 | 1.20 | 0.60 | 0.00 | 0.70 | **0.623** | 0.703 | 0.175 | 0.324 |
| [18:2n6](http://www.lipomics.com/resources/fatty_acids/18_2n6.htm) | 21.1 | 14.1 | 11.3 | **15.5** | 9.80 | 13.4 | 9.70 | 12.0 | 4.90 | **9.97** | | 30.8 | 34.6 | 11.1 | 15.1 | 20.9 | **22.5** | 0.0289 | 0.313 | **0.103** |
| [18:3n6](http://www.lipomics.com/resources/fatty_acids/18_3n6.htm) | 0.50 | 0.20 | 0.00 | **0.252** | 0.00 | 1.60 | 0.30 | 0.30 | 0.00 | **0.437** | | 0.70 | 0.50 | 0.30 | 1.60 | 0.50 | **0.723** | 0.476 | 0.201 | 0.673 |
| 20:2n6 | 1.00 | 0.50 | 0.30 | **0.584** | 0.00 | 1.00 | 0.30 | 0.20 | 0.20 | **0.334** | | 0.50 | 0.60 | 0.40 | 0.30 | 0.60 | **0.451** | 0.522 | 0.501 | 0.391 |
| 20:3n6 | 0.10 | 0.10 | 0.10 | **0.0935** | 0.10 | 0.10 | 0.10 | 0.00 | 0.00 | **0.0608** | | 0.20 | 0.20 | 0.00 | 0.20 | 0.20 | **0.157** | **0.060** | 0.314 | 0.244 |
| [20:4n6](http://www.lipomics.com/resources/fatty_acids/20_4n6.htm) | 7.80 | 3.60 | 3.10 | **4.80** | 3.80 | 8.80 | 3.70 | 3.60 | 4.10 | **4.81** | | 7.20 | 4.20 | 2.40 | 7.20 | 7.30 | **5.65** | 0.568 | 0.640 | 0.997 |
| [22:2n6](http://www.lipomics.com/resources/fatty_acids/22_2n6.htm) | 0.00 | 0.00 | 0.00 | **0.002** | 0.00 | 0.00 | 0.00 | 0.00 | 0.00 | **0.00655** | | 0.00 | 0.10 | 0.10 | 0.10 | 0.00 | **0.0561** | **0.096** | 0.171 | 0.467 |
| 22:4n6 | 0.40 | 0.20 | 0.10 | **0.196** | 0.20 | 0.00 | 0.10 | 0.10 | 0.00 | **0.0708** | | 0.80 | 0.40 | 0.10 | 0.00 | 0.60 | **0.374** | **0.077** | 0.418 | 0.148 |
| [22:5n6](http://www.lipomics.com/resources/fatty_acids/22_5n6.htm) | 1.20 | 0.30 | 0.10 | **0.536** | 0.20 | 0.00 | 0.10 | 0.20 | 0.00 | **0.111** | | 1.20 | 1.50 | 0.20 | 2.30 | 1.40 | **1.34** | **0.008** | 0.172 | 0.143 |
| [18:3n3](http://www.lipomics.com/resources/fatty_acids/18_3n3.htm) | 0.50 | 0.30 | 0.20 | **0.338** | 0.10 | 0.00 | 0.20 | 0.40 | 0.00 | **0.143** | | 0.30 | 1.50 | 0.30 | 3.30 | 0.20 | **1.11** | 0.149 | 0.374 | 0.131 |
| 18:4n3 | 0.00 | 0.00 | 0.00 | **0.0164** | 0.10 | 0.10 | 0.00 | 0.00 | 0.00 | **0.0387** | | 0.10 | 0.00 | 0.00 | 0.20 | 0.00 | **0.0659** | 0.580 | 0.391 | 0.567 |
| 20:3n3 | 0.00 | 0.00 | 0.00 | **0.00** | 0.00 | 0.00 | 0.00 | 0.00 | 0.00 | **0.00** | | 0.00 | 0.00 | 0.00 | 0.00 | 0.00 | **0.00** | - | - | - |
| [20:4n3](http://www.lipomics.com/resources/fatty_acids/20_4n3.htm) | 0.00 | 0.00 | 0.00 | **0.0108** | 0.00 | 0.10 | 0.00 | 0.00 | 0.00 | **0.0251** | | 0.10 | 0.00 | 0.00 | 0.00 | 0.00 | **0.0110** | 0.621 | 0.993 | 0.687 |
| [20:5n3](http://www.lipomics.com/resources/fatty_acids/20_5n3.htm) | 0.00 | 0.00 | 0.00 | **0.00** | 0.00 | 0.00 | 0.00 | 0.00 | 0.00 | **0.00681** | | 4.70 | 0.00 | 0.20 | 1.80 | 1.30 | **1.60** | **0.0934** | 0.202 | 0.482 |
| [22:5n3](http://www.lipomics.com/resources/fatty_acids/22_5n3.htm) | 0.00 | 0.40 | 0.00 | **0.129** | 1.00 | 0.00 | 0.00 | 0.00 | 0.00 | **0.210** | | 0.00 | 0.00 | 0.00 | 0.00 | 0.90 | **0.189** | 0.942 | 0.831 | 0.793 |
| 22:6n3 | 0.60 | 0.30 | 0.00 | **0.317** | 0.40 | 0.00 | 0.30 | 0.70 | 0.00 | **0.271** | | 0.70 | 0.50 | 0.50 | 1.30 | 0.80 | **0.785** | **0.0306** | **0.0960** | 0.839 |
| 24:6n3 | 0.00 | 0.00 | 0.00 | **0.00** | 0.00 | 0.00 | 0.00 | 0.00 | 0.00 | **0.00** | | 0.00 | 0.00 | 0.00 | 0.00 | 0.00 | **0.00** | - | - | - |
| [dm16:0](http://www.lipomics.com/resources/fatty_acids/pl_16_0.htm) | 0.10 | 0.00 | 0.10 | **0.101** | 0.40 | 0.00 | 0.10 | 0.00 | 2.00 | 0.507 | | 1.10 | 0.00 | 0.20 | 0.10 | 0.40 | **0.374** | 0.762 | 0.352 | - |
| [dm18:0](http://www.lipomics.com/resources/fatty_acids/pl_18_0.htm) | 0.00 | 0.00 | 0.00 | **0.00** | 0.00 | 0.00 | 0.00 | 0.00 | 0.00 | 0.00 | | 1.70 | 0.00 | 0.00 | 0.00 | 0.70 | **0.465** | 0.192 | 0.326 | 0.446 |
| [dm18:1n7](http://www.lipomics.com/resources/fatty_acids/pl_18_1n7.htm) | 0.00 | 0.00 | 0.00 | **0.00** | 0.00 | 0.00 | 0.00 | 0.00 | 0.00 | 0.00 | | 0.00 | 0.00 | 0.00 | 0.00 | 0.00 | **0.00** | - | - | - |
| [dm18:1n9](http://www.lipomics.com/resources/fatty_acids/pl_18_1n9.htm) | 0.30 | 0.20 | 0.10 | **0.224** | 0.00 | 0.00 | 0.00 | 0.00 | 0.00 | 0.00 | | 1.10 | 0.00 | 0.50 | 1.60 | 2.00 | **1.01** | 0.0208 | 0.146 | **0.002** |
| [t16:1n7](http://www.lipomics.com/resources/fatty_acids/t16_1n7.htm) | 0.00 | 0.00 | 0.00 | **0.00** | 0.00 | 0.00 | 0.00 | 0.00 | 0.30 | 0.0529 | | 0.00 | 0.00 | 0.00 | 0.00 | 0.00 | **0.00** | 0.347 | - | 0.482 |
| [t18:1n9](http://www.lipomics.com/resources/fatty_acids/t18_1n9.htm) | 0.00 | 0.00 | 0.00 | **0.00** | 0.00 | 0.00 | 0.00 | 0.00 | 0.00 | 0.00 | | 0.00 | 0.00 | 0.00 | 0.00 | 0.00 | **0.00** | - | - | - |
| t18:2n6 | 0.20 | 0.00 | 0.00 | **0.0786** | 0.00 | 0.60 | 0.00 | 0.00 | 0.00 | 0.128 | | 0.00 | 0.00 | 0.00 | 0.00 | 0.00 | **0.005** | 0.317 | 0.193 | 0.770 |
| **B Total Diacylglyceride and Fatty Acid Subclasses (nmol/g sample)^1^** | | | | | | | | | | | | | | | | | | | | |
| nmol FA/g | 75.4 | 54.1 | 51.3 | **60.3** | 55.2 | 66.2 | 60.8 | 68.4 | 43.8 | **58.9** | | 133 | 101 | 59.4 | 86.7 | 93.9 | **94.6** | **0.0217** | **0.085** | 0.868 |
| nmol CE/g sa | 37.7 | 27.1 | 25.6 | **30.1** | 27.6 | 33.1 | 30.4 | 34.2 | 21.9 | **29.4** | | 66.3 | 50.3 | 29.7 | 43.4 | 47.0 | **47.3** | **0.0217** | **0.085** | 0.868 |
| SFA | 23.0 | 19.6 | 21.6 | **21.4** | 22.2 | 27.7 | 30.6 | 31.1 | 22.1 | **26.8** | | 47.5 | 30.7 | 28.3 | 31.7 | 31.7 | **34.0** | **0.106** | **0.035** | **0.097** |
| MUFA | 18.3 | 14.3 | 14.3 | **15.6** | 16.7 | 12.6 | 15.2 | 19.6 | 10.2 | **14.9** | | 33.8 | 25.6 | 14.9 | 19.7 | 24.4 | **23.7** | **0.0373** | 0.111 | 0.755 |
| PUFA | 33.3 | 20.0 | 15.1 | **22.8** | 15.8 | 25.2 | 14.9 | 17.7 | 9.20 | **16.5** | | 47.4 | 44.2 | 15.5 | 33.6 | 34.8 | **35.1** | **0.0165** | 0.195 | 0.279 |
| n3 | 1.20 | 1.10 | 0.20 | **0.811** | 1.60 | 0.30 | 0.50 | 1.10 | 0.00 | **0.695** | | 5.80 | 2.00 | 1.00 | 6.70 | 3.30 | **3.77** | **0.026** | **0.092** | 0.805 |
| n6 | 32.1 | 18.9 | 14.8 | **22.0** | 14.1 | 24.8 | 14.4 | 16.5 | 9.20 | **15.8** | | 41.5 | 42.2 | 14.5 | 26.7 | 31.3 | **31.2** | **0.027** | 0.279 | 0.273 |
| n7 | 1.80 | 2.00 | 1.80 | **1.84** | 2.30 | 1.50 | 2.20 | 2.30 | 0.70 | **1.81** | | 4.10 | 2.70 | 2.10 | 2.20 | 3.30 | **2.89** | **0.053** | **0.077** | 0.940 |
| n9 | 16.3 | 12.0 | 12.2 | **13.5** | 14.1 | 10.5 | 12.4 | 16.8 | 9.00 | **12.6** | | 29.2 | 22.5 | 12.3 | 16.9 | 20.7 | **20.3** | **0.039** | 0.133 | 0.671 |
| dm | 0.50 | 0.20 | 0.30 | **0.326** | 0.40 | 0.00 | 0.10 | 0.00 | 2.00 | **0.507** | | 3.90 | 0.00 | 0.70 | 1.70 | 3.00 | **1.85** | 0.134 | 0.161 | 0.730 |

^1^ Values were averaged and then rounded to 3 significant numbers. p values are derived from non-adjusted t-tests to assess trends. Values for p values of 0.1 or less are highlighted in bold. The data are for adult rats fed PQQ- or PQQ+ diets (n= 4 to 5 per group) and 3 additional rats fed the PQQ- diet; repleted with PQQ 4.5 mg/kg BW (PPQ-/+) for 3 days prior to assay.
