# Supplementary material for: Altering Pyrroloquinoline Quinone Nutritional Status Modulates Mitochondrial, Lipid, and Energy Metabolism in Rats
Source: PLoS One. 2011 Jul 21;6(7):e21779. doi: 10.1371/journal.pone.0021779 (PMC3140972; doi:10.1371/journal.pone.0021779)
Supplement: Table S4 — (DOC) [file pone.0021779.s005.doc]

Abbreviations: FA, fatty acid; SFA, saturated fatty acids; MUFA, mono unsaturated fatty acids; PUFA, Polyunsaturated fatty acids

| **Table S4: Influence of PQQ on Changes in Triacylglyceride and Constituent Fatty Acids** | | | | | | | | | | | | | | | | | | | | |
| --- | --- | --- | --- | --- | --- | --- | --- | --- | --- | --- | --- | --- | --- | --- | --- | --- | --- | --- | --- | --- |
| **Individual Fatty Acids Associated with the Triacylglyceride Faction (nmol/g sample)^1^** | | | | | | | | | | | | | | | | | | | | |
| **FA/Sample #** | **Experimental Treatments and Statistical Relationships** | | | | | | | | | | | | | | | | | | | |
|  | **PQQ -/+** | | | | **PQQ+** | | | | | | **PQQ-** | | | | | | **p Values^1^** | | | |
|  | **1** | **2** | **3** | **Average** | **1** | **2** | **3** | **4** | **Average** | **1** | **2** | **3** | **4** | **5** | **Average** | | **PQQ+ vs PQQ-** | | **PQQ- vs**  **PQQ-/+** | **PQQ+ vs**  **PQQ-/+** |
| 14:0 | 12.8 | 18.2 | 8.60 | **12.3** | 16.3 | 18.6 | 11.8 | 10.1 | **12.7** | 10.1 | 8.80 | 10.7 | 9.40 | 9.00 | **10.8** | | **0.0408** | | 0.877 | 0.137 |
| 15:0 | 6.90 | 7.80 | 4.50 | **5.96** | 10.1 | 7.70 | 4.00 | 4.80 | **4.98** | 5.60 | 4.30 | 3.80 | 4.20 | 3.60 | **5.18** | | 0.169 | | 0.892 | **0.050** |
| 16:0 | 553 | 516 | 237 | **350** | 676 | 667 | 243 | 275 | **375** | 295 | 300 | 235 | 238 | 150 | **320** | | **0.082** | | 0.971 | **0.056** |
| 18:0 | 70.2 | 73.4 | 55.9 | **59.5** | 88.2 | 92.1 | 57.8 | 62.0 | **68.7** | 49.1 | 66.3 | 45.6 | 64.1 | 38.6 | **60.6** | | **0.051** | | 0.540 | 0.143 |
| 20:0 | 5.70 | 6.40 | 5.40 | **6.09** | 9.00 | 15.3 | 4.30 | 5.80 | **7.28** | 6.60 | 4.70 | 6.10 | 3.70 | 2.40 | **5.17** | | 0.161 | | 0.442 | 0.332 |
| 22:0 | 1.90 | 2.50 | 1.80 | **1.83** | 2.20 | 2.70 | 1.20 | 1.50 | **1.83** | 1.30 | 1.30 | 0.90 | 1.10 | 0.70 | **1.25** | | **0.026** | | 0.720 | **0.005** |
| 24:0 | 4.50 | 5.60 | 1.80 | **3.98** | 4.50 | 6.10 | 4.50 | 4.50 | **5.35** | 4.50 | 5.20 | 2.60 | 4.50 | 3.10 | **3.98** | | **0.105** | | 0.283 | 0.989 |
| 14:1n7 | 0.70 | 1.00 | 0.90 | **0.905** | 1.00 | 1.50 | 1.00 | 1.10 | **1.11** | 0.80 | 0.90 | 0.60 | 0.80 | 0.70 | **0.803** | | **0.008** | | 0.113 | 0.225 |
| 16:1n7 | 19.6 | 26.3 | 4.90 | **14.6** | 20.8 | 26.3 | 7.50 | 8.60 | **14.3** | 12.5 | 9.50 | 9.80 | 8.50 | 4.80 | **10.7** | | 0.124 | | 0.842 | 0.158 |
| 18:1n7 | 33.9 | 42.3 | 14.7 | **25.7** | 46.2 | 42.8 | 14.3 | 18.6 | **25.9** | 20.1 | 19.4 | 15.9 | 15.9 | 8.60 | **21.2** | | **0.073** | | 0.960 | **0.071** |
| [18:1n9](http://www.lipomics.com/resources/fatty_acids/18_1n9.htm) | 568 | 531 | 300 | **384** | 814 | 903 | 297 | 353 | **498** | 321 | 364 | 271 | 247 | 131 | **365** | | **0.048** | | 0.585 | **0.049** |
| [20:1n9](http://www.lipomics.com/resources/fatty_acids/20_1n9.htm) | 9.50 | 8.10 | 6.50 | **7.15** | 14.8 | 12.2 | 4.90 | 10.0 | **8.25** | 6.90 | 5.40 | 3.20 | 4.60 | 2.80 | **6.16** | | **0.026** | | 0.495 | **0.027** |
| [20:3n9](http://www.lipomics.com/resources/fatty_acids/20_3n9.htm) | 4.20 | 4.80 | 3.00 | **3.60** | 4.00 | 27.4 | 1.90 | 3.20 | **8.34** | 2.90 | 3.60 | 3.50 | 3.00 | 2.50 | **3.33** | | 0.322 | | 0.539 | **0.103** |
| [22:1n9](http://www.lipomics.com/resources/fatty_acids/22_1n9.htm) | 0.40 | 0.60 | 0.60 | **0.729** | 0.50 | 0.50 | 0.60 | 0.40 | **0.50** | 1.00 | 0.40 | 0.40 | 0.70 | 0.40 | **0.474** | | 0.604 | | 0.844 | 0.752 |
| [24:1n9](http://www.lipomics.com/resources/fatty_acids/24_1n9.htm) | 0.90 | 11.8 | 0.60 | **4.13** | 0.00 | 0.00 | 0.00 | 0.40 | **0.47** | 0.00 | 0.30 | 0.00 | 0.30 | 0.50 | **0.222** | | 0.794 | | 0.175 | 0.168 |
| [18:2n6](http://www.lipomics.com/resources/fatty_acids/18_2n6.htm) | 104 | 972 | 567 | **686** | 161 | 173 | 539 | 588 | **888** | 518 | 613 | 450 | 421 | 228 | **665** | | **0.051** | | 0.611 | **0.024** |
| [18:3n6](http://www.lipomics.com/resources/fatty_acids/18_3n6.htm) | 12.8 | 14.1 | 5.60 | **9.98** | 25.0 | 15.2 | 9.80 | 9.50 | **11.5** | 10.2 | 9.40 | 10.8 | 9.90 | 6.80 | **12.4** | | 0.146 | | 0.469 | 0.527 |
| 20:2n6 | 12.0 | 10.3 | 3.70 | **6.67** | 20.9 | 15.8 | 5.80 | 11.7 | **12.5** | 5.90 | 7.10 | 4.70 | 4.70 | 2.60 | **7.99** | | **0.001** | | 0.230 | 0.128 |
| 20:3n6 | 11.4 | 7.90 | 3.30 | **7.24** | 23.9 | 18.6 | 6.10 | 14.2 | **13.8** | 10.5 | 8.80 | 6.60 | 7.30 | 3.50 | **10.0** | | **0.031** | | **0.108** | 0.941 |
| [20:4n6](http://www.lipomics.com/resources/fatty_acids/20_4n6.htm) | 135 | 133 | 78.7 | **106** | 234 | 153 | 70.3 | 110 | **119** | 107 | 107 | 111 | 109 | 90.2 | **130** | | 0.243 | | 0.564 | 0.476 |
| [22:2n6](http://www.lipomics.com/resources/fatty_acids/22_2n6.htm) | 0.30 | 0.20 | 0.10 | **0.138** | 0.50 | 0.00 | 0.00 | 0.70 | **0.24** | 0.10 | 0.00 | 0.60 | 0.50 | 0.00 | **0.338** | | 0.906 | | 0.650 | 0.714 |
| 22:4n6 | 35.6 | 37.5 | 14.2 | **26.0** | 71.6 | 44.9 | 19.3 | 30.1 | **32.8** | 26.2 | 29.8 | 18.5 | 19.2 | 14.7 | **30.8** | | **0.082** | | 0.429 | 0.303 |
| [22:5n6](http://www.lipomics.com/resources/fatty_acids/22_5n6.htm) | 43.7 | 46.0 | 28.7 | **35.2** | 58.6 | 51.9 | 20.8 | 40.2 | **32.3** | 30.8 | 32.4 | 21.2 | 34.9 | 22.1 | **33.8** | | 0.208 | | 0.976 | **0.085** |
| [18:3n3](http://www.lipomics.com/resources/fatty_acids/18_3n3.htm) | 20.4 | 26.7 | 7.80 | **14.8** | 21.5 | 28.2 | 6.70 | 6.20 | **14.1** | 10.0 | 8.70 | 6.00 | 4.60 | 1.70 | **8.48** | | **0.074** | | 0.694 | **0.036** |
| 18:4n3 | 0.00 | 1.10 | 0.00 | **0.382** | 0.00 | 0.10 | 0.10 | 0.00 | **0.04** | 0.10 | 0.00 | 0.00 | 0.00 | 0.00 | **0.009** | | 0.400 | | 0.264 | 0.240 |
| 20:3n3 | 0.00 | 0.00 | 0.00 | **0.00** | 0.00 | 0.00 | 0.00 | 0.00 | **0.00** | 0.00 | 0.00 | 0.00 | 0.00 | 0.00 | **0.00** | | - | | - | - |
| [20:4n3](http://www.lipomics.com/resources/fatty_acids/20_4n3.htm) | 1.30 | 1.20 | 0.60 | **1.88** | 1.40 | 1.50 | 1.10 | 2.30 | **1.46** | 3.90 | 0.20 | 0.70 | 0.60 | 0.40 | **0.680** | | 0.645 | | 0.206 | 0.893 |
| [20:5n3](http://www.lipomics.com/resources/fatty_acids/20_5n3.htm) | 2.30 | 6.10 | 0.70 | **2.76** | 2.20 | 1.70 | 0.90 | 0.80 | **2.20** | 1.50 | 0.80 | 0.80 | 1.10 | 0.50 | **1.07** | | 0.144 | | 0.433 | 0.130 |
| [22:5n3](http://www.lipomics.com/resources/fatty_acids/22_5n3.htm) | 4.20 | 8.30 | 1.60 | **4.25** | 6.70 | 6.80 | 1.40 | 2.50 | **3.42** | 2.80 | 3.40 | 1.60 | 2.90 | 2.40 | **3.41** | | 0.229 | | 0.786 | 0.208 |
| 22:6n3 | 6.70 | 17.0 | 3.90 | **10.3** | 16.1 | 28.2 | 5.00 | 7.00 | **10.8** | 10.1 | 5.20 | 3.40 | 6.10 | 2.60 | **6.70** | | 0.155 | | 0.617 | 0.317 |
| 24:6n3 | 0.00 | 0.00 | 0.00 | **0.00** | 0.00 | 0.00 | 0.00 | 0.00 | **0.00** | 0.00 | 0.00 | 0.00 | 0.00 | 0.00 | **0.00** | | - | | - | - |
| [dm16:0](http://www.lipomics.com/resources/fatty_acids/pl_16_0.htm) | 0.60 | 0.20 | 0.30 | **0.207** | 0.30 | 1.20 | 0.30 | 1.80 | **0.77** | 0.20 | 4.60 | 0.40 | 0.20 | 0.30 | **1.17** | | 0.692 | | 0.338 | **0.503** |
| [dm18:0](http://www.lipomics.com/resources/fatty_acids/pl_18_0.htm) | 5.90 | 3.30 | 1.80 | **4.33** | 8.30 | 9.90 | 2.30 | 3.40 | **5.02** | 7.90 | 4.90 | 3.10 | 4.90 | 2.00 | **4.64** | | 0.523 | | 0.368 | 0.598 |
| [dm18:1n7](http://www.lipomics.com/resources/fatty_acids/pl_18_1n7.htm) | 0.00 | 0.00 | 0.00 | **0.037** | 0.00 | 0.00 | 0.00 | 0.00 | **0.017** | 0.00 | 0.20 | 0.00 | 0.00 | 0.00 | **0.044** | | 0.422 | | 0.256 | 0.703 |
| [dm18:1n9](http://www.lipomics.com/resources/fatty_acids/pl_18_1n9.htm) | 0.00 | 0.00 | 0.00 | **0.00** | 0.00 | 0.00 | 0.00 | 0.00 | **1.62** | 0.00 | 0.00 | 0.00 | 0.00 | 0.00 | **0.00** | | 0.347 | | 0.482 | - |
| [t16:1n7](http://www.lipomics.com/resources/fatty_acids/t16_1n7.htm) | 0.00 | 0.00 | 0.00 | **0.00** | 0.00 | 0.00 | 0.00 | 0.00 | **0.00** | 0.00 | 0.00 | 0.00 | 0.00 | 0.00 | **0.00** | | - | | - | - |
| [t18:1n9](http://www.lipomics.com/resources/fatty_acids/t18_1n9.htm) | 0.00 | 0.00 | 0.00 | **0.00** | 0.00 | 0.00 | 0.00 | 0.00 | **0.00** | 0.00 | 0.00 | 0.00 | 0.00 | 0.00 | **0.00** | | - | | - | - |
| t18:2n6 | 0.60 | 0.50 | 0.50 | **0.825** | 0.00 | 0.10 | 2.90 | 2.10 | **3.03** | 1.60 | 0.60 | 0.10 | 0.20 | 0.40 | **0.245** | | 0.168 | | 0.270 | 0.903 |
| **B Total Triglyceride and Fatty Acid Subclasses (nmol/g sample)^1^** | | | | | | | | | | | | | | | | | | | | |
| nmol FA/g sample | 2620 | 2540 | 1370 | **1800** | 3810 | 3.930 | 1350 | 1590 | **2190** | 1480 | 1630 | 1250 | 1230 | 737 | **1730** | **0.0573** | | 0.663 | | **0.0445** |
| nmol CE/g sample | 875 | 847 | 455 | **599** | 1270 | 1310 | 449 | 530 | **728** | 495 | 544 | 416 | 411 | 246 | **577** | **0.0573** | | 0.663 | | **0.0445** |
| SFA | 655 | 630 | 315 | **439** | 807 | 810 | 327 | 364 | **476** | 372 | 391 | 304 | 325 | 208 | **407** | **0.0747** | | 0.927 | | **0.0564** |
| MUFA | 633 | 621 | 328 | **437** | 898 | 986 | 325 | 392 | **549** | 363 | 400 | 301 | 278 | 149 | **405** | **0.0492** | | 0.634 | | **0.0484** |
| PUFA | 1330 | 1290 | 719 | **915** | 2100 | 2120 | 688 | 827 | **1150** | 740 | 829 | 640 | 625 | 378 | **914** | **0.0568** | | 0.596 | | **0.0369** |
| n3 | 34.9 | 60.4 | 14.6 | **34.4** | 47.9 | 66.4 | 15.1 | 18.8 | **31.9** | 28.3 | 18.3 | 12.6 | 15.3 | 7.60 | **20.3** | **0.0970** | | 0.950 | | **0.108** |
| n6 | 1290 | 1220 | 702 | **877** | 2050 | 2030 | 671 | 805 | **1110** | 709 | 808 | 624 | 607 | 368 | **891** | **0.0558** | | 0.585 | | **0.0364** |
| n7 | 53.6 | 68.5 | 19.6 | **40.3** | 67.0 | 69.0 | 21.9 | 27.2 | **40.2** | 32.7 | 28.9 | 25.7 | 24.4 | 13.5 | **31.9** | **0.0851** | | 0.913 | | **0.0993** |
| n9 | 583 | 557 | 311 | **400** | 833 | 943 | 304 | 367 | **516** | 332 | 374 | 278 | 256 | 137 | **376** | **0.0490** | | 0.592 | | **0.0462** |
| dm | 6.50 | 3.50 | 2.10 | **4.57** | 8.60 | 11.1 | 2.70 | 5.20 | **7.42** | 8.10 | 9.60 | 3.60 | 5.10 | 2.30 | **5.86** | 0.457 | | 0.187 | | 0.436 |

^1^ Values were averaged and then rounded to 3 significant numbers. p values are derived from non-adjusted t-tests to assess trends. Values for p values of 0.1 or less are highlighted in bold. The data are for adult rats fed PQQ- or PQQ+ diets (n= 4 to 5 per group) and 3 additional rats fed the PQQ- diet; repleted with PQQ 4.5 mg/kg BW (PPQ-/+) for 3 days prior to assay.
