# Supplementary material for: Altering Pyrroloquinoline Quinone Nutritional Status Modulates Mitochondrial, Lipid, and Energy Metabolism in Rats
Source: PLoS One. 2011 Jul 21;6(7):e21779. doi: 10.1371/journal.pone.0021779 (PMC3140972; doi:10.1371/journal.pone.0021779)
Supplement: Table S5 — (DOC) [file pone.0021779.s006.doc]

Abbreviations: FA, fatty acid; SFA, saturated fatty acids; MUFA, mono unsaturated fatty acids; PUFA, Polyunsaturated fatty acids

| **Table S5: Influence of PQQ on Changes in Lysophosphatidylcholine and Constituent Fatty Acids** | | | | | | | | | | | | | | | | | | | | | | | |
| --- | --- | --- | --- | --- | --- | --- | --- | --- | --- | --- | --- | --- | --- | --- | --- | --- | --- | --- | --- | --- | --- | --- | --- |
| **Individual Fatty Acids Associated with the Lysophosphatidylcholine Faction (nmol/g sample)^1^** | | | | | | | | | | | | | | | | | | | | | | | |
| **FA/Sample #** | **Experimental Treatments and Statistical Relationships** | | | | | | | | | | | | | | | | | | | | | | |
|  | **PQQ -/+** | | | | **PQQ+** | | | | | | **PQQ-** | | | | | | | | | | **p Values^1^** | | |
|  | **1** | **2** | **3** | **Average** | **1** | **2** | **3** | **4** | **5** | **Average** | **1** | | **2** | | **3** | | **4** | | **5** | **Average** | **PQQ+ vs**  **PQQ-** | **PQQ- vs**  **PQQ-/+** | **PQQ+ vs**  **PQQ-/+** |
| 14:0 | 4.80 | 2.20 | 3.90 | **3.65** | 3.50 | 2.60 | 2.30 | 3.00 | 7.40 | **3.77** | 3.00 | | 4.80 | | 3.30 | | 3.10 | | 5.50 | **3.92** | 0.884 | 0.768 | 0.937 |
| 15:0 | 3.60 | 2.80 | 3.00 | **3.14** | 2.30 | 2.30 | 2.10 | 2.00 | 5.10 | **2.74** | 2.10 | | 2.60 | | 2.40 | | 2.30 | | 4.30 | **2.72** | 0.978 | 0.501 | 0.639 |
| 16:0 | 211 | 184 | 210 | **202** | 166 | 179 | 192 | 157 | 188 | **177** | 165 | | 229 | | 172 | | 193 | | 203 | **192** | 0.268 | 0.593 | 0.0587 |
| 18:0 | 147 | 143 | 155 | **148** | 102 | 107 | 130 | 88.7 | 120 | **110** | 107 | | 137 | | 101 | | 129 | | 132 | **121** | 0.281 | 0.0344 | 0.0076 |
| 20:0 | 1.50 | 1.60 | 2.20 | **1.78** | 1.20 | 1.30 | 1.50 | 1.20 | 1.80 | **1.42** | 1.80 | | 2.00 | | 1.50 | | 1.80 | | 2.10 | **1.84** | **0.029** | 0.764 | 0.173 |
| 22:0 | 0.70 | 0.50 | 0.90 | **0.702** | 0.50 | 0.50 | 0.50 | 0.60 | 0.80 | **0.567** | 0.500 | | 0.70 | | 0.40 | | 0.50 | | 0.80 | **0.598** | 0.697 | 0.397 | 0.250 |
| 24:0 | 0.90 | 0.80 | 0.90 | **0.873** | 0.60 | 0.50 | 0.70 | 0.60 | 1.30 | **0.742** | 0.500 | | 0.70 | | 0.60 | | 0.60 | | 1.40 | **0.781** | 0.859 | 0.692 | 0.497 |
| 14:1n7 | 0.70 | 0.10 | 0.40 | **0.384** | 0.30 | 0.20 | 0.10 | 0.30 | 1.20 | **0.421** | 0.300 | | 0.30 | | 0.50 | | 0.30 | | 1.10 | **0.489** | 0.786 | 0.677 | 0.900 |
| 16:1n7 | 1.80 | 2.00 | 1.20 | **1.66** | 1.40 | 1.20 | 1.60 | 1.40 | 2.30 | **1.56** | 1.10 | | 1.80 | | 1.20 | | 1.00 | | 1.50 | **1.34** | 0.373 | 0.256 | 0.746 |
| 18:1n7 | 9.00 | 10.3 | 8.20 | **9.16** | 5.90 | 6.50 | 8.20 | 6.30 | 6.80 | **6.74** | 8.10 | | 9.10 | | 7.00 | | 6.90 | | 9.40 | **8.12** | **0.07** | 0.258 | 0.0136 |
| [18:1n9](http://www.lipomics.com/resources/fatty_acids/18_1n9.htm) | 34.6 | 26.1 | 36.4 | **32.4** | 22.2 | 22.8 | 26.1 | 29.2 | 34.0 | **26.8** | 25.0 | | 33.0 | | 26.1 | | 27.2 | | 28.6 | **28.0** | 0.672 | 0.192 | 0.189 |
| [20:1n9](http://www.lipomics.com/resources/fatty_acids/20_1n9.htm) | 1.30 | 1.60 | 1.90 | **1.57** | 1.10 | 0.80 | 1.30 | 0.80 | 1.40 | **1.07** | 1.60 | | 1.60 | | 1.30 | | 1.80 | | 1.70 | **1.59** | **0.008** | 0.881 | **0.052** |
| [20:3n9](http://www.lipomics.com/resources/fatty_acids/20_3n9.htm) | 0.10 | 0.50 | 0.00 | **0.199** | 0.10 | 0.00 | 0.00 | 0.00 | 0.10 | **0.052** | 0.200 | | 0.00 | | 0.10 | | 0.00 | | 0.10 | **0.086** | 0.386 | 0.357 | 0.207 |
| [22:1n9](http://www.lipomics.com/resources/fatty_acids/22_1n9.htm) | 0.50 | 0.50 | 0.60 | **0.502** | 2.10 | 0.20 | 0.20 | 0.50 | 0.80 | **0.767** | 0.600 | | 0.50 | | 0.40 | | 0.50 | | 0.80 | **0.555** | 0.565 | 0.625 | 0.586 |
| [24:1n9](http://www.lipomics.com/resources/fatty_acids/24_1n9.htm) | 0.00 | 0.00 | 0.10 | **0.0292** | 0.10 | 0.00 | 0.00 | 0.00 | 0.10 | **0.033** | 0.00 | | 0.00 | | 0.00 | | 0.00 | | 0.00 | **0.0369** | 0.864 | 0.580 | 0.543 |
| [18:2n6](http://www.lipomics.com/resources/fatty_acids/18_2n6.htm) | 132 | 127 | 131 | **130** | 75.8 | 90.3 | 113 | 91.2 | 80.2 | **90.2** | 108 | | 137 | | 108 | | 110 | | 116 | **116** | **0.02** | **0.1** | **0.004** |
| [18:3n6](http://www.lipomics.com/resources/fatty_acids/18_3n6.htm) | 1.00 | 1.60 | 1.00 | **1.20** | 1.00 | 1.00 | 1.50 | 1.10 | 1.10 | **1.12** | 1.00 | | 1.10 | | 0.80 | | 0.90 | | 0.90 | **0.937** | 0.110 | 0.130 | 0.704 |
| 20:2n6 | 2.10 | 3.40 | 2.40 | **2.65** | 1.30 | 1.60 | 2.00 | 1.20 | 1.60 | **1.54** | 3.30 | | 2.80 | | 2.10 | | 3.00 | | 3.50 | **2.93** | **0.0009** | 0.537 | **0.014** |
| 20:3n6 | 2.10 | 1.70 | 1.90 | **1.89** | 1.20 | 1.20 | 1.30 | 1.30 | 0.90 | **1.18** | 1.70 | | 1.40 | | 1.10 | | 1.50 | | 1.80 | **1.50** | **0.0538** | 0.0752 | **0.001** |
| [20:4n6](http://www.lipomics.com/resources/fatty_acids/20_4n6.htm) | 129 | 152 | 124 | **135** | 90.9 | 91.7 | 124 | 86.8 | 102 | **99.1** | 111 | | 120 | | 103 | | 111 | | 128 | **115** | **0.0834** | 0.0535 | **0.017** |
| [22:2n6](http://www.lipomics.com/resources/fatty_acids/22_2n6.htm) | 0.00 | 0.00 | 0.00 | **0.0272** | 0.00 | 0.10 | 0.00 | 0.00 | 0.00 | **0.02** | 0.00 | | 0.00 | | 0.00 | | 0.00 | | 0.00 | **0.026** | 0.684 | 0.909 | 0.817 |
| 22:4n6 | 1.80 | 2.70 | 2.30 | **2.28** | 1.20 | 1.20 | 1.40 | 0.90 | 1.40 | **1.24** | 2.10 | | 1.90 | | 1.30 | | 1.30 | | 2.00 | **1.72** | **0.0334** | 0.0946 | 0.003 |
| [22:5n6](http://www.lipomics.com/resources/fatty_acids/22_5n6.htm) | 10.5 | 18.3 | 13.1 | **14.0** | 10.1 | 7.30 | 10.2 | 8.10 | 10.2 | **9.17** | 10.1 | | 12.6 | | 10.1 | | 10.6 | | 11.7 | **11.0** | **0.0442** | 0.150 | 0.041 |
| [18:3n3](http://www.lipomics.com/resources/fatty_acids/18_3n3.htm) | 1.00 | 0.20 | 0.30 | **0.491** | 0.10 | 0.10 | 0.10 | 0.20 | 0.70 | **0.246** | 0.200 | | 0.40 | | 0.30 | | 0.30 | | 0.10 | **0.249** | 0.978 | 0.298 | 0.375 |
| 18:4n3 | 0.00 | 0.00 | 0.00 | **0.00293** | 0.00 | 0.00 | 0.00 | 0.00 | 0.00 | **0.004** | 0.00 | | 0.00 | | 0.00 | | 0.00 | | 0.00 | **0.002** | 0.550 | 0.922 | 0.695 |
| 20:3n3 | 0.00 | 0.00 | 0.00 | **0.00** | 0.00 | 0.00 | 0.00 | 0.00 | 0.00 | **0.00** | 0.00 | | 0.00 | | 0.00 | | 0.00 | | 0.00 | **0.00** | - | - | - |
| [20:4n3](http://www.lipomics.com/resources/fatty_acids/20_4n3.htm) | 0.10 | 0.10 | 0.10 | **0.0934** | 0.10 | 0.10 | 0.00 | 0.10 | 0.00 | **0.044** | 0.100 | | 0.10 | | 0.10 | | 0.10 | | 0.10 | **0.064** | 0.214 | **0.03** | **0.0281** |
| [20:5n3](http://www.lipomics.com/resources/fatty_acids/20_5n3.htm) | 0.20 | 0.30 | 0.10 | **0.196** | 0.00 | 0.00 | 0.00 | 0.00 | 0.00 | **0.017** | 0.00 | | 0.00 | | 0.00 | | 0.00 | | 0.00 | **0.018** | 0.864 | **0.02** | **0.0206** |
| [22:5n3](http://www.lipomics.com/resources/fatty_acids/22_5n3.htm) | 0.80 | 1.20 | 0.70 | **0.900** | 0.30 | 0.50 | 0.40 | 0.30 | 0.40 | **0.391** | 0.600 | | 0.60 | | 0.30 | | 0.30 | | 0.80 | **0.514** | 0.309 | **0.08** | **0.0056** |
| 22:6n3 | 6.60 | 8.90 | 5.70 | **7.06** | 3.50 | 5.80 | 5.10 | 3.00 | 2.80 | **4.06** | 6.00 | | 5.70 | | 4.60 | | 5.10 | | 6.60 | **5.59** | **0.0572** | 0.132 | **0.0298** |
| 24:6n3 | 0.00 | 0.00 | 0.00 | **0.00** | 0.00 | 0.00 | 0.00 | 0.00 | 0.00 | **0.00** | 0.00 | | 0.00 | | 0.00 | | 0.00 | | 0.00 | **0.00** | - | - | - |
| [dm16:0](http://www.lipomics.com/resources/fatty_acids/pl_16_0.htm) | 0.40 | 0.40 | 0.50 | **0.441** | 0.40 | 0.30 | 0.30 | 0.30 | 0.50 | **0.336** | 0.300 | | 0.50 | | 0.30 | | 0.30 | | 0.40 | **0.370** | 0.580 | 0.291 | 0.107 |
| [dm18:0](http://www.lipomics.com/resources/fatty_acids/pl_18_0.htm) | 0.60 | 0.60 | 0.60 | **0.605** | 0.50 | 2.70 | 0.50 | 0.80 | 0.60 | **1.01** | 0.500 | | 5.00 | | 0.40 | | 0.90 | | 0.40 | **1.44** | 0.676 | 0.514 | 0.495 |
| [dm18:1n7](http://www.lipomics.com/resources/fatty_acids/pl_18_1n7.htm) | 0.00 | 0.00 | 0.00 | **0.0143** | 0.00 | 0.00 | 0.00 | 0.00 | 0.00 | **0.01** | 0.00 | | 0.00 | | 0.00 | | 0.00 | | 0.00 | **0.013** | 0.167 | 0.834 | 0.283 |
| [dm18:1n9](http://www.lipomics.com/resources/fatty_acids/pl_18_1n9.htm) | 0.10 | 0.90 | 0.00 | **0.340** | 0.20 | 0.10 | 0.20 | 0.10 | 0.10 | **0.14** | 0.100 | | 0.00 | | 0.10 | | 0.10 | | 0.10 | **0.078** | **0.0299** | 0.233 | 0.383 |
| [t16:1n7](http://www.lipomics.com/resources/fatty_acids/t16_1n7.htm) | 0.00 | 0.00 | 0.00 | **0.00** | 0.00 | 0.20 | 0.00 | 0.00 | 0.00 | **0.042** | 0.200 | | 0.40 | | 0.00 | | 0.00 | | 0.00 | **0.108** | 0.446 | 0.299 | 0.482 |
| [t18:1n9](http://www.lipomics.com/resources/fatty_acids/t18_1n9.htm) | 0.00 | 0.00 | 0.00 | **0.00** | 0.00 | 0.00 | 0.00 | 0.00 | 0.00 | **0.00** | 0.00 | | 0.00 | | 0.00 | | 0.00 | | 0.00 | **0.00** | - | - | - |
| t18:2n6 | 0.00 | 0.50 | 0.10 | **0.192** | 0.00 | 0.10 | 0.10 | 0.10 | 0.00 | **0.0525** | 0.00 | | 0.00 | | 0.00 | | 0.00 | | 0.00 | **0.025** | **0.0877** | 0.155 | 0.227 |
| **B Total Lysophosphatidylcholine and Fatty Acid Subclasses (nmol/g sample)^1^** | | | | | | | | | | | | | | | | | | | | | | | |
| nmol FA/g | 706 | 696 | 709 | **704** | 496 | 530 | 627 | 487 | 574 | **543** | 562 | 712 | | 550 | | 615 | | 665 | | **621** | **0.08** | **0.08** | **0.004** |
| nmol CE/g | 706 | 696 | 709 | **704** | 496 | 530 | 627 | 487 | 574 | **543** | 562 | 712 | | 550 | | 615 | | 665 | | **621** | **0.08** | **0.08** | **0.004** |
| SFA | 369 | 335 | 376 | **360** | 276 | 294 | 329 | 253 | 324 | **295** | 279 | 377 | | 281 | | 331 | | 349 | | **324** | 0.272 | 0.224 | **0.02** |
| MUFA | 47.9 | 40.5 | 48.6 | **45.7** | 33.0 | 31.7 | 37.6 | 38.4 | 46.5 | **37.4** | 36.8 | 46.3 | | 36.5 | | 37.7 | | 43.2 | | **40.1** | 0.436 | 0.138 | **0.08** |
| PUFA | 288 | 317 | 283 | **296** | 185 | 201 | 259 | 194 | 202 | **208** | 245 | 283 | | 232 | | 245 | | 271 | | **255** | **0.02** | **0.03** | **0.004** |
| n3 | 8.70 | 10.7 | 6.80 | **8.74** | 4.00 | 6.50 | 5.60 | 3.60 | 4.00 | **4.76** | 6.90 | 6.70 | | 5.30 | | 5.70 | | 7.70 | | **6.44** | **0.04** | **0.06** | **0.01** |
| n6 | 279 | 306 | 276 | **287** | 181 | 194 | 254 | 191 | 198 | **204** | 238 | 276 | | 227 | | 239 | | 264 | | **249** | **0.02** | **0.03** | **0.004** |
| n7 | 10.8 | 12.3 | 9.40 | **10.8** | 7.20 | 7.70 | 9.80 | 7.60 | 9.10 | **8.30** | 9.20 | 11.0 | | 8.20 | | 7.90 | | 10.9 | | **9.45** | 0.192 | 0.245 | **0.03** |
| n9 | 36.5 | 28.6 | 38.9 | **34.7** | 25.5 | 23.8 | 27.7 | 30.5 | 36.3 | **28.8** | 27.4 | 35.1 | | 28.0 | | 29.5 | | 31.3 | | **30.3** | 0.582 | 0.182 | 0.162 |
| dm | 1.10 | 2.00 | 1.10 | **1.40** | 1.00 | 3.10 | 1.10 | 1.20 | 1.20 | **1.50** | 0.90 | 5.60 | | 0.80 | | 1.30 | | 0.90 | | **1.90** | 0.705 | 0.705 | 0.858 |

^1^ Values were averaged and then rounded to 3 significant numbers. p values are derived from non-adjusted t-tests to assess trends. Values for p values of 0.1 or less are highlighted in bold. The data are for adult rats fed PQQ- or PQQ+ diets (n= 4 to 5 per group) and 3 additional rats fed the PQQ- diet; repleted with PQQ 4.5 mg/kg BW (PPQ-/+) for 3 days prior to assay.
