# Supplementary material for: Altering Pyrroloquinoline Quinone Nutritional Status Modulates Mitochondrial, Lipid, and Energy Metabolism in Rats
Source: PLoS One. 2011 Jul 21;6(7):e21779. doi: 10.1371/journal.pone.0021779 (PMC3140972; doi:10.1371/journal.pone.0021779)
Supplement: Table S6 — (DOC) [file pone.0021779.s007.doc]

Abbreviations: FA, fatty acid; SFA, saturated fatty acids; MUFA, mono unsaturated fatty acids; PUFA, Polyunsaturated fatty acids

| **Table S6: Influence of PQQ on Changes in Phosphatidylcholine and Constituent Fatty Acids** | | | | | | | | | | | | | | | | | | | |
| --- | --- | --- | --- | --- | --- | --- | --- | --- | --- | --- | --- | --- | --- | --- | --- | --- | --- | --- | --- |
| **Individual Fatty Acids Associated with the Phosphatidylcholine Faction (nmol/g sample)^1^** | | | | | | | | | | | | | | | | | | | |
|  | **Experimental Treatments and Statistical Relationships** | | | | | | | | | | | | | | | | | | |
|  | **PQQ -/+** | | | | **PQQ-** | | | | | | **PQQ+** | | | | | | **p Values^1^** | | |
| **FA/Sample #** | **1** | **2** | **3** | **Average** | **1** | **2** | **3** | **4** | **5** | **Average** | **1** | **2** | **3** | **4** | **5** | **Average** | **PQQ+ vs**  **PQQ-** | **PQQ- vs**  **PQQ-/+** | **PQQ+ vs**  **PQQ-/+** |
| **14:0** | 6.20 | 4.40 | 8.40 | **6.33** | 5.30 | 3.10 | 4.70 | 5.30 | 5.10 | **4.69** | 6.40 | 7.60 | 4.00 | 1.90 | 3.80 | **4.75** | 0.960 | 0.356 | 0.152 |
| **15:0** | 5.50 | 4.80 | 7.40 | **5.88** | 5.60 | 5.00 | 5.50 | 4.40 | 4.50 | **4.99** | 4.90 | 6.00 | 4.60 | 5.90 | 9.60 | **6.20** | 0.230 | 0.813 | 0.229 |
| **16:0** | 586 | 552 | 583 | **574** | 473 | 440 | 628 | 458 | 462 | **492** | 485 | 776 | 439 | 500 | 518 | **543** | 0.477 | 0.718 | 0.131 |
| **18:0** | 540 | 481 | 514 | **512** | 387 | 352 | 522 | 363 | 374 | **400** | 418 | 595 | 360 | 434 | 438 | **449** | 0.351 | 0.285 | 0.041 |
| **20:0** | 3.20 | 3.20 | 4.40 | **3.57** | 3.00 | 2.90 | 3.90 | 2.80 | 2.60 | **3.04** | 4.40 | 5.70 | 3.50 | 5.10 | 3.70 | **4.48** | 0.016 | 0.194 | 0.261 |
| **22:0** | 1.00 | 0.80 | 1.10 | **0.991** | 0.90 | 1.00 | 1.00 | 0.90 | 1.00 | **0.940** | 0.80 | 1.10 | 5.80 | 3.10 | 0.70 | **2.31** | 0.195 | 0.348 | 0.500 |
| **24:0** | 0.90 | 0.80 | 1.70 | **1.12** | 1.20 | 1.20 | 1.10 | 0.90 | 1.50 | **1.17** | 1.00 | 1.30 | 2.30 | 0.40 | 0.70 | **1.11** | 0.867 | 0.994 | 0.832 |
| **14:1n7** | 0.00 | 0.00 | 0.10 | **0.0464** | 0.00 | 0.00 | 0.10 | 0.00 | 0.10 | **0.0462** | 0.10 | 0.00 | 0.00 | 0.00 | 0.20 | **0.070** | 0.544 | 0.653 | 0.993 |
| **16:1n7** | 4.10 | 4.60 | 2.90 | **3.86** | 3.30 | 2.90 | 4.10 | 3.30 | 3.00 | **3.33** | 3.10 | 5.00 | 0.90 | 1.70 | 2.90 | **2.72** | 0.430 | 0.300 | 0.290 |
| **18:1n7** | 32.8 | 36.2 | 29.4 | **32.8** | 25.7 | 25.1 | 37.8 | 27.0 | 24.2 | **28.0** | 36.1 | 49.2 | 8.90 | 32.4 | 35.0 | **32.3** | 0.551 | 0.955 | 0.229 |
| [**18:1n9**](http://www.lipomics.com/resources/fatty_acids/18_1n9.htm) | 79.9 | 71.4 | 87.7 | **79.7** | 70.7 | 65.8 | 85.4 | 68.9 | 63.3 | **70.8** | 77.2 | 126 | 31.5 | 77.0 | 69.8 | **76.3** | 0.735 | 0.872 | 0.203 |
| [**20:1n9**](http://www.lipomics.com/resources/fatty_acids/20_1n9.htm) | 4.60 | 4.50 | 5.40 | **4.83** | 4.10 | 4.40 | 4.90 | 3.80 | 3.60 | **4.15** | 7.00 | 8.20 | 3.70 | 6.40 | 6.50 | **6.34** | **0.021** | 0.180 | 0.112 |
| [**20:3n9**](http://www.lipomics.com/resources/fatty_acids/20_3n9.htm) | 0.60 | 0.40 | 0.60 | **0.534** | 0.60 | 1.50 | 0.50 | 0.50 | 0.40 | **0.679** | 0.40 | 0.50 | 20.2 | 0.60 | 0.30 | **4.39** | 0.376 | 0.492 | 0.604 |
| [**22:1n9**](http://www.lipomics.com/resources/fatty_acids/22_1n9.htm) | 0.30 | 0.20 | 0.50 | **0.329** | 0.30 | 1.90 | 0.30 | 0.20 | 0.50 | **0.645** | 0.30 | 0.70 | 0.00 | 0.80 | 0.40 | **0.443** | 0.569 | 0.571 | 0.477 |
| [**24:1n9**](http://www.lipomics.com/resources/fatty_acids/24_1n9.htm) | 0.20 | 0.10 | 0.30 | **0.215** | 0.20 | 0.40 | 0.60 | 0.20 | 0.40 | **0.351** | 0.10 | 0.30 | 11.0 | 7.10 | 0.10 | **3.74** | 0.172 | 0.286 | 0.221 |
| [**18:2n6**](http://www.lipomics.com/resources/fatty_acids/18_2n6.htm) | 466 | 420 | 494 | **460** | 309 | 339 | 444 | 333 | 280 | **341** | 399 | 649 | 145 | 394 | 382 | **394** | 0.550 | 0.560 | 0.025 |
| [**18:3n6**](http://www.lipomics.com/resources/fatty_acids/18_3n6.htm) | 1.50 | 1.40 | 1.30 | **1.38** | 1.70 | 1.20 | 2.60 | 1.80 | 1.50 | **1.74** | 1.50 | 2.20 | 0.40 | 1.60 | 1.30 | **1.39** | 0.374 | 0.972 | 0.281 |
| **20:2n6** | 15.6 | 15.2 | 16.8 | **15.9** | 12.3 | 13.6 | 16.1 | 11.9 | 11.8 | **13.1** | 27.5 | 28.8 | 44.1 | 23.6 | 24.9 | **29.8** | **0.002** | 0**.031** | **0.053** |
| **20:3n6** | 10.0 | 7.40 | 8.30 | **8.57** | 7.10 | 5.60 | 8.00 | 7.00 | 5.90 | **6.73** | 8.00 | 11.3 | 13.6 | 8.50 | 7.80 | **9.85** | **0.034** | 0.460 | 0.063 |
| [**20:4n6**](http://www.lipomics.com/resources/fatty_acids/20_4n6.htm) | 606 | 525 | 522 | **551** | 482 | 411 | 655 | 448 | 480 | **495** | 510 | 693 | 196 | 512 | 554 | **493** | 0.980 | 0.617 | 0.386 |
| [**22:2n6**](http://www.lipomics.com/resources/fatty_acids/22_2n6.htm) | 0.40 | 0.20 | 0.40 | **0.333** | 0.20 | 0.30 | 0.20 | 0.30 | 0.30 | **0.264** | 0.40 | 0.50 | 4.40 | 5.70 | 0.30 | **2.25** | 0.126 | 0.263 | 0.283 |
| **22:4n6** | 17.0 | 17.6 | 21.5 | **18.7** | 14.4 | 14.8 | 17.5 | 11.6 | 17.0 | **15.1** | 17.9 | 22.6 | 36.2 | 14.7 | 16.2 | **21.5** | 0.149 | 0.615 | **0.082** |
| [**22:5n6**](http://www.lipomics.com/resources/fatty_acids/22_5n6.htm) | 88.0 | 114 | 113 | **105** | 108 | 79.7 | 106 | 86.9 | 104 | **97.1** | 80.2 | 137 | 49.9 | 109 | 76.1 | **90.3** | 0.685 | 0.503 | 0.445 |
| [**18:3n3**](http://www.lipomics.com/resources/fatty_acids/18_3n3.htm) | 0.60 | 0.90 | 0.80 | **0.760** | 0.50 | 0.40 | 0.50 | 0.40 | 0.40 | **0.449** | 0.60 | 0.80 | 0.80 | 0.00 | 0.40 | **0.527** | 0.635 | 0.322 | **0.003** |
| **18:4n3** | 0.30 | 0.20 | 0.40 | **0.280** | 0.20 | 0.20 | 0.30 | 0.30 | 0.20 | **0.225** | 0.50 | 0.40 | 1.70 | 0.60 | 0.30 | **0.669** | 0.123 | 0.302 | 0.248 |
| **20:3n3** | 0.00 | 0.00 | 0.00 | **0.00** | 0.00 | 0.00 | 0.00 | 0.00 | 0.00 | **0.00** | 0.00 | 0.00 | 0.00 | 0.00 | 0.00 | **0.00** | - | - | - |
| [**20:4n3**](http://www.lipomics.com/resources/fatty_acids/20_4n3.htm) | 0.00 | 0.00 | 0.10 | **0.0566** | 0.00 | 0.00 | 0.00 | 0.00 | 0.00 | **0.0170** | 0.00 | 0.00 | 0.00 | 0.00 | 0.00 | **0.019** | 0.719 | 0.151 | 0.116 |
| [**20:5n3**](http://www.lipomics.com/resources/fatty_acids/20_5n3.htm) | 0.80 | 1.70 | 0.40 | **0.957** | 0.40 | 0.10 | 0.20 | 0.10 | 0.10 | **0.179** | 0.20 | 0.30 | 0.00 | 0.00 | 0.20 | **0.118** | 0.496 | **0.034** | 0.048 |
| [**22:5n3**](http://www.lipomics.com/resources/fatty_acids/22_5n3.htm) | 8.90 | 10.1 | 10.3 | **9.78** | 5.00 | 6.10 | 6.60 | 5.40 | 7.30 | **6.07** | 7.20 | 8.00 | 3.40 | 8.70 | 6.20 | **6.71** | 0.542 | **0.053** | **0.001** |
| **22:6n3** | 59.3 | 58.0 | 51.6 | **56.3** | 38.0 | 33.1 | 54.1 | 33.3 | 36.7 | **39.1** | 52.0 | 64.6 | 15.6 | 50.1 | 54.1 | **47.3** | 0.397 | 0.452 | **0.019** |
| **24:6n3** | 0.00 | 0.00 | 0.00 | **0.00** | 0.00 | 0.00 | 0.00 | 0.00 | 0.00 | **0.00** | 0.00 | 0.00 | 0.00 | 0.00 | 0.00 | **0.00** | - | - | - |
| [**dm16:0**](http://www.lipomics.com/resources/fatty_acids/pl_16_0.htm) | 3.70 | 2.90 | 4.40 | **3.68** | 3.00 | 1.30 | 3.90 | 2.30 | 3.20 | **2.74** | 2.40 | 4.10 | 2.00 | 2.50 | 3.00 | **2.82** | 0.895 | 0.186 | 0.210 |
| [**dm18:0**](http://www.lipomics.com/resources/fatty_acids/pl_18_0.htm) | 1.50 | 1.10 | 2.10 | **1.57** | 0.80 | 1.10 | 1.30 | 1.00 | 1.30 | **1.10** | 1.00 | 1.80 | 1.30 | 6.60 | 1.60 | **2.43** | 0.240 | 0.563 | **0.091** |
| [**dm18:1n7**](http://www.lipomics.com/resources/fatty_acids/pl_18_1n7.htm) | 0.10 | 0.10 | 0.10 | **0.01** | 0.10 | 0.60 | 0.00 | 0.00 | 0.10 | **0.156** | 0.10 | 0.10 | 0.00 | 0.00 | 3.30 | **0.698** | 0.430 | 0.499 | 0.626 |
| [**dm18:1n9**](http://www.lipomics.com/resources/fatty_acids/pl_18_1n9.htm) | 0.90 | 0.80 | 1.30 | **0.979** | 1.20 | 0.50 | 0.80 | 0.30 | 0.50 | **0.665** | 0.20 | 0.50 | 0.40 | 3.50 | 0.50 | **1.03** | 0.591 | 0.958 | 0.214 |
| [**t16:1n7**](http://www.lipomics.com/resources/fatty_acids/t16_1n7.htm) | 0.50 | 0.00 | 0.70 | **0.408** | 0.50 | 0.20 | 0.50 | 0.10 | 0.60 | **0.385** | 0.50 | 0.70 | 15.1 | 14.1 | 1.30 | **6.36** | 0.115 | 0.235 | 0.913 |
| [**t18:1n9**](http://www.lipomics.com/resources/fatty_acids/t18_1n9.htm) | 0.00 | 0.00 | 0.00 |  | 0.00 | 0.00 | 0.00 | 0.00 | 0.00 | **0.00** | c | 0.00 | 0.00 | 0.00 | 0.00 | 0.00 | - | - | - |
| **t18:2n6** | 0.10 | 0.30 | 0.30 | **0.231** | 0.10 | 0.40 | 0.30 | 0.40 | 0.30 | **0.313** | 0.40 | 0.40 | 29.7 | 0.20 | 0.30 | **6.20** | 0.345 | 0.474 | 0.381 |
| **B Phosphatidylcholine and Fatty Acid Subclasses (nmol/g sample)^1^** | | | | | | | | | | | | | | | | | | | |
| **nmol FA/g sample** | 2550 | 2340 | 2500 | **2460** | 197 | 1820 | 2620 | 1880 | 1900 | **2040** | 2.15 | 3.21 | 1.46 | 2.23 | 2.22 | **2250** | 0.510 | 0.600 | **0.0794** |
| **nmol CE/g sample** | 1270 | 1170 | 1250 | **1230** | 983 | 908 | 1310 | 942 | 949 | **1020** | 1080 | 1600 | 728 | 1120 | 1110 | **1130** | 0.510 | 0.600 | **0.0794** |
| **SFA** | 1140 | 1050 | 1120 | **1100** | 876 | 805 | 1170 | 835 | 850 | **907** | 920 | 1390 | 820 | 950 | 975 | **1010** | 0.404 | 0.516 | **0.0723** |
| **MUFA** | 122 | 117 | 126 | **122** | 104 | 101 | 133 | 103 | 95.2 | **107** | 124 | 189 | 56.1 | 125 | 115 | **122** | 0.529 | 0.997 | 0.161 |
| **PUFA** | 1270 | 1170 | 1240 | **1230** | 979 | 906 | 1310 | 941 | 946 | **1020** | 1100 | 1620 | 531 | 1130 | 1120 | **1100** | 0.665 | 0.598 | **0.0823** |
| **n3** | 69.9 | 70.9 | 63.6 | **68.1** | 44.1 | 40.0 | 61.7 | 39.6 | 44.7 | **46.0** | 60.4 | 74.0 | 21.6 | 59.4 | 61.3 | **55.3** | 0.367 | 0.324 | **0.008** |
| **n6** | 1200 | 1100 | 1180 | **1160** | 935 | 865 | 1250 | 901 | 901 | **970** | 1040 | 1540 | 490 | 1070 | 1060 | **1040** | 0.704 | 0.613 | **0.097** |
| **n7** | 37.0 | 40.8 | 32.3 | **36.7** | 29.0 | 27.9 | 41.9 | 30.3 | 27.2 | **31.3** | 39.1 | 54.2 | 9.80 | 34.1 | 37.9 | **35.0** | 0.638 | 0.871 | 0.228 |
| **n9** | 85.7 | 76.7 | 94.4 | **85.6** | 75.9 | 74.0 | 91.6 | 73.6 | 68.3 | **76.7** | 85.0 | 136 | 66.4 | 91.8 | 77.1 | **91.2** | 0.280 | 0.744 | 0.215 |
| **dm** | 6.20 | 4.90 | 7.80 | **6.31** | 5.10 | 3.50 | 6.00 | 3.60 | 5.20 | **4.66** | 3.70 | 6.40 | 3.70 | 12.6 | 8.40 | **6.98** | 0.217 | 0.781 | 0.116 |

^1^ Values were averaged and then rounded to 3 significant numbers. p values are derived from non-adjusted t-tests to assess trends. Values for p values of 0.1 or less are highlighted in bold. The data are for adult rats fed PQQ- or PQQ+ diets (n= 4 to 5 per group) and 3 additional rats fed the PQQ- diet; repleted with PQQ 4.5 mg/kg BW (PPQ-/+) for 3 days prior to assay.
