# Supplementary material for: Altering Pyrroloquinoline Quinone Nutritional Status Modulates Mitochondrial, Lipid, and Energy Metabolism in Rats
Source: PLoS One. 2011 Jul 21;6(7):e21779. doi: 10.1371/journal.pone.0021779 (PMC3140972; doi:10.1371/journal.pone.0021779)
Supplement: Table S7 — (DOC) [file pone.0021779.s008.doc]

Abbreviations: FA, fatty acid; SFA, saturated fatty acids; MUFA, mono unsaturated fatty acids; PUFA, Polyunsaturated fatty acids

| **Table S7: Influence of PQQ on Changes in Phosphatidylethanolamine and Constituent Fatty Acids** | | | | | | | | | | | | | | | | | | | |
| --- | --- | --- | --- | --- | --- | --- | --- | --- | --- | --- | --- | --- | --- | --- | --- | --- | --- | --- | --- |
| **Individual Fatty Acids Associated with the Phosphatidylethanolamine Faction (nmol/g sample)^1^** | | | | | | | | | | | | | | | | | | | |
| **FA/Sample #** | **Experimental Treatments and Statistical Relationships** | | | | | | | | | | | | | | | | | | |
|  | **PQQ -/+** | | | | **PQQ+** | | | | | | **PQQ-** | | | | | | **p Values^1^** | | |
|  | **1** | **2** | **3** | **Average** | **1** | **2** | **3** | **4** | **5** | **Average** | **1** | **2** | **3** | **4** | **5** | **Average** | **PQQ+ vs**  **PQQ-** | **PQQ- vs**  **PQQ-/+** | **PQQ+ vs**  **PQQ-/+** |
| **14:0** | 9.40 | 1.30 | 7.90 | **6.21** | 5.50 | 6.70 | 4.70 | 6.20 | 7.50 | **6.14** | 6.10 | 7.60 | 3.70 | 10.6 | 5.50 | **6.70** | 0.665 | 0.844 | 0.970 |
| **15:0** | 3.60 | 1.30 | 2.00 | **2.31** | 2.00 | 1.90 | 1.70 | 2.30 | 2.10 | **1.99** | 1.60 | 2.50 | 1.40 | 4.30 | 1.40 | **2.25** | 0.665 | 0.950 | 0.573 |
| **16:0** | 61.6 | 33.4 | 46.2 | **47.1** | 34.8 | 32.6 | 37.8 | 61.3 | 38.1 | **40.9** | 33.6 | 53.2 | 27.3 | 50.2 | 34.2 | **39.7** | 0.873 | 0.443 | 0.524 |
| **18:0** | 139 | 147 | 139 | **142** | 115 | 81.2 | 144 | 111 | 118 | **114** | 97.2 | 162 | 95.1 | 98.8 | 108 | **112** | 0.928 | 0.134 | 0.083 |
| **20:0** | 0.90 | 0.70 | 1.00 | **0.87** | 0.80 | 0.70 | 0.80 | 0.70 | 0.80 | **0.76** | 0.80 | 1.20 | 0.90 | 1.40 | 0.70 | **0.993** | 0.112 | 0.506 | 0.099 |
| **22:0** | 0.70 | 1.20 | 0.70 | **0.858** | 1.10 | 0.90 | 0.50 | 0.40 | 0.80 | **0.74** | 0.80 | 1.60 | 1.10 | 0.80 | 1.10 | **1.09** | 0.108 | 0.354 | 0.592 |
| **24:0** | 0.90 | 2.50 | 1.40 | **1.61** | 2.00 | 1.60 | 1.60 | 1.40 | 1.80 | **1.69** | 1.50 | 2.40 | 1.40 | 2.30 | 1.50 | **1.83** | 0.577 | 0.645 | 0.847 |
| **14:1n7** | 1.70 | 1.60 | 1.70 | **1.67** | 1.60 | 2.10 | 1.50 | 0.80 | 1.80 | **1.56** | 2.80 | 1.90 | 2.30 | 1.80 | 1.50 | **2.04** | 0.151 | 0.251 | 0.700 |
| **16:1n7** | 2.90 | 0.50 | 2.50 | **1.95** | 0.40 | 2.60 | 2.10 | 1.70 | 2.80 | **1.91** | 2.20 | 2.70 | 1.70 | 2.70 | 2.00 | **2.26** | 0.466 | 0.616 | 0.956 |
| **18:1n7** | 2.60 | 1.90 | 3.30 | **2.63** | 1.50 | 2.10 | 2.70 | 2.00 | 2.90 | **2.24** | 2.70 | 3.60 | 1.80 | 2.60 | 2.40 | **2.61** | 0.358 | 0.968 | 0.403 |
| [**18:1n9**](http://www.lipomics.com/resources/fatty_acids/18_1n9.htm) | 45.2 | 15.9 | 51.1 | **37.4** | 15.3 | 44.6 | 43.5 | 33.4 | 46.8 | **36.7** | 44.3 | 56.7 | 37.3 | 55.2 | 39.9 | **46.7** | 0.195 | 0.369 | 0.952 |
| [**20:1n9**](http://www.lipomics.com/resources/fatty_acids/20_1n9.htm) | 0.50 | 0.70 | 1.10 | **0.76** | 0.50 | 2.40 | 0.80 | 0.70 | 0.90 | **1.05** | 0.90 | 1.10 | 0.70 | 1.20 | 0.70 | **0.914** | 0.712 | 0.432 | 0.559 |
| [**20:3n9**](http://www.lipomics.com/resources/fatty_acids/20_3n9.htm) | 0.10 | 0.40 | 0.30 | **0.278** | 0.30 | 0.40 | 0.30 | 0.20 | 0.30 | **0.29** | 0.20 | 0.50 | 0.30 | 0.30 | 0.20 | **0.289** | 0.942 | 0.909 | 0.821 |
| [**22:1n9**](http://www.lipomics.com/resources/fatty_acids/22_1n9.htm) | 1.80 | 0.80 | 2.50 | **1.72** | 0.30 | 9.50 | 1.90 | 2.30 | 2.80 | **3.34** | 2.20 | 2.80 | 1.70 | 2.70 | 1.90 | **2.27** | 0.520 | 0.275 | 0.478 |
| [**24:1n9**](http://www.lipomics.com/resources/fatty_acids/24_1n9.htm) | 0.10 | 1.40 | 0.20 | **0.588** | 0.30 | 0.60 | 0.50 | 0.40 | 0.40 | **0.45** | 0.20 | 0.40 | 1.70 | 0.40 | 0.30 | **0.579** | 0.667 | 0.985 | 0.693 |
| [**18:2n6**](http://www.lipomics.com/resources/fatty_acids/18_2n6.htm) | 29.3 | 26.9 | 33.5 | **29.9** | 18.1 | 17.4 | 25.7 | 16.5 | 18.6 | **19.3** | 24.5 | 39.9 | 21.9 | 25.9 | 20.9 | **26.6** | 0.090 | 0.516 | 0.0066 |
| [**18:3n6**](http://www.lipomics.com/resources/fatty_acids/18_3n6.htm) | 0.00 | 0.80 | 0.10 | **0.300** | 0.50 | 0.70 | 0.30 | 1.00 | 0.60 | **0.619** | 0.40 | 0.70 | 0.30 | 0.00 | 0.40 | **0.351** | 0.131 | 0.830 | 0.217 |
| **20:2n6** | 0.80 | 1.70 | 1.30 | **1.27** | 0.90 | 0.80 | 1.10 | 1.00 | 1.20 | **1.01** | 1.20 | 1.50 | 2.30 | 1.00 | 1.00 | **1.42** | 0.145 | 0.701 | 0.225 |
| **20:3n6** | 1.50 | 2.00 | 1.80 | **1.78** | 1.20 | 0.90 | 1.60 | 1.30 | 1.40 | **1.29** | 1.30 | 2.30 | 1.10 | 1.20 | 1.30 | **1.45** | 0.519 | 0.304 | 0.0442 |
| [**20:4n6**](http://www.lipomics.com/resources/fatty_acids/20_4n6.htm) | 136 | 163 | 137 | **145** | 115 | 76.5 | 149 | 97.0 | 119 | **111** | 97.2 | 165 | 100 | 90.2 | 109 | **112** | 0.962 | 0.133 | 0.097 |
| [**22:2n6**](http://www.lipomics.com/resources/fatty_acids/22_2n6.htm) | 0.00 | 0.00 | 0.20 | **0.08** | 0.20 | 0.00 | 0.20 | 0.00 | 0.20 | **0.107** | 0.10 | 0.20 | 0.30 | 0.20 | 0.20 | **0.211** | 0.071 | 0.063 | 0.755 |
| **22:4n6** | 5.80 | 8.10 | 7.80 | **7.28** | 5.70 | 4.60 | 6.70 | 4.60 | 6.70 | **5.66** | 5.80 | 8.50 | 5.60 | 4.60 | 5.80 | **6.05** | 0.636 | 0.269 | 0.0938 |
| [**22:5n6**](http://www.lipomics.com/resources/fatty_acids/22_5n6.htm) | 5.60 | 9.60 | 7.90 | **7.68** | 7.50 | 4.20 | 7.60 | 5.60 | 7.60 | **6.51** | 4.80 | 8.10 | 5.90 | 6.00 | 4.70 | **5.92** | 0.535 | 0.184 | 0.389 |
| [**18:3n3**](http://www.lipomics.com/resources/fatty_acids/18_3n3.htm) | 0.50 | 0.20 | 0.30 | **0.34** | 0.20 | 0.20 | 0.20 | 0.20 | 0.20 | **0.21** | 0.20 | 0.40 | 0.30 | 0.30 | 0.20 | **0.292** | 0.067 | 0.543 | 0.0589 |
| **18:4n3** | 0.10 | 0.00 | 0.00 | **0.045** | 0.00 | 0.00 | 0.00 | 0.00 | 0.00 | **0.00** | 0.00 | 0.00 | 0.00 | 0.00 | 0.00 | **0.00** | - | 0.220 | 0.22 |
| **20:3n3** | 0.00 | 0.00 | 0.00 | **0.00** | 0.00 | 0.00 | 0.00 | 0.00 | 0.00 | **0.00** | 0.00 | 0.00 | 0.00 | 0.00 | 0.00 | **0.00** | - | - | - |
| [**20:4n3**](http://www.lipomics.com/resources/fatty_acids/20_4n3.htm) | 0.20 | 0.20 | 0.10 | **0.181** | 0.20 | 0.30 | 0.10 | 0.20 | 0.00 | **0.157** | 0.00 | 0.20 | 0.20 | 0.30 | 0.10 | **0.165** | 0.906 | 0.823 | 0.77 |
| [**20:5n3**](http://www.lipomics.com/resources/fatty_acids/20_5n3.htm) | 0.00 | 1.60 | 0.00 | **0.521** | 0.40 | 0.00 | 0.00 | 0.00 | 0.00 | **0.085** | 0.00 | 0.00 | 4.00 | 0.00 | 1.60 | **1.13** | 0.223 | 0.605 | 0.314 |
| [**22:5n3**](http://www.lipomics.com/resources/fatty_acids/22_5n3.htm) | 1.60 | 2.80 | 2.20 | **2.20** | 1.20 | 1.00 | 1.50 | 0.90 | 1.20 | **1.17** | 1.00 | 1.70 | 0.90 | 1.10 | 1.20 | **1.19** | 0.899 | **0.018** | 0.0097 |
| **22:6n3** | 4.50 | 7.10 | 6.80 | **6.14** | 4.50 | 2.60 | 5.60 | 3.10 | 4.10 | **3.99** | 4.30 | 5.50 | 3.70 | 4.10 | 5.00 | **4.54** | 0.403 | **0.072** | 0.0584 |
| **24:6n3** | 0.00 | 0.00 | 0.00 | **0.00** | 0.00 | 0.00 | 0.00 | 0.00 | 0.00 | **0.00** | 0.00 | 0.00 | 0.00 | 0.00 | 0.00 | **0.00** | - | - | - |
| [**dm16:0**](http://www.lipomics.com/resources/fatty_acids/pl_16_0.htm) | 3.70 | 3.20 | 4.20 | **3.70** | 2.70 | 1.50 | 3.30 | 2.50 | 3.50 | **2.69** | 2.70 | 3.80 | 2.60 | 2.60 | 2.90 | **2.92** | 0.593 | 0.08 | 0.092 |
| [**dm18:0**](http://www.lipomics.com/resources/fatty_acids/pl_18_0.htm) | 5.90 | 4.90 | 6.20 | **5.66** | 5.70 | 4.60 | 5.50 | 11.0 | 4.90 | **6.34** | 4.40 | 7.00 | 3.70 | 5.10 | 4.30 | **4.90** | 0.306 | 0.379 | 0.690 |
| [**dm18:1n7**](http://www.lipomics.com/resources/fatty_acids/pl_18_1n7.htm) | 0.30 | 0.30 | 0.00 | **0.20** | 0.20 | 0.00 | 0.20 | 0.10 | 0.20 | **0.122** | 0.10 | 0.20 | 0.40 | 0.10 | 0.20 | **0.190** | 0.320 | 0.956 | 0.410 |
| [**dm18:1n9**](http://www.lipomics.com/resources/fatty_acids/pl_18_1n9.htm) | 3.30 | 3.60 | 3.00 | **3.31** | 2.20 | 1.50 | 2.40 | 2.20 | 1.80 | **2.03** | 1.80 | 2.30 | 3.60 | 2.30 | 2.20 | **2.45** | 0.258 | **0.087** | **0.0019** |
| [**t16:1n7**](http://www.lipomics.com/resources/fatty_acids/t16_1n7.htm) | 0.00 | 0.00 | 0.00 | **0.00** | 2.90 | 0.00 | 3.40 | 0.00 | 0.00 | **1.26** | 3.00 | 0.00 | 0.00 | 0.00 | 0.00 | **0.607** | 0.528 | 0.482 | 0.269 |
| [**t18:1n9**](http://www.lipomics.com/resources/fatty_acids/t18_1n9.htm) | 0.00 | 0.00 | 0.00 | **0.00** | 0.00 | 0.00 | 0.00 | 0.00 | 0.00 | **0.00** | 0.00 | 0.00 | 0.00 | 0.00 | 0.00 | **0.00** | - | - | - |
| **t18:2n6** | 0.00 | 0.10 | 0.00 | **0.041** | 0.00 | 0.50 | 0.10 | 0.80 | 0.10 | **0.313** | 0.10 | 0.20 | 0.50 | 0.10 | 0.00 | **0.194** | 0.515 | 0.213 | 0.240 |
| **B Total Phosphatidylethanolamine and Fatty Acid Subclasses (nmol/g sample)^1^** | | | | | | | | | | | | | | | | | | | |
| **nmol FA/g sample** | 471 | 446 | 474 | **464** | 350 | 307 | 459 | 373 | 400 | **378** | 350 | 547 | 336 | 381 | 362 | **395** | 0.714 | 0.237 | **0.0465** |
| **nmol CE/g sample** | 235 | 223 | 237 | **232** | 175 | 154 | 229 | 186 | 200 | **189** | 175 | 274 | 168 | 190 | 181 | **198** | 0.714 | 0.237 | **0.0465** |
| **SFA** | 217 | 187 | 198 | **201** | 161 | 126 | 191 | 183 | 169 | **166** | 142 | 231 | 131 | 168 | 152 | **165** | 0.957 | 0.191 | **0.0795** |
| **MUFA** | 54.8 | 22.9 | 62.5 | **46.7** | 20.0 | 63.8 | 52.9 | 41.1 | 58.4 | **47.3** | 55.3 | 69.1 | 47.2 | 66.6 | 48.7 | **57.4** | 0.294 | 0.359 | 0.970 |
| **PUFA** | 186 | 224 | 200 | **203** | 155 | 110 | 200 | 132 | 161 | **152** | 141 | 234 | 147 | 135 | 152 | **162** | 0.680 | 0.157 | **0.0553** |
| **n3** | 7.00 | 11.9 | 9.40 | **9.42** | 6.40 | 4.20 | 7.40 | 4.50 | 5.60 | **5.61** | 5.60 | 7.90 | 9.10 | 5.90 | 8.10 | **7.32** | **0.097** | 0.174 | **0.027** |
| **n6** | 179 | 212 | 190 | **194** | 149 | 105 | 192 | 127 | 156 | **146** | 135 | 226 | 137 | 129 | 143 | **154** | 0.725 | 0.167 | **0.06** |
| **n7** | 5.50 | 2.50 | 5.80 | **4.58** | 1.90 | 4.70 | 4.80 | 3.70 | 5.60 | **4.15** | 4.90 | 6.20 | 3.50 | 5.30 | 4.40 | **4.87** | 0.384 | 0.779 | 0.718 |
| **n9** | 47.7 | 19.3 | 55.3 | **40.8** | 16.7 | 57.5 | 46.9 | 36.9 | 51.3 | **41.9** | 47.7 | 61.4 | 41.8 | 59.7 | 43.0 | **50.7** | 0.313 | 0.345 | 0.933 |
| **dm** | 13.3 | 12.0 | 13.4 | **12.9** | 10.8 | 7.70 | 11.3 | 15.9 | 10.3 | **11.2** | 9.10 | 13.2 | 10.3 | 10.2 | 9.50 | **10.5** | 0.646 | 0.057 | 0.384 |

^1^ Values were averaged and then rounded to 3 significant numbers. p values are derived from non-adjusted t-tests to assess trends. Values for p values of 0.1 or less are highlighted in bold. The data are for adult rats fed PQQ- or PQQ+ diets (n= 4 to 5 per group) and 3 additional rats fed the PQQ- diet; repleted with PQQ 4.5 mg/kg BW (PPQ-/+) for 3 days prior to assay.
