# Supplementary material for: Altering Pyrroloquinoline Quinone Nutritional Status Modulates Mitochondrial, Lipid, and Energy Metabolism in Rats
Source: PLoS One. 2011 Jul 21;6(7):e21779. doi: 10.1371/journal.pone.0021779 (PMC3140972; doi:10.1371/journal.pone.0021779)
Supplement: Table S8 — (DOC) [file pone.0021779.s009.doc]

| **Table 8S: Influence of PQQ on Changes in Sphingomyelin and Constituent Fatty Acids** | | | | | | | | | | | | | | | | | | | |
| --- | --- | --- | --- | --- | --- | --- | --- | --- | --- | --- | --- | --- | --- | --- | --- | --- | --- | --- | --- |
| **Individual Fatty Acids Associated with the Sphingomyelin Faction (nmol/g sample)^1^** | | | | | | | | | | | | | | | | | | | |
| **FA/Sample #** | **Experimental Treatments and Statistical Relationships** | | | | | | | | | | | | | | | | | | |
|  | **PQQ -/+** | | | | **PQQ+** | | | | | | **PQQ-** | | | | | | **p Values^1^** | | |
|  | **1** | **2** | **3** | **Average** | **1** | **2** | **3** | **4** | **5** | **Average** | **1** | **2** | **3** | **4** | **5** | **Average** | **PQQ+ vs**  **PQQ-** | **PQQ- vs**  **PQQ-/+** | **PQQ+ vs**  **PQQ-/+** |
| **14:0** | 7.30 | 4.40 | 2.30 | **4.66** | 2.70 | 1.60 | 2.80 | 2.60 | 4.80 | **2.90** | 3.20 | 2.00 | 8.80 | 3.10 | 4.20 | **4.27** | 0.330 | 0.840 | 0.210 |
| **15:0** | 1.50 | 1.20 | 6.00 | **2.91** | 0.60 | 1.00 | 0.90 | 1.10 | 6.50 | **2.03** | 1.50 | 0.80 | 5.70 | 1.10 | 0.50 | **1.92** | 0.940 | 0.580 | 0.660 |
| **16:0** | 113 | 31.4 | 119 | **88.0** | 11.6 | 71.7 | 72.0 | 52.7 | 66.5 | **54.9** | 52.6 | 29.1 | 79.4 | 68.7 | 11.4 | **48.2** | 0.700 | 0.190 | 0.240 |
| **18:0** | 52.3 | 18.2 | 27.9 | **32.8** | 5.00 | 31.2 | 25.8 | 23.6 | 33.4 | **23.8** | 22.2 | 13.8 | 25.0 | 30.9 | 4.30 | **19.2** | 0.520 | 0.210 | 0.400 |
| **20:0** | 8.70 | 2.00 | 4.70 | **5.11** | 0.40 | 4.80 | 7.90 | 4.70 | 5.20 | **4.59** | 4.30 | 2.30 | 2.60 | 5.30 | 0.40 | **2.98** | 0.300 | 0.280 | 0.820 |
| **22:0** | 55.4 | 6.80 | 33.4 | **31.9** | 1.20 | 28.2 | 27.2 | 31.3 | 31.1 | **23.8** | 28.8 | 8.00 | 6.30 | 32.7 | 1.30 | **15.4** | 0.360 | 0.260 | 0.550 |
| **24:0** | 17.5 | 5.10 | 59.5 | **27.4** | 1.40 | 17.4 | 14.8 | 14.0 | 13.8 | **12.3** | 12.4 | 7.00 | 7.30 | 15.0 | 0.80 | **8.49** | 0.340 | 0.180 | 0.280 |
| **14:1n7** | 0.70 | 1.30 | 0.90 | **0.96** | 0.40 | 0.30 | 1.40 | 0.40 | 1.40 | **0.780** | 0.40 | 0.30 | 2.10 | 0.70 | 0.40 | **0.79** | 0.990 | 0.740 | 0.660 |
| **16:1n7** | 1.40 | 0.00 | 0.20 | **0.50** | 0.00 | 1.70 | 0.60 | 2.10 | 1.90 | **1.24** | 0.90 | 1.00 | 1.10 | 0.00 | 0.00 | **0.59** | 0.210 | 0.850 | 0.280 |
| **18:1n7** | 1.50 | 0.00 | 0.00 | **0.50** | 0.30 | 1.60 | 1.00 | 1.30 | 0.10 | **0.850** | 0.70 | 0.20 | 1.60 | 1.80 | 0.70 | **1.02** | 0.710 | 0.380 | 0.530 |
| [**18:1n9**](http://www.lipomics.com/resources/fatty_acids/18_1n9.htm) | 23.3 | 19.0 | 26.1 | **22.8** | 15.0 | 21.1 | 16.9 | 20.5 | 32.2 | **21.1** | 16.4 | 18.6 | 53.1 | 24.2 | 15.9 | **25.6** | 0.570 | 0.770 | 0.710 |
| [**20:1n9**](http://www.lipomics.com/resources/fatty_acids/20_1n9.htm) | 0.60 | 0.10 | 0.00 | **0.27** | 0.30 | 0.60 | 0.80 | 0.50 | 0.00 | **0.44** | 0.10 | 0.20 | 1.50 | 0.70 | 0.30 | **0.57** | 0.660 | 0.450 | 0.480 |
| [**20:3n9**](http://www.lipomics.com/resources/fatty_acids/20_3n9.htm) | 0.10 | 0.00 | 8.30 | **2.80** | 0.00 | 0.00 | 0.10 | 0.00 | 0.00 | **0.030** | 0.1 | 0.00 | 0.10 | 0.00 | 0.00 | **0.05** | 0.270 | 0.220 | 0.220 |
| [**22:1n9**](http://www.lipomics.com/resources/fatty_acids/22_1n9.htm) | 2.60 | 1.30 | 0.00 | **1.31** | 0.20 | 1.30 | 2.90 | 0.00 | 1.80 | **1.25** | 1.60 | 0.9 | 1.30 | 0.00 | 0.30 | **0.83** | 0.520 | 0.510 | 0.950 |
| [**24:1n9**](http://www.lipomics.com/resources/fatty_acids/24_1n9.htm) | 27.0 | 4.30 | 16.3 | **15.9** | 0.80 | 37.4 | 28.6 | 22.5 | 9.60 | **19.8** | 21.6 | 5.90 | 5.60 | 29.9 | 0.70 | **12.8** | 0.440 | 0.740 | 0.710 |
| [**18:2n6**](http://www.lipomics.com/resources/fatty_acids/18_2n6.htm) | 13.1 | 5.60 | 5.50 | **8.07** | 5.10 | 13.8 | 6.30 | 10.5 | 5.60 | **8.26** | 7.70 | 5.40 | 11.7 | 12.3 | 6.00 | **8.62** | 0.880 | 0.840 | 0.950 |
| [**18:3n6**](http://www.lipomics.com/resources/fatty_acids/18_3n6.htm) | 0.30 | 0.00 | 0.10 | **0.15** | 0.00 | 0.20 | 0.20 | 0.00 | 0.00 | **0.080** | 0.00 | 0.00 | 0.00 | 0.80 | 0.00 | **0.15** | 0.630 | 0.990 | 0.450 |
| **20:2n6** | 0.00 | 1.10 | 7.00 | **2.70** | 0.30 | 0.40 | 1.60 | 0.50 | 0.00 | **0.540** | 0.60 | 0.00 | 0.70 | 0.70 | 0.30 | **0.47** | 0.830 | 0.210 | 0.230 |
| **20:3n6** | 0.30 | 0.70 | 1.60 | **0.89** | 0.20 | 0.20 | 0.50 | 0.40 | 1.60 | **0.600** | 0.50 | 0.60 | 0.70 | 0.60 | 0.30 | **0.54** | 0.810 | 0.290 | 0.560 |
| [**20:4n6**](http://www.lipomics.com/resources/fatty_acids/20_4n6.htm) | 7.50 | 1.60 | 3.90 | **4.35** | 0.00 | 7.20 | 4.30 | 4.60 | 10.6 | **5.34** | 4.50 | 1.90 | 2.90 | 7.20 | 0.10 | **3.31** | 0.370 | 0.630 | 0.720 |
| [**22:2n6**](http://www.lipomics.com/resources/fatty_acids/22_2n6.htm) | 0.40 | 0.00 | 0.00 | **0.130** | 0.00 | 0.50 | 0.30 | 0.20 | 0.00 | **0.200** | 0.60 | 0.00 | 0.00 | 0.20 | 0.00 | **0.190** | 0.920 | 0.790 | 0.660 |
| **22:4n6** | 0.40 | 0.50 | 0.00 | **0.300** | 0.10 | 0.40 | 0.40 | 0.40 | 4.20 | **1.09** | 0.40 | 0.30 | 0.60 | 0.80 | 0.10 | **0.450** | 0.440 | 0.470 | 0.480 |
| [**22:5n6**](http://www.lipomics.com/resources/fatty_acids/22_5n6.htm) | 1.40 | 0.70 | 1.80 | **1.29** | 0.50 | 1.40 | 0.40 | 1.20 | 1.80 | **1.05** | 1.10 | 0.70 | 2.00 | 0.00 | 0.30 | **0.830** | 0.640 | 0.410 | 0.580 |
| [**18:3n3**](http://www.lipomics.com/resources/fatty_acids/18_3n3.htm) | 0.50 | 0.00 | 0.30 | **0.260** | 0.70 | 0.40 | 0.00 | 1.10 | 0.10 | **0.460** | 0.00 | 0.30 | 0.70 | 1.10 | 2.60 | **0.93** | 0.370 | 0.310 | 0.520 |
| **18:4n3** | 0.50 | 0.00 | 0.00 | **0.170** | 0.00 | 1.80 | 0.00 | 0.00 | 0.00 | **0.360** | 0.00 | 0.00 | 0.00 | 0.00 | 0.20 | **0.05** | 0.410 | 0.420 | 0.710 |
| **20:3n3** | 0.00 | 0.00 | 0.00 | **0.00** | 0.00 | 0.00 | 0.00 | 0.00 | 0.00 | **0.00** | 0.00 | 0.00 | 0.00 | 0.00 | 0.00 | **0.00** | - | - | - |
| [**20:4n3**](http://www.lipomics.com/resources/fatty_acids/20_4n3.htm) | 0.50 | 0.30 | 1.90 | **0.900** | 0.20 | 0.40 | 0.50 | 0.50 | 0.40 | **0.410** | 0.60 | 0.30 | 0.80 | 0.30 | 0.10 | **0.44** | 0.830 | 0.290 | 0.230 |
| [**20:5n3**](http://www.lipomics.com/resources/fatty_acids/20_5n3.htm) | 3.90 | 0.00 | 0.00 | **1.31** | 1.90 | 0.40 | 0.00 | 0.00 | 0.00 | **0.460** | 0.00 | 0.50 | 3.70 | 0.00 | 0.90 | **1.03** | 0.490 | 0.840 | 0.460 |
| [**22:5n3**](http://www.lipomics.com/resources/fatty_acids/22_5n3.htm) | 3.00 | 0.70 | 6.30 | **3.30** | 0.00 | 6.50 | 4.00 | 2.30 | 1.90 | **2.95** | 2.40 | 0.60 | 0.60 | 3.70 | 0.10 | **1.47** | 0.280 | 0.260 | 0.860 |
| **22:6n3** | 0.00 | 0.80 | 1.10 | **0.640** | 0.10 | 6.00 | 3.70 | 4.10 | 1.70 | **3.12** | 4.60 | 0.80 | 1.60 | 6.30 | 0.40 | **2.74** | 0.810 | 0.230 | 0.120 |
| **24:6n3** | 0.00 | 0.00 | 0.00 | **0.00** | 0.00 | 0.00 | 0.00 | 0.00 | 0.00 | **0.00** | 0.00 | 0.00 | 0.00 | 0.00 | 0.00 | **0.00** | - | - | - |
| [**dm16:0**](http://www.lipomics.com/resources/fatty_acids/pl_16_0.htm) | 0.00 | 0.00 | 0.00 | **0.00** | 0.00 | 0.00 | 0.00 | 0.00 | 0.00 | **0.00** | 0.00 | 0.00 | 0.00 | 0.00 | 0.00 | **0.00** | - | - | - |
| [**dm18:0**](http://www.lipomics.com/resources/fatty_acids/pl_18_0.htm) | 0.00 | 0.00 | 0.00 | **0.00** | 0.00 | 0.00 | 0.00 | 0.00 | 0.00 | **0.00** | 0.00 | 0.00 | 0.00 | 0.00 | 0.00 | **0.00** | - | - | - |
| [**dm18:1n7**](http://www.lipomics.com/resources/fatty_acids/pl_18_1n7.htm) | 0.00 | 0.00 | 0.00 | **0.00** | 0.00 | 0.00 | 0.00 | 0.00 | 0.00 | **0.00** | 0.00 | 0.00 | 0.00 | 0.00 | 0.00 | **0.00** | - | - | - |
| [**dm18:1n9**](http://www.lipomics.com/resources/fatty_acids/pl_18_1n9.htm) | 0.00 | 0.00 | 0.00 | **0.00** | 0.00 | 0.00 | 0.00 | 0.00 | 0.00 | **0.00** | 0.00 | 0.00 | 0.00 | 0.00 | 0.00 | **0.00** | - | - | - |
| [**t16:1n7**](http://www.lipomics.com/resources/fatty_acids/t16_1n7.htm) | 1.20 | 0.10 | 0.00 | **0.430** | 0.30 | 1.50 | 1.30 | 1.30 | 0.00 | **0.890** | 1.80 | 0.30 | 0.20 | 2.20 | 0.40 | **0.980** | 0.860 | 0.410 | 0.390 |
| [**t18:1n9**](http://www.lipomics.com/resources/fatty_acids/t18_1n9.htm) | 0.00 | 0.00 | 0.00 | **0.00** | 0.00 | 0.00 | 0.00 | 0.00 | 0.00 | **0.00** | 0.00 | 0.00 | 0.00 | 0.00 | 0.00 | **0.00** | - | - | - |
| **t18:2n6** | 0.00 | 0.00 | 0.60 | **0.230** | 0.00 | 0.00 | 0.00 | 0.00 | 0.70 | **0.16** | 0.00 | 0.00 | 0.20 | 0.00 | 0.00 | **0.060** | 0.540 | 0.340 | 0.770 |
| **B Total Sphingomyelin and Fatty Acid Subclasses (nmol/g sample)^1^** | | | | | | | | | | | | | | | | | | | |
| **nmol FA/g** | 346 | 107 | 335 | **263** | 49.3 | 261 | 227 | 204 | 237 | **196** | 192 | 102 | 228 | 250 | 53.1 | **165** | 0.580 | 0.250 | 0.410 |
| **nmol CE/g** | 346 | 107 | 335 | **263** | 49.3 | 261 | 227 | 204 | 237 | **196** | 192 | 102 | 228 | 250 | 53.1 | **165** | 0.580 | 0.250 | 0.410 |
| **SFA** | 256 | 69.1 | 253 | **193** | 22.9 | 156 | 152 | 130 | 161 | **124** | 125 | 62.9 | 135 | 157 | 22.9 | **101** | 0.530 | 0.150 | 0.270 |
| **MUFA** | 57.0 | 26.1 | 43.5 | **42.2** | 17.0 | 64.0 | 52.2 | 47.2 | 47.0 | **45.5** | 41.8 | 27.2 | 66.4 | 57.4 | 18.4 | **42.2** | 0.790 | 1.00 | 0.800 |
| **PUFA** | 31.8 | 12.2 | 37.8 | **27.3** | 9.10 | 39.5 | 22.3 | 25.8 | 28.0 | **25.0** | 23.2 | 11.5 | 26.1 | 34.1 | 11.4 | **21.3** | 0.590 | 0.490 | 0.800 |
| **n3** | 8.30 | 1.80 | 9.60 | **6.58** | 3.00 | 15.5 | 8.20 | 8.10 | 4.10 | **7.77** | 7.60 | 2.60 | 7.40 | 11.4 | 4.30 | **6.66** | 0.690 | 0.980 | 0.740 |
| **n6** | 23.4 | 10.4 | 19.9 | **17.9** | 6.10 | 24.0 | 14.1 | 17.7 | 23.9 | **17.1** | 15.5 | 8.90 | 18.7 | 22.6 | 7.10 | **14.6** | 0.570 | 0.520 | 0.890 |
| **n7** | 2.80 | 0.00 | 0.20 | **1.01** | 0.30 | 3.30 | 1.60 | 3.30 | 2.00 | **2.10** | 1.60 | 1.20 | 2.70 | 1.80 | 0.70 | **1.61** | 0.480 | 0.490 | 0.320 |
| **n9** | 53.6 | 24.7 | 50.8 | **43.0** | 16.3 | 60.4 | 49.3 | 43.5 | 43.6 | **42.6** | 39.8 | 25.7 | 61.6 | 54.9 | 17.3 | **39.9** | 0.810 | 0.820 | 0.970 |
| **dm** | 0.00 | 0.00 | 0.00 | **0.00** | 0.00 | 0.00 | 0.00 | 0.00 | 0.00 | **0.00** | 0.00 | 0.00 | 0.00 | 0.00 | 0.00 | **0.00** | - | - | - |

Abbreviations: FA, fatty acid; SFA, saturated fatty acids; MUFA, mono unsaturated fatty acids; PUFA, Polyunsaturated fatty acids

^1^ Values were averaged and then rounded to 3 significant numbers. p values are derived from non-adjusted t-tests to assess trends. Values for p values of 0.1 or less are highlighted in bold. The data are for adult rats fed PQQ- or PQQ+ diets (n= 4 to 5 per group) and 3 additional rats fed the PQQ- diet; repleted with PQQ 4.5 mg/kg BW (PPQ-/+) for 3 days prior to assay.
